# Cytotoxic clerodane diterpenoids from the roots of *Casearia barteri* Mast.†‡

**DOI:** 10.1039/d4ra04393f

**Published:** 2024-07-22

**Authors:** Georges Bellier Tabekoueng, Franck Armand Fomo Fozing, Eduard Mas-Claret, Moses K. Langat, Marcel Frese, Achille Nouga Bissoue, Jean Duplex Wansi, Alain François Kamdem Waffo, Norbert Sewald, Bruno Ndjakou Lenta

**Affiliations:** a Department of Chemistry, Chemistry Laboratory, Faculty of Science, University of Douala P. O. Box 24157 Douala Cameroon; b Royal Botanic Gardens Kew TW9 3AE Richmond Surrey UK; c Department of Chemistry, Organic and Bioorganic Chemistry, Bielefeld University D-33501 Bielefeld Germany; d Department of Chemistry, Higher Teacher Training College, University of Yaoundé I P. O. Box 47 Yaoundé Cameroon

## Abstract

A study of diterpenoids as active ingredients against cancer from the active roots extract of *Casearia barteri* Mast. (IC_50_ = 1.57 μg mL^−1^) led to the isolation of six new clerodane diterpenoids, named as barterins A–F (1–6) alongside seven known compounds, caseamembrin A, caseamembrin E, casearlucin A, graveospene G, *N-trans*-feruloyltyramine, *N-cis*-feruloytyramine and sitosterol-3-*O*-β-*D*-(6-*O*-palmitoyl)-glucopyranoside. Their structures were elucidated based on NMR spectroscopic data and mass spectrometry. The absolute configurations of 1–6 were established by the time-dependent density functional theory (TDDFT), electronic circular dichroism (ECD) calculations and experimental data analysis. The cytotoxic effects of compounds 1–6 were evaluated against a human cervix carcinoma cell line *KB*-3-1. Barterins A–D (1–4) showed cytotoxic effects against the *KB*-3-1 cell line with IC_50_ values ranging from 1.34–4.73 μM.

## Abstract 2

A study of diterpenoids as active ingredients against cancer from the active roots extract of *Casearia barteri* Mast.

## Graphical abstract

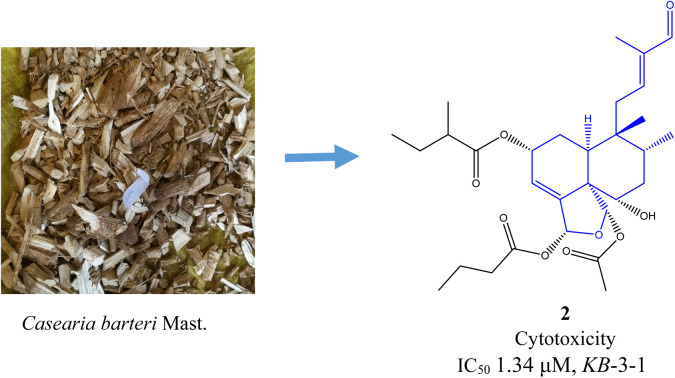

## Introduction

Natural products have been a cornerstone of drug discovery given their considerable chemical diversity, which has proven invaluable in the discovery of new anti-cancer agents.^1^ As the global cancer incidence continues to rise, the search for effective treatments remains a critical challenge. Among the promising candidates, clerodane-type diterpenes have been the subject of numerous works.^2–4^ Belonging to the family *Flacourtiaceae*, the genus *Casearia* includes a group of about 180 plant species widely spread in tropical and subtropical areas of Africa, Asia, Australia, North and South America and the Pacific islands.^5^ They are abundantly mentioned in traditional medicine for the treatment of diarrhea, skin lesions, ulceration, tropical leprosy, herpes, snake bite and fever.^6^ Numerous investigations on species of the genus *Casearia* Jacq. have revealed the occurrence of several classes of secondary metabolites, including clerodane-type diterpenes having a zuelanin and isozuelanin skeleton as the predominant type, with more than 150 already listed in the literature.^7–11^ Moreover, biological assays have revealed that these diterpenes possess cytotoxic, antibacterial, antifungal and DNA-modifying properties.^5^*Casearia barteri* Mast. is a small to medium-size tree, up to 20(−40) m high, present in tropical forest of Cameroon and Nigeria. The twigs and stem barks are chewed for sore gum and teeth cleaning.^12^ This high chemical and pharmacological potential as well as the contribution of the *Casearia* genus to traditional medicine motivated our choice of the study presented here.

## Results and discussion

**Fig. 1:**
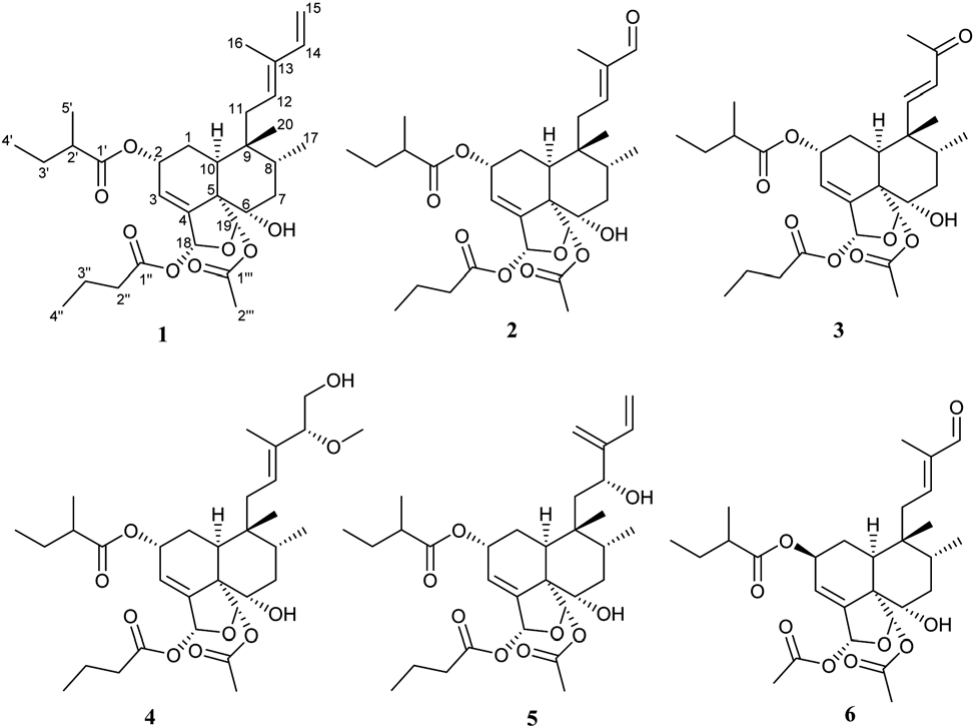
Structures of compounds 1–6 from *C. barteri*.

The crude extract derived from maceration of *C. barteri* roots in methanol led to further semi-pure extracts after fractionation with petrol ether–acetone solvent system of increasing polarity. These were purified using chromatographic columns with various stationary phases, leading to the isolation of thirteen compounds, including six new derivatives, barterins A–F (1–6) (Fig. 1) along with seven known compounds identified as caseamembrin A (7), caseamembrin E (8),^13^ the mixture of casearlucin A (9)^14^ and graveospene G (10),^11^*N-trans*-feruloyltyramine (11), *N-cis*-feruloytyramine (12),^15^ (−)-β-sitosterol-3-*O*-β-*D*-(6-*O*-palmitoyl)glucopyranoside (13)^16^ using their spectroscopic data by comparing with those reported in the literature.

### Structure elucidation

**Fig. 2:**
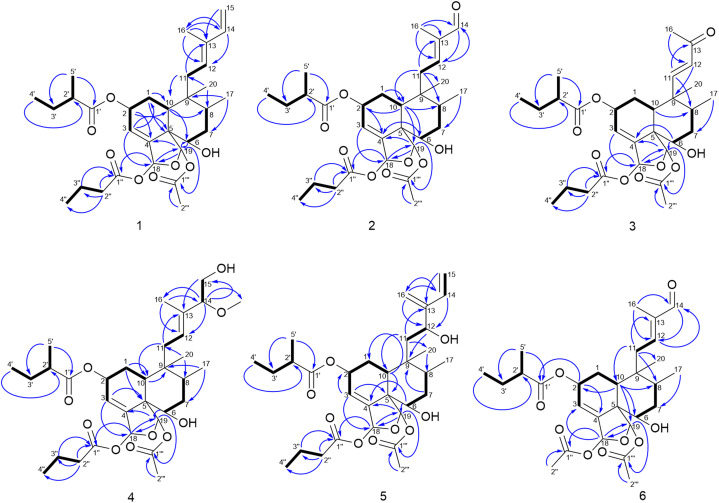
COSY and HMBC correlations of compounds 1–6.

**Fig. 3:**
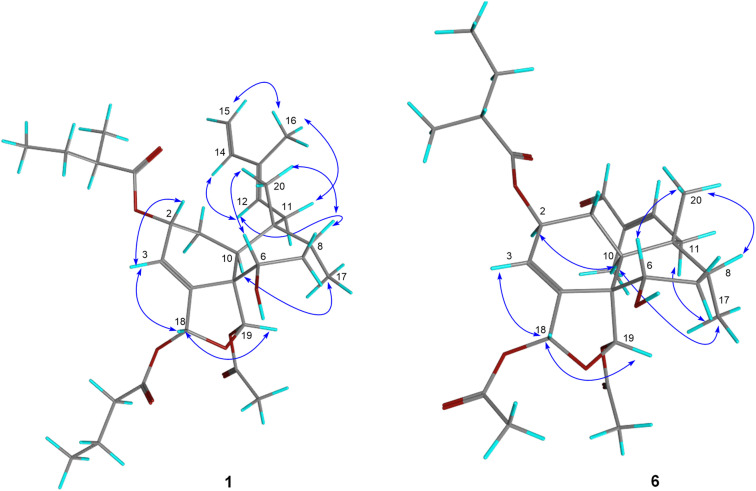
Key NOESY correlations of compounds 1 and 6.

**Fig. 4:**
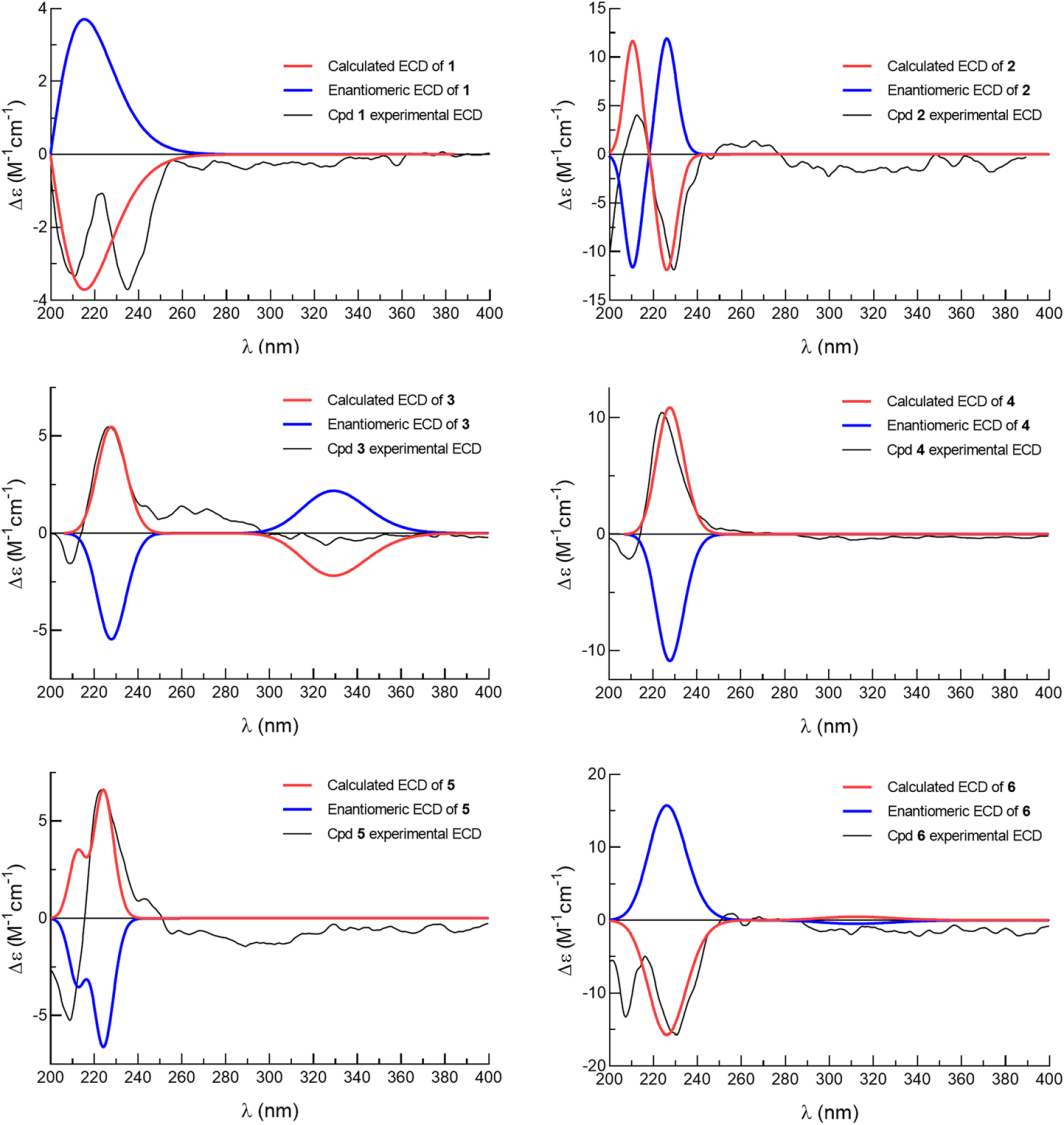
Calculated and experimental ECD spectra of compounds 1–6.

Compound 1 was isolated as a colorless oil. Its molecular formula was deduced as C_31_H_46_O_8_ by HRESIMS from the sodium adduct ion peak at *m*/*z* 569.3084 [M + Na]^+^ (calcd for C_31_H_46_O_8_Na, 569.3084) corresponding to nine double bond equivalents. The FTIR spectrum displayed the typical stretching bands of carbonyl functions at 1728 and 1749 cm^−1^ as well as a large absorption band due to the presence of hydroxyl groups centered at 3468 cm^−1^. The ^1^H NMR spectrum of 1 showed signals of seven methyl groups [*δ*_H_ 1.88 (s), 1.67 (s), 1.15 (d, *J* = 6.9 Hz), 0.95 (d, *J* = 7.4 Hz), 0.97 (t, *J* = 7.4 Hz), 0.92 (t, *J* = 7.4 Hz), 0.84 (s)], five olefinic protons [*δ*_H_ 5.99 (dt, *J* = 4.4, 1.4 Hz), 5.51 (d, *J* = 8.0 Hz), 6.35 (dd, *J* = 17.3, 10.8 Hz), 5.09 (d, *J* = 17.4 Hz), 4.91 (d, *J* = 10.7 Hz)], and three oxygenated methine protons [*δ*_H_ 5.40 (m), 6.69 (dd, *J* = 1.6, 0.6 Hz), 6.47 (br s)] (Table 1). The decoupled broadband ^13^C NMR spectrum of 1 exhibited resonances of 31 carbon atoms including those of 2-methylbutanoyloxy, butanoyloxy, and acetyloxy groups established from the observation of the following carbon signals (*δ*_C_ 175.8, 41.9, 27.7, 17.1, 12.1; 173.1, 36.7, 18.9, 13.9; 169.6, 21.9) (Table 2). The remaining 20 carbons have been assigned to those of the diterpene-type skeleton with the help of DEPT and HMQC spectra having three double bonds including one terminal olefin (*δ*_C_ 121.7, 147.5, 136.0, 130.9, 111.0; 142.5) as well two acetal carbons (*δ*_C_ 96.4, 98.2). From these data, we suggested that compound 1 may possess a zuelanin type skeleton, a subgroup of clerodane diterpenes previously described from *Casearia* genus.^17–19^ 2D homo- and hetero-nuclear correlations were further used to confirm the skeletal type of compound 1 and identify the position of its functional groups and substituents (Fig. 2). A tri-substituted double bound was located at position C-3/C-4 of the decalin ring system of diterpene from long range hetero-nuclear correlation of the proton signal at *δ*_H_ 5.99 (H-3) with carbons C-1 (*δ*_C_ 27.3), C-5 (*δ*_C_ 54.6) and C-18 (*δ*_C_ 96.4) and those of the proton at *δ*_H_ 5.40 (H-2) with C-10 (*δ*_C_ 37.7), C-3 (*δ*_C_ 121.7) and C-4 (*δ*_C_ 147.5). Similarly, a diene function was identified on the linear chain at C-12/C-13 and C-14/C-15 from correlations of the proton signal at *δ*_H_ 5.09 (H-15*Z*) with C-13 (*δ*_C_ 136.0) and C-14 (*δ*_C_ 142.5) and between the methyl signal at *δ*_H_ 1.67 (H-16) with the olefinic carbons C-12 (*δ*_C_ 130.9), C-13 (*δ*_C_ 136.0) and C-14 (*δ*_C_ 142.5). Furthermore, the butanoyl substituent was inferred to be attached at position C-18 from the ^3^*J* correlation between the proton signal at *δ*_H_ 6.69 (H-18) and the carbonyl at *δ*_C_ 173.1 (C-1′′). In the same way, the correlation between the proton signal at *δ*_H_ 6.47 (H-19) and the carbonyl at *δ*_C_ 169.6 (C-1′′′) was used to attach the acetoxyl group at position C-19. Following the same rationale, the 2-methylbutanoyloxy was attached at position C-2 considering the de-shielding effect of the proton H-2 (*δ*_H_ 5.40) attributable to the attractive mesomeric effect of the ester function, in agreement with data reported for other diterpenoids from the genus *Casearia* in the literature.^2,10^ An additional hydroxy group was located at C-6 using correlation between H-19 (*δ*_H_ 6.47) and C-6 (*δ*_C_ 72.9), and from H-10 (*δ*_H_ 2.44) to C-6 (*δ*_C_ 72.9). The *E*-configuration of the double bond C-12/C-13 as well as the transoid geometry of the conjugated double bond were established from NOESY interactions between H-11α,β/H-16/H-15 and H-8/H-12/H-14 and from the ^13^C NMR chemical shift of the allylic carbon C-11 at *δ*_C_ 31.1.^11^ Similarly, the relative configuration of the decalin ring system was discussed after examination of spatial correlations between H-17/H-10 and H-2/H-3/H-18/H-19/H-6/H-20/H-8. From these evidence, the decalin ring was established as *cis* fused with proton H-10 and methyl H-17 both α-oriented while protons H-2, H-6, H-18, H-19 and methyl H-20 adopted a β position (Fig. 3). The relative configuration (2*R*,5*S*,6*S*,8*R*,9*R*,10*S*,18*R*,19*S*) obtained from the analysis of spatial correlations was used as a model for the ECD calculations. The similarities between the negative Cotton effect at 215 nm of the experimental and theoretical ECD curves (Fig. 4) allow us to assign the absolute configuration 2*R*,5*S*,6*S*,8*R*,9*R*,10*S*,18*R*,19*S* for compound 1. These observations were in agreement with the stereochemistry of graveospene A.^11^

**Table 1:** ^1^H NMR data for compounds 1–6 (600 MHz, *δ* in ppm, *J* in Hz)

Position	1^a^	2^b^	3^b^	4^b^	5^b^	6^b^
1α	2.06 m	2.11 dd (14.7, 4.5)	2.02 m	2.04 m	2.00 m	2.25 m
1β	1.89 m	1.95 m	1.53 m	1.88 m	1.31 m	1.78 m
2	5.40 m	5.45 m	5.43 m	5.41 m	5.38 br d (4.5)	5.59 m
3	5.99 dt (4.4, 1.4)	6.02 d (3.4)	5.97 d (4.2)	6.02 br d (4.2)	5.99 d (4.1)	5.91 br s
4	—	—	—	—	—	—
5	—	—	—	—	—	—
6	3.89 ddd (11.7, 7.3, 5.0)	3.84 m	3.88 dd (10.7, 3.7)	3.84 m	3.78 br d (7.4)	4.00 dd (11.6, 4.8)
7	1.73 m	1.70 m	1.78 m	1.65 m	1.76 m	1.74 m
8	1.88 m	1.93 m	1.90 m	1.87 m	1.77 m	1.95 m
9	—	—	—	—	—	—
10	2.44 m	2.44 m	2.14 m	2.42 m	2.18 dd (14.8, 3.2)	2.54 dd (13.9, 2.7)
11α	2.30 m	2.53 dd (17.8, 8.4)	7.06 d (16.4)	2.31 m	1.76 m	2.48 dd (18.1, 8.1)
11β	1.75 m	1.99 m	—	1.52 m	1.41 m	1.96 m
12	5.51 d (8.0)	6.64 m	6.14 d (16.4)	5.51 m	4.43 br d (6.4)	6.80 br d (4.3)
13	—	—	—	—	—	—
14	6.35 dd (17.3, 10.8)	9.40 s	—	3.54 m	6.33 m	9.43 s
15	5.09 d (17.4), H_*Z*_	—	—	3.58 m	5.45 d (18.3)	—
15	4.91 d (10.7), H_*E*_	—	—	3.45 m	—	—
16	1.67 s	1.69 s	2.24 s	1.54 s	5.11 d (15.3)	1.66 s
17	0.95 d (7.4)	0.99 s	0.89 d (6.0)	0.96 d (6.0)	1.07 d (6.0)	0.98 d (7.0)
18	6.69 dd (1.6, 0.6)	6.71 br s	6.71 t (1.6)	6.71 t (1.6)	6.69 br s	6.64 t (1.5)
19	6.47 br s	6.52 s	6.32 s	6.52 s	6.37 s	6.47 s
20	0.84 s	0.94 s	1.16 s	0.82 s	1.08 s	0.96 s
1′	—	—	—	—	—	—
2′	2.42 m	2.48 sext (6.9)	2.42 m	2.44 m	2.40 ddd (13.7, 6.9, 1.7)	2.40 sext (6.9)
3′α	1.67 m	1.70 m	2.15 m	1.63 m	1.61 m	1.72 m
3′β	1.54 m	1.55 m	2.01 m	1.52 m	1.50 m	1.51 dt (13.7, 7.2)
4′	0.97 t (7.4)	0.97 s	0.98 t (7.5)	0.97 t (7.2)	0.95 t (7.5)	0.93 t (7.0)
5′	1.15 d (6.9)	1.18 d (7.0)	1.15 d (7.5)	1.17 d (7.0)	1.16 d (7.0)	1.16 d (7.0)
1′′	—	—	—	—	—	—
2′′	2.32 t (7.4)	2.33 t (7.4)	2.31 t (7.2)	2.34 t (7.2)	2.30 t (7.2)	2.07 s
3′′	1.61 sext (7.4)	1.65 sext (7.3)	1.63 sext (7.4)	1.66 sext (7.4)	1.62 sext (7.4)	—
4′′	0.92 t (7.4)	0.96 t (7.3)	0.96 t (7.4)	0.95 t (7.3)	0.94 t (7.3)	—
1′′′	—	—	—	—	—	—
2′′′	1.88 s	1.88 s	1.84 s	2.06 s	1.08 s	1.89 s
OMe	—	—	—	3.20, s	—	—

a Recorded in acetone-*d*_6_.

b Recorded in methanol-*d*_4_.

**Table 2:** ^13^C NMR Data for Compounds 1–6 (*δ* in ppm, 150 MHz)

Position	1^a^		2^b^		3^b^		4^b^		5^b^		6^b^	
1	27.3	CH_2_	27.6	CH_2_	28.0	CH_2_	27.6	CH_2_	27.6	CH_2_	27.3	CH_2_
2	67.2	CH	67.9	CH	67.7	CH	68.1	CH	68.1	CH	72.3	CH
3	121.7	CH	122.5	CH	122.2	CH	122.4	CH	122.3	CH	124.8	CH
4	147.5	C	147.4	C	147.3	C	147.7	C	147.7	C	146.3	C
5	54.6	C	54.9	C	54.9	C	55.2	C	55.4	C	54.9	C
6	72.9	CH	73.1	CH	72.9	CH	73.3	CH	73.6	CH	74.3	CH
7	38.0	CH_2_	37.9	CH_2_	37.7	CH_2_	38.1	CH_2_	38.0	CH_2_	38.2	CH_2_
8	37.3	CH	37.5	CH	36.2	CH	37.7	CH	38.3	CH	37.7	CH
9	38.5	C	39.0	C	42.4	C	39.3	C	39.6	C	39.5	C
10	37.7	CH	38.7	CH	43.0	CH	38.5	CH	42.0	CH	43.1	CH
11	31.1	CH_2_	32.9	CH_2_	154.6	CH	30.6	CH_2_	40.8	CH_2_	32.7	CH_2_
12	130.9	CH	153.5	CH	132.1	CH	128.1	CH	68.5	CH	154.1	CH
13	136.0	C	142.0	C	201.4	C	135.6	C	151.9	C	142.0	C
14	142.5	CH	196.1	CH	—	—	90.5	CH	138.2	CH	196.7	CH
15	111.0	CH_2_	—	—	—	—	64.8	CH_2_	115.2	CH_2_	—	—
16	12.0	CH_3_	9.5	CH_3_	27.1	CH_3_	11.7	CH_3_	114.7	CH_2_	9.3	CH_3_
17	15.9	CH_3_	16.0	CH_3_	16.5	CH_3_	16.1	CH_3_	16.4	CH_3_	15.9	CH_3_
18	96.4	CH	97.1	CH	97.3	CH	97.1	CH	96.9	CH	96.7	CH
19	98.2	CH	99.1	CH	98.7	CH	99.3	CH	99.7	CH	98.6	CH
20	25.4	CH_3_	25.3	CH_3_	24.9	CH_3_	25.6	CH_3_	24.9	CH_3_	25.2	CH_3_
1′	175.8	C	177.6	C	177.5	C	177.4	C	177.5	C	178.0	C
2′	41.9	CH	42.4	CH	42.5	CH	42.6	CH	42.6	CH	42.4	CH
3′	27.7	CH_2_	28.1	CH_2_	27.6	CH_2_	28.2	CH_2_	28.1	CH_2_	27.9	CH_2_
4′	12.1	CH_3_	12.1	CH_3_	12.1	CH_3_	12.2	CH_3_	12.1	CH_3_	11.9	CH_3_
5′	17.1	CH_3_	17.2	CH_3_	17.2	CH_3_	17.2	CH_3_	17.2	CH_3_	17.0	CH_3_
1′′	173.1	C	174.4	C	174.4	C	174.5	C	174.4	C	171.9	C
2′′	36.7	CH_2_	37.1	CH_2_	37.1	CH_2_	37.2	CH_2_	37.2	CH_2_	21.1	CH_3_
3′′	18.9	CH_2_	19.3	CH_2_	19.4	CH_2_	19.4	CH_2_	19.4	CH_2_	—	—
4′′	13.9	CH_3_	13.8	CH_3_	13.8	CH_3_	13.9	CH_3_	13.8	CH_3_	—	—
1′′′	169.6	C	170.8	C	171.2	C	171.1	C	171.4	C	171.0	C
2′′′	21.9	CH_3_	22.0	CH_3_	21.3	CH_3_	22.1	CH_3_	22.3	CH_3_	22.0	CH_3_
OMe	—		—		—	—	56.6	CH_3_	—	—	—	—

a Recorded in acetone-*d*_6_.

b Recorded in methanol-*d*_4_.

Compound 2 was purified as a colorless oil. The molecular formula of compound 2 was deduced to be C_30_H_44_O_9_ from the sodium adduct ion [M + Na]^+^ observed on its HRESIMS spectrum at *m*/*z* 571.2878 (calcd for C_30_H_44_NaO_9_, 571.2877) and its NMR data. The exhaustive analysis of the 1D NMR spectra revealed that compound 2 is analogous to compound 1, notably due to the presence of the same substituents 2-methylbutanoyloxy (*δ*_C_ 177.6, 42.4, 28.1, 17.2, 12.1), butanoyloxy (*δ*_C_ 174.4, 37.1, 19.3, 13.8) and acetyloxy (*δ*_C_ 170.8, 22.0) groups, as well the resonance of two acetal signals (*δ*_C_ 99.1, 97.1). Furthermore, it was established that the above-mentioned acyloxy groups as well as the hydroxyl function remain attached at positions 2, 18, 19 and 6, respectively, based on the observed HMBC and COSY correlations. The major difference is the absence of the resonance from the C-14/C-15 terminal double bond in conjunction with the appearance of a carbonyl signal (*δ*_C_ 196.1) attributable to an aldehyde function (*δ*_H_ 9.40). A putative oxidative cleavage of the double bond C-14/C-15 in 1 was thus suggested to establish the structure of compound 2.

The position of the aldehyde function at C-14 was further conclusive after interpretation of the HMBC correlations of its proton (*δ*_H_ 9.40, H-14) with olefinic and methyl carbons respectively at *δ*_C_ 153.5 (C-12), 142.0 (C-13) and 9.5 (C-16) as well as those between methyl H-16 with carbon C-12, C-13, C-14 (Fig. 2). The double bond C-12/C-13 was identified as *E*-configured according to the NOESY experiment where cross-peaks between H-11α,β/H-16 and H-14/H-12/H-17 were observed. Futher NOESY interaction between H-8/H-18/H-19 revealed the same relative configuration as found for compound 1. Positive Cotton effects at 222 nm and 276 nm matched the simulated ECD curve (Fig. 4) and allow to assign the absolute configuration of compound 2 as 2*R*,5*S*,6*S*,8*R*,9*R*,10*S*,18*R*,19*S*, matching that of compound 1. Compound 3 was obtained as a colorless oil. Its HRESIMS showed a sodium adduct pic at *m*/*z* 5557.2719 [M + Na]^+^ (calcd for C_29_H_42_NaO_9_, 557.2721), corresponding to a molecular formula C_29_H_42_NaO_9_, bearing nine double bond equivalents. From the ^1^H and ^13^C NMR spectra, several similarities to compound 1 were apparent. Indeed, the 2-methylbutanoyloxy (*δ*_C_ 177.5, 42.5, 27.6, 17.2, 12.1), butanoyloxy (*δ*_C_ 174.4, 37.1, 19.4, 13.8), and acetyloxy (*δ*_C_ 171.2, 21.3) groups, as well as the resonance of two acetal signals (*δ*_C_ 98.7, 97.3) were evidenced. Furthermore, the position of these acyloxy groups as well as the hydroxyl function on the clerodane-type diterpene skeleton was identified by means of the HMBC spectrum in which correlations between the protons at *δ*_H_ 5.43 (H-2), 6.71 (H-18) and 6.32 (H-19) with the carbonyl carbons at *δ*_C_ 177.5, 174.4, 171.2 and 72.9 demonstrated the 2-methylbutanoyloxy, butanoyloxy, acetoxy and hydroxy groups to be attached at C-2, C-18, C-19 and C-6 respectively. Beside these similarities with compound 1, close analysis of the 1 and 2D NMR spectra indicated a difference in the side chain attached at position 9 of ring B. Indeed, resonances of an α,β-unsaturated methyl ketone at *δ*_C_ 201.4 (C-13), 154.6 (C-11), 132.1 (C-12), 27.1 (C-16) were observed and further corroborated from HMBC correlations between the methyl protons at *δ*_H_ 2.24 (H-16) with carbons C-13 and C-12 and also between the olefinic proton at *δ*_H_ 7.06 (H-11) and carbons C-13 and C-12. The stereochemistry of the double bond C-11/C-12 was inferred as *trans* from the coupling constant of both proton H-11 and H-12 (d, *J* = 16.4 Hz). The NOESY experiment disclosed cross-peaks between H-18/H-19/H-6/H-8/H-20 and H-10/H-17 as well as H-16/H-11α,β and H-12/H-20/H-2/H-6, in agreement with those found for compound 1. The calculated ECD curve for the stereoisomer 2*R*,5*S*,6*S*,8*R*,9*R*,10*S*,18*R*,19*S* displays the same profile as the experimental one (Fig. 4) and this unambiguously led to the assignment of the absolute configuration for compound 3.

Compound 4 was also isolated as a brownish oil. Its HRESIMS in positive mode having the sodium adduct peak at 617.3292 [M + Na]^+^ (calcd for 617.3296 for C_32_H_50_O_10_Na), suggested a molecular formula of C_32_H_50_O_10_, corresponding to eight degrees of unsaturation. NMR data comparison revealed that compound 4 was similar to 1, except for the presence of a methoxy (*δ*_H_ 3.20/*δ*_C_ 56.6), an oxymethylene (*δ*_H_ 3.58, 3.45/*δ*_C_ 64.8) and an oxymethine group (*δ*_H_ 3.54/*δ*_C_ 90.5) together with the loss of the signals from the terminal double bond C-14/C-15. Strong ^1^H–^1^H ^3^*J* COSY correlations were observed between the oxymethine and the oxymethylene protons and their position at C-14 and C-15 were inferred from HMBC cross-peaks from the methyl protons at *δ*_H_ 1.54 (H-16) to carbons C-12 (*δ*_C_ 128.1), C-13 (*δ*_C_ 135.6) and C-14 (*δ*_C_ 90.5) and between the oxymethine proton at *δ*_H_ 3.58 (H-14) and carbon C-12, C-15. Furthermore, correlations of the oxymethine proton H-14 with the methoxy carbon at *δ*_C_ 56.6 evidenced the position of the methoxy at C-14. The same relative configuration for compound 4 as that of 1 was corroborated from H-20/H-8/H-11α/H-16/H-15α,β/MeO-14, H-2/H-3/H-18/H-19/H-6/H-1α. Similarly, the orientation of the proton H-14 in β position was deduced from NOESY cross peaks from H-12/H-20/H-14. The experimental ECD curve was consistent with the calculated ECD (Fig. 4) for the configuration 2*R*,5*S*,6*S*,8*R*,9*R*,10*S*,14*R*,18*R*,19*S* establishing thus the absolute configuration of compound 4.

Compound 5, obtained as a brownish oil with a molecular formula C_31_H_46_O_9_ based on the presence of a HRESIMS ion peak at *m*/*z* 585.3035 [M + Na]^+^ (calcd for C_31_H_46_NaO_9_, 585.3034). Compound 5 contains nine double bond equivalents and is like compound 1 described above with respect to the number of carbon atoms, but with an additional oxygen atom. In addition to the signals related to the identical acyl substituents present, a comparative analysis established that the basic skeleton of compound 5 belongs to isozuelanin, with two terminal double bonds at the level of the lateral chain of clerodane-type diterpenes. Indeed, the DEPT 135 of compound 5 spectrum exhibited resonances of two olefinic methylene carbons at *δ*_C_ 114.7 and 115.2. The typical β-monosubstituted diene of the isozuelanin skeleton was confirmed by interpretation of the HMBC correlations of both terminal protons at *δ*_H_ 5.11 (H-16) and 5.45 (H-15) with olefinic carbons at at *δ*_C_ 151.9 (C-13) and 138.2 (C-14). Beyond this aspect of position isomerism, compound 5 was found to have an additional oxygen atom as outlined above, which resulted in the presence of an additional hydroxyl group compared to 1. Based on HMBC cross-peaks of protons H-16 and H-14 with carbon C-12, it was assigned unambiguously at position C-12. The cisoid configuration of the diene was deduced from the NOESY spatial correlation between proton H-15 and H-16. Similarly, the relative configuration of compound 5 was shown identical as compound 1 from analysis of spatial correlations with a β-oriented proton H-12 inferred from NOESY correlation between H-12 and H-8. This was further confirmed with the similar Cotton effect at 223 nm on both experimental and calculated ECD curve for the stereoisomer 2*R*,5*S*,6*S*,8*R*,9*R*,10*S*,12*R*,18*R*,19*S* (Fig. 4).

The molecular formula, C_28_H_40_O_9_, of compound 6 that was obtained as a colorless oil, could be inferred from the relevant HRESIMS ion peak at *m*/*z* 543.2557 [M + Na]^+^ (calcd for C_28_H_40_NaO_9_, 543.2564). Analysis of the 1D NMR spectra revealed that 6 had the same clerodane-like structure with three substituent groups, notably, a 2-methylbutanoyloxy group (*δ*_C_ 178.0, 42.4, 27.9, 11.9, 17.0) and two acetyloxy groups (171.9, 21.1, 171.0, 22.0). These conclusions were supported by analysis of the 2D NMR spectra crosspeaks. ^3^*J* correlation of H-2 to the carbonyl group (*δ*_C_ 178.0) demonstrated that the 2-methylbutyryloxy unit was positioned at C-2. Similarly, both acetyloxy placed at C-18 and C-19 were positioned from the corresponding HMBC couplings of protons H-18 (*δ*_H_ 6.64) and proton H-19 (*δ*_H_ 6.47) with ester carbonyls at *δ*_C_ 171.9 and 171.0. On the same basis, the hydroxy methine was placed at position 6 as it was for the compounds described above, based on the HMBC correlation between the proton H-19 and carbon at *δ*_C_ 74.3 (C-6). The presence of the aldehyde function at *δ*_C_ 196.7 brought us to compare the HMBC correlations of compound 6 to those of compound 2. Indeed, this allowed us to identify the aldehyde at the same position C-14, notably through the correlations from proton H-14 to olefinic carbons C-12, C-13 and methyl C-16. It has, therefore, been established that compound 6 is distinct from compound 2 by the nature of the acyloxy substituent in position 2, specifically the 2-methylbutanoyloxy group replaced by the acetyloxy group. Furthermore, the spatial correlation of H-2 and H-10, H-17 were in agreement with an α orientation of H-2 unlike the β orientation it occupies in compound 1 (Fig. 3). The absolute configuration 2*S*,5*S*,6*S*,8*R*,9*R*,10*S*,18*R*,19*S* was inferred from the comparison between theoretical and experimental ECD curves, with a negative Cotton effect at 228 nm (Fig. 4).

### Cytotoxic activity

Cervix cancer is the fourth most diagnosed cancer in women worldwide, with more than half a million new cases and 311 365 deaths in 2018, with nine-tenths of these being among women living in low- and middle-income countries.^20^ A large number of plants and plant-derived principles has demonstrated anticancer activity through selective cytotoxicity towards tumour cells. To bring our contribution to the search for new anti-cancer agents, we evaluated the cytotoxic potential of diterpenes 1–6 and the crude extract towards the cervix carcinoma cell line *KB*-3-1 (a HeLa subclone) with griseofulvin as standard and using the MTT assay as previously described.^21^ The crude extract exhibited strong cytotoxic activity with IC_50_ of 1.58 μg mL^−1^, in agreement with the range defined by the National Cancer Institute in US, which considers an extract to be active when the IC_50_ value after incubation between 48 and 72 h, is less than 20 μg mL^−1^.^22^ This corroborates the results obtained on other cancer cell lines such as mouse P388 lymphocytic leukemia cell line where extracts from various parts of *C. costulata*, *C. grayi*, *C. multinervosa*, *C. grewiifolia* have shown similar activity with IC_50_ ranging from 0.89 to 4.2 μg mL^−1^,^23^ human breast adenocarcinoma cell line MCF-7 where the ethyl acetate leave extract of *C. capitellata* exhibited potent cytotoxicity (IC_50_ = 2.0 μg mL^−1^). However, few studies have been done towards cervix cancer cell lines, such as the one performed by Silva, which indicated a moderate activity of the essential oil of *C. sylvestris* towards HeLa cells with IC_50_ = 63.3 μg mL^−1^.^24^ Compounds 1, 2, 3 and 6 also had pronounced activity with IC_50_ values of 2.52 μM, 1.34 μM, 4.73 μM, and 1.54 μM, respectively, compared to the control griseofulvin (IC_50_ = 19.3 μM) while compounds 4 and 5 were inactive (IC_50_ > 200 μM) (Table 3). It is noteworthy that these fulfil one of the criteria attributed to potential plant-derived anti-cancer candidates with an IC_50_ value less than 10 μM.^25^ The high activity of compounds 2 and 6 could be related to the aldehyde function at position 14. However, further analysis needs to be carried out to better elucidate the influence of this chemical function, as no bioassay results or computational analysis on the cytotoxic activity of analogue compounds is available in the literature.

**Table 3:** Cytotoxic activities

Samples	IC_50_
Crude extract	1.58 μg mL^−1^
Barterin A (1)	2.52 μM
Barterin B (2)	1.34 μM
Barterin C (3)	4.73 μM
Barterin D (4)	>200 μM
Barterin E (5)	>200 μM
Barterin F (6)	1.54 μM

## Experimental section

### General experimental procedures

UV spectra were obtained using a Hitachi UV 3200 spectrophotometer, and IR spectra with a using JASCO 302-A spectrometer (Thermo Scientific, Waltham, MA, USA). ECD spectra were obtained on a JASCO J-715CD spectrometer (JASCO Corporation, Tokyo, Japan). Optical rotations were measured on a JASCO DIP-3600 digital polarimeter (JASCO, Tokyo, Japan) at 23 °C. ^1^H, ^13^C and 2D NMR spectra were recorded at room temperature using a Bruker DRX-600 spectrometer (125 MHz for ^13^C and 500 MHz for ^1^H, Bruker, Germany) to provide chemical shifts that are expressed in ppm relative to tetramethylsilane (TMS). ESI-Mass spectra were obtained with an Agilent 6220 TOF LCMS mass spectrometer (Agilent Technologies, Santa Clara, CA, USA). Column chromatography was performed using silica gel of 70–230 mesh (Merck, Darmstadt, Germany) and aluminum plates precoated with silica gel 60 F254 (Merck, Darmstadt, Germany) were used for thin layer chromatography. TLC spots were visualized under UV light at 254 or 365 nm, followed by spraying with 20% aqueous H_2_SO_4_ spray and heating. Normal phase medium pressure liquid chromatography (MPLC) was run using Reveleris X2 Flash Chromatography System equipped with an UV-vis and ELSD detectors (Buchi, Switzerland).

### Plant material

The roots of *Casearia barteri* Mast. were collected in March 2016 from Bangangté, West Province, Cameroon. The botanical identification was made by the botanist Mr Nana Victor at the National Herbarium of Cameroon, where a voucher specimen (no. 65823 HNC) were already available.

### Extraction and isolation

The air-dried roots of *Casearia barteri* (1.86 kg) were powdered and macerated twice with methanol (2 × 10 L) during 48 H at room temperature. The filtrate was then evaporated using rotary evaporator under reduced pressure to afford a crude extract (77.1 g). The obtained extract was dissolved and fixed onto silica gel and subsequently fractioned using open silica gel column 230–400 mesh (Merck, Darmstadt, Germany) to yield F_1_ (PE, 23.6 mg), F_2_ (PE/acetone 1 : 1 v/v, 28.5 g), F_3_ (acetone, 19.3 g), F_4_ (acetone/MeOH 1 : 1 v/v, 10.2 g), F_5_ (MeOH, 15.9 g) on the basis of their TLC profiles. Fraction was F_2_ divided into two parts and separated by normal phase MPLC over silica gel eluting with a step gradient PE-acetone (5 : 95, 10 : 90, 15 : 85, 20 : 80, 30 : 70, 40 : 60, 50 : 50 v/v) to afford seven subfractions (F_2−1_–F_2−7_). F_2−1_ was purified using open column loaded with silica gel and eluted with DCM : MeOH (99 : 1) to yield the mixture of two inseparable compounds 9 + 10 (6.3 mg). Further elution on isocratic mode (DCM/MeOH (95 : 5)) of F_2−2_ over column silica gel let to partially purified mixtures labelled F_2−2-A_–F_2−2-F_ that were purified repeatedly over sephadex column eluted with DCM/MeOH (1 : 1) to afford compound 1 (4.2 mg), 2 (5.9 mg), 3 (3.9 mg) and 7 (8.2 mg). Following the same procedure, F_2−3_ to F_2−5_ were treated separately to afford compound 4 (DCM/MeOH (85 : 15), 2.2 mg), 5 (DCM/MeOH (90 : 10), 3.1 mg), 6 (DCM/MeOH (90 : 10), 3.2 mg), and 8 (DCM/MeOH (90 : 10), 8.2 mg). F_2−6_ was purified over column silica gel followed with sephadex column eluted with methanol yielded compound 11 (DCM/MeOH (80 : 20), 5.2 mg) and 12 (DCM/MeOH (80 : 20), 6.3 mg). Compound 13 (DCM/MeOH (90 : 10), 3.1 mg) was obtained after elution of fraction F_3_ with DCM/MeOH (70 : 30) over silica gel column.

Barterin A (1): colorless oil; [*α*]^20^_D_ + 32.1 (*c* = 1.0, MeOH); ECD (CH_3_CN) 210 (Δ*ε* − 3.6), 240 (Δ*ε* − 3.9) nm; IR (film) *ν*_max_ 2965, 2933, 1748, 1721, 1459, 1370, 1221, 1170, 946, 735 cm^−1^; ^1^H NMR (600 MHz, acetone-*d*_6_) and ^13^C NMR (150 MHz, acetone-*d*_6_) data, see Tables 1 and 2; HRESIMS *m*/*z* 569.3084 [M + Na]^+^ (calcd for C_31_H_46_O_8_Na, 569.3084).

Barterin B (2): colorless oil; [*α*]^20^_D_ + 17.5 (*c* = 2.0, MeOH); ECD (CH_3_CN) 212 (Δ*ε* − 2.2), 245 (Δ*ε* − 2.1) nm; IR (film) *ν*_max_ 2965, 2930, 1749, 1728, 1369, 1222, 1172, 1062, 947 cm^−1^; ^1^H NMR (600 MHz, methanol-*d*_4_) and ^13^C NMR (150 MHz, methanol-*d*_6_) data, see Tables 1 and 2; HRESIMS *m*/*z* 571.2878 [M + Na]^+^ (calcd for C_30_H_44_NaO_9_, 571.2877).

Barterin C (3): colorless oil; [*α*]^20^_D_ − 5.4 (*c* = 1.0, MeOH); ECD (CH_3_CN) 209 (Δ*ε* − 2.8), 232 (Δ*ε* − 3.6) nm; IR (film) *ν*_max_ 2965, 2933, 1748, 1721, 1459, 1370, 1221, 1170, 946, 735 cm^−1^; ^1^H NMR (600 MHz, methanol-*d*_4_) and ^13^C NMR (150 MHz, methanol-*d*_6_) data, see Tables 1 and 2; HRESIMS *m*/*z* 5557.2719 [M + Na]^+^ (calcd for C_29_H_42_NaO_9_, 557.2721).

Barterin D (4): colorless oil; [*α*]^20^_D_ + 62.7 (*c* = 1.0, MeOH); ECD (CH_3_CN) 230 (Δ*ε* + 8.9) nm; IR (film) *ν*_max_ 2964, 2929, 1749, 1730, 1457, 1372, 1225, 1147, 1063, 1010, 947, 669 cm^−1^; ^1^H NMR (600 MHz, methanol-*d*_4_) and ^13^C NMR (150 MHz, methanol-*d*_6_) data, see Tables 1 and 2; HRESIMS *m*/*z* 617.3292 [M + Na]^+^ (calcd for 617.3296 for C_32_H_50_O_10_Na).

Barterin E (5): colorless oil; [*α*]^20^_D_ + 22.9 (*c* = 2.0, MeOH); ECD (CH_3_CN) 210 (Δ*ε* − 1.8), 241 (Δ*ε* + 2.1) nm; IR (film) *ν*_max_ 2961, 2924, 1728, 1373, 1226, 1061, 946, 669 cm^−1^; ^1^H NMR (600 MHz, methanol-*d*_6_) and ^13^C NMR (150 MHz, methanol-*d*_4_) data, see Tables 1 and 2; HRESIMS *m*/*z* 585.3035 [M + Na]^+^ (calcd for C_31_H_46_NaO_9_, 585.3034).

Barterin F (6): colorless oil; [*α*]^20^_D_ − 66.2 (*c* = 2.0, MeOH); ECD (CH_3_CN) 210 (Δ*ε* − 3.6), 240 (Δ*ε* − 3.9) nm; IR (film) *ν*_max_ 2968, 2929, 1751, 1732, 1685, 1372, 1229, 1179, 1003, 962 cm^−1^; ^1^H NMR (600 MHz, methanol-*d*_4_) and ^13^C NMR (150 MHz, methanol-*d*_4_) data, see Tables 1 and 2; HRESIMS *m*/*z* 543.2557 [M + Na]^+^ (calcd for C_28_H_40_NaO_9_, 543.2564).

### ECD calculations

The calculated ECD spectra of new compounds were performed as previously reported.^26,27^ Systematic conformational searches were performed firstly using MOE software and conformers under 3.0 kcal mol^−1^ were optimized using the DFT method at the B3LYP/6-31+G(d,p) level (Gaussian 09).^28^ A second optimization was performed using the DFT method at the B3LYP/6-311+G(d,p) level, and a polarizable continuum model (IEFPCM, solvent: acetonitrile) was applied to mimic the effects of the solvent used in the experimental ECD spectra. Theoretical ECD spectra were simulated for those conformers falling above 1% population threshold after applying Boltzmann distribution. Time Dependant Density Functional Theory (TDDFT) was used at the CAM-B3LYP/6-31+G(d) level of theory (IEFPCM, solvent: acetonitrile). The ECD curves were extracted using SpecDis 1.61 software.^29^ The overall ECD curves of all the compounds were weighted by Boltzmann distribution after UV correction.

## Conclusions

Six new clerodane-type diterpenoids, barterins 1–6 as well as four knowns were described from the roots extract of *Casearia barteri*. These isolates were found to share the same skeleton as the previous ones reported from the genus *Casearia*, enhancing their character as chemotaxonomic markers of this genus. Besides, their structural diversity, the reported diterpenoids are also endowed with significant cytotoxic activity, such as barterin B and E which presented the pronounced inhibition activity (IC_50_ = 1.34, 1.54 μM respectively) while the crude extract exhibited also a strong activity (IC_50_ = 1.58 μg mL^−1^). These observations suggest that *Casearia barteri* should be given considerable attention, especially to better understand the mode of action in the perspective of the search for alternative treatment for cervical cancer.

## Data availability

The authors declare that the data supporting the findings of this study are available within the paper and ESI.‡

## Conflicts of interest

The authors declare no conflict of interest that could have appeared to influence the work reported in this paper.

## Supplementary Material

RA-014-D4RA04393F-s001
