# Supplementary material for: Cytotoxic clerodane diterpenoids from the roots of Casearia barteri Mast
Source: RSC Adv. 2024 Jul 22;14(32):23109–17. doi: 10.1039/d4ra04393f (PMC11262006; doi:10.1039/d4ra04393f)
Supplement: RA-014-D4RA04393F-s001 [file RA-014-D4RA04393F-s001.pdf]

Electronic Supplementary Material (ESI) for RSC Advances.
This journal is © The Royal Society of Chemistry 2024

Cytotoxic clerodane diterpenoids from the roots of *Casearia barteri* Mast.

Georges Bellier Tabekoueng,[*a] Franck Armand Fomo Fozing,[a] Eduard Mas-Claret,[b] Moses K. Langat,[*b] Marcel Frese,[c] Achille Nouga Bissoue,[a] Jean Duplex Wansi,[a] Alain François Kamdem Waffo,[a] Norbert Sewald[c] and Bruno Ndjakou Lenta[*d]

[a]*Chemistry Laboratory, Department of Chemistry, Faculty of Science, University of Douala, P.O. Box 24157, Douala, Cameroon*

[b]*Royal Botanic Gardens Kew, Richmond, TW9 3AE, Surrey, UK*

[c]*Organic and Bioorganic Chemistry, Department of Chemistry, Bielefeld University, D-33501 Bielefeld, Germany*

[d]*Department of Chemistry, Higher Teacher Training College, University of Yaoundé I, P. O. Box 47, Yaoundé, Cameroon*

In memory of Professor Juliette Catherine Vardamides, University of Douala-Cameroon.

*Corresponding authors

E-mail addresses: bellier_tab@yahoo.fr (G.B.T.), lentabruno@yahoo.fr (B.N.L.), m.langat@kew.org (M.K.L.)

Supporting material

Figure 1:	ESI-HR-MS (+) spectrum of compound 1	5
Figure 2:	FT-IR spectrum of compound 1	6
Figure 3:	^{1}H NMR spectrum (500 MHz, acetone-d_6) of compound 1	7
Figure 4:	^{13}C NMR spectrum (125 MHz, acetone-d_6) of compound 1	8
Figure 5:	DEPT 135 NMR spectrum (125 MHz, acetone-d_6) of compound 1	9
Figure 6:	COSY spectrum (500 MHz, acetone-d_6) of compound 1	10
Figure 7:	HSQC spectrum (500 MHz, acetone-d_6) of compound 1	11
Figure 8:	HMBC spectrum (500 MHz, acetone-d_6) of compound 1	12
Figure 9:	NOESY spectrum (500 MHz, acetone-d_6) of compound 1	13
Figure 10:	ESI-HR-MS (+) spectrum of compound 2	14
Figure 11:	FT-IR spectrum of compound 2	15
Figure 12:	^{1}H NMR spectrum (500 MHz, methanol-d_4) of compound 2	16
Figure 13:	^{13}C NMR spectrum (125 MHz, methanol-d_4) of compound 2	17
Figure 14:	DEPT 135 NMR spectrum (125 MHz, methanol-d_4) of compound 2	18
Figure 15:	COSY spectrum (500 MHz, methanol-d_4) of compound 2	19
Figure 16:	HSQC spectrum (500 MHz, methanol-d_4) of compound 2	20
Figure 17:	HMBC spectrum (500 MHz, methanol-d_4) of compound 2	21
Figure 18:	NOESY spectrum (500 MHz, methanol-d_4) of compound 2	22
Figure 19:	ESI-HR-MS (+) spectrum of compound 3	23
Figure 20:	FT-IR spectrum of compound 3	24

Figure 21: ^{1}H NMR spectrum (500 MHz, methanol-d_4) of compound 3 25
Figure 22: ^{13}C NMR spectrum (125 MHz, methanol-d_4) of compound 3 26
Figure 23: DEPT 135 NMR spectrum (125 MHz, methanol-d_4) of compound 3 27
Figure 24: COSY spectrum (500 MHz, methanol-d_4) of compound 3 28
Figure 25: HSQC spectrum (500 MHz, methanol-d_4) of compound 3 29
Figure 26: HMBC spectrum (500 MHz, methanol-d_4) of compound 3 30
Figure 27: NOESY spectrum (500 MHz, methanol-d_4) of compound 3 31
Figure 28: ESI-HR-MS (+) spectrum of compound 4 32
Figure 29: FT-IR spectrum of compound 4 33
Figure 30: ^{1}H NMR spectrum (500 MHz, methanol-d_4) of compound 4 34
Figure 31: ^{13}C NMR spectrum (125 MHz, methanol-d_4) of compound 4 35
Figure 32: DEPT 135 NMR spectrum (125 MHz, methanol-d_4) of compound 4 36
Figure 33: COSY spectrum (500 MHz, methanol-d_4) of compound 4 37
Figure 34: HSQC spectrum (500 MHz, methanol-d_4) of compound 4 38
Figure 35: HMBC spectrum (500 MHz, methanol-d_4) of compound 4 39
Figure 36: NOESY spectrum (500 MHz, methanol-d_4) of compound 4 40
Figure 37: ESI-HR-MS (+) spectrum of compound 5 41
Figure 38: FT-IR spectrum of compound 5 42
Figure 39: ^{1}H NMR spectrum (500 MHz, methanol-d_4) of compound 5 43
Figure 40: ^{13}C NMR spectrum (125 MHz, methanol-d_4) of compound 5 44
Figure 41: DEPT 135 NMR spectrum (125 MHz, methanol-d_4) of compound 5 45

Figure 42: COSY spectrum (500 MHz, methanol-d_4) of compound 5 46
Figure 43: HSQC spectrum (500 MHz, methanol-d_4) of compound 5 47
Figure 44: HMBC spectrum (500 MHz, methanol-d_4) of compound 5 48
Figure 45: NOESY spectrum (500 MHz, methanol-d_4) of compound 5 49
Figure 46: ESI-HR-MS (+) spectrum of compound 6 50
Figure 47: FT-IR spectrum of compound 6 51
Figure 48: ^{1}H NMR spectrum (500 MHz, methanol-d_4) of compound 6 52
Figure 49: ^{13}C NMR spectrum (125 MHz, methanol-d_4) of compound 6 53
Figure 50: DEPT 135 NMR spectrum (125 MHz, methanol-d_4) of compound 6 54
Figure 51: COSY spectrum (500 MHz, methanol-d_4) of compound 6 55
Figure 52: HSQC spectrum (500 MHz, methanol-d_4) of compound 6 56
Figure 53: HMBC spectrum (500 MHz, methanol-d_4) of compound 6 57
Figure 54: NOESY spectrum (500 MHz, methanol-d_4) of compound 6 58
Figure 55: Dose-response curves 60
Figure 56: Low-energy (≥ 1%) conformers of compound 1 obtained at B3LYP/6-311+G(d,p) IEFPCM/MeCN level of theory 61
Figure 57: Low-energy (≥ 1%) conformers of compound 2 obtained at B3LYP/6-311+G(d,p) IEFPCM/MeCN level of theory 62
Figure 58: Low-energy (≥ 1%) conformers of compound 3 obtained at B3LYP/6-311+G(d,p) IEFPCM/MeCN level of theory 63
Figure 59: Low-energy (≥ 1%) conformers of compound 4 obtained at B3LYP/6-311+G(d,p) IEFPCM/MeCN level of theory 64
Figure 60: Low-energy (≥ 1%) conformers of compound 5 obtained at B3LYP/6-311+G(d,p) IEFPCM/MeCN level of theory 65
Figure 61: Low-energy (≥ 1%) conformers of compound 6 obtained at B3LYP/6-311+G(d,p) IEFPCM/MeCN level of theory 66

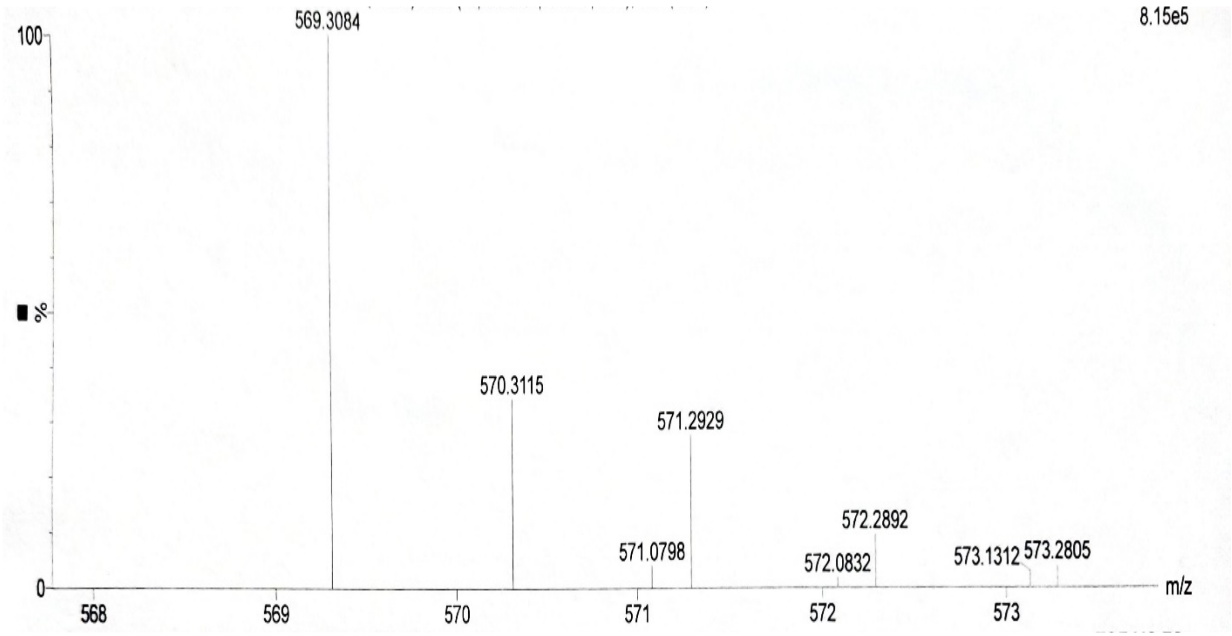

8.15e5
569.3084
570.3115
571.2929
571.0798
572.2892
572.0832
573.1312
573.2805
100
%
0
568
569
570
571
572
573
m/z

#

**Measured Ion Mass(es) :**	569.3084	**Deviation [mmu] :**	0.09 [mmu]
**Calculated Ion Mass(es) :**	569.30849	**Deviation [ppm] :**	0.16 [ppm]
**Potential Molecular Formula :**	C31H46O8Na [illegible]+		

Figure 1: ESI-HR-MS (+) spectrum of compound 1

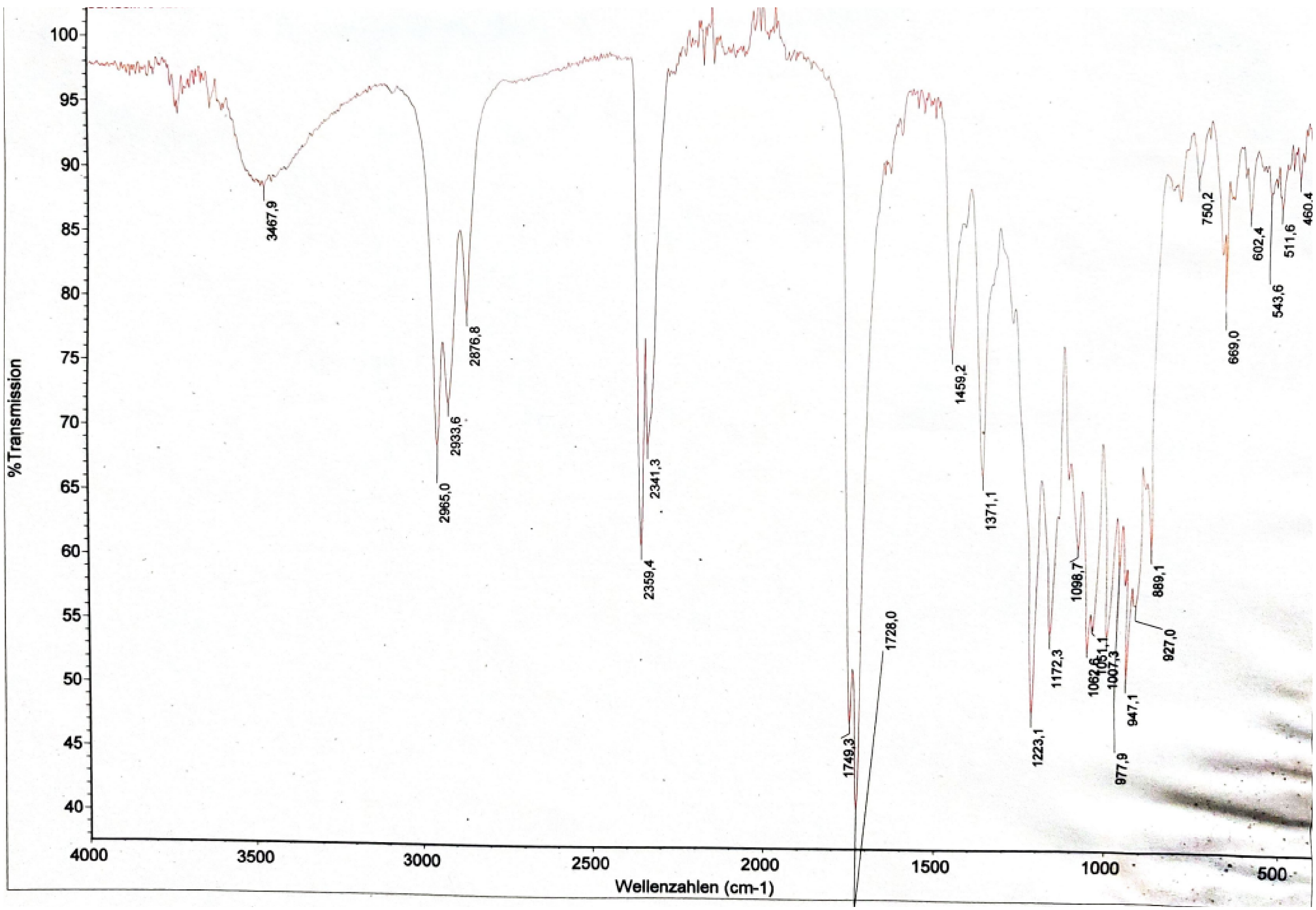

Figure 2: FT-IR spectrum of compound 1

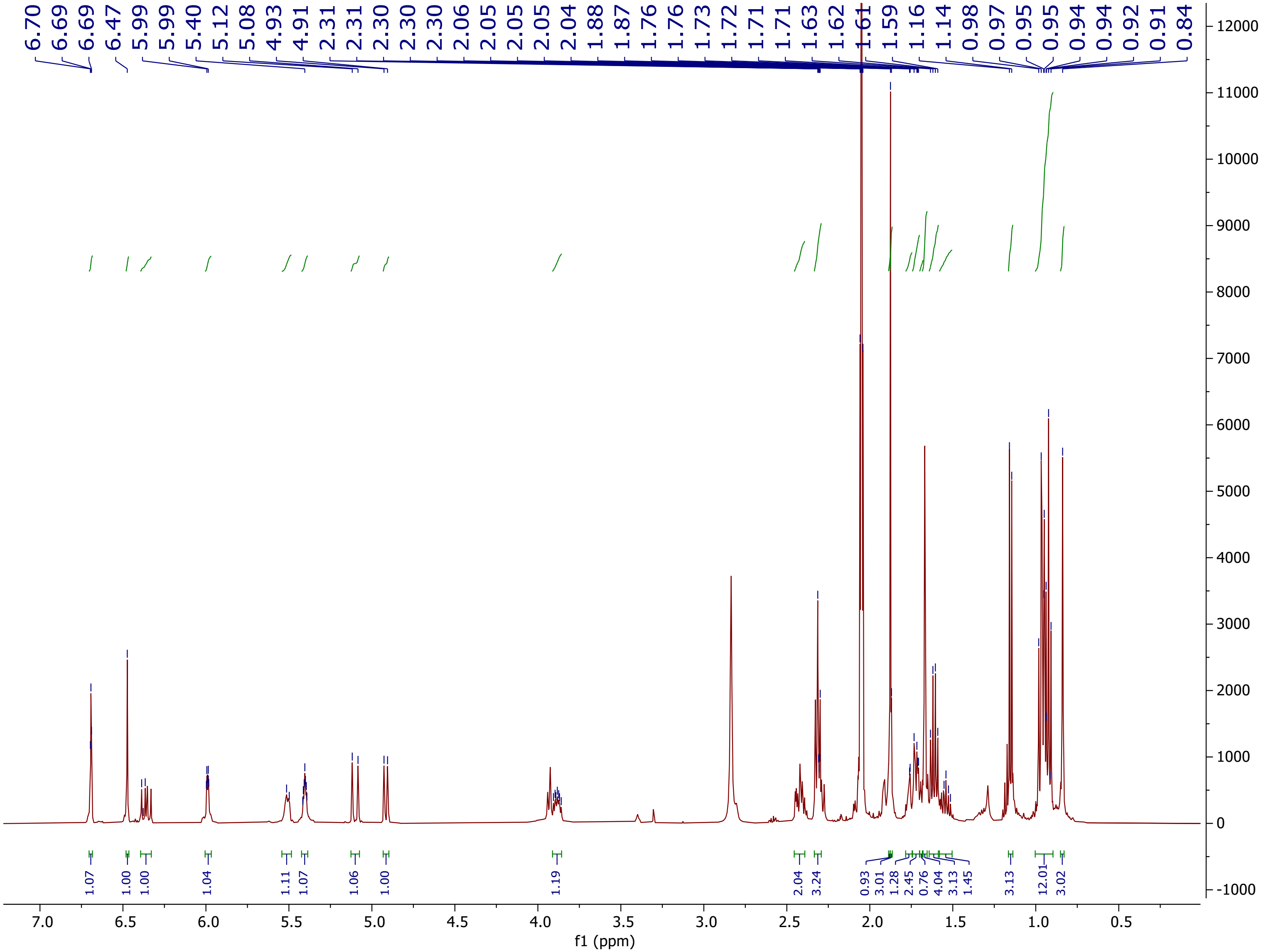

Figure 3: ^{1}H NMR spectrum (500 MHz, acetone-d_6) of compound 1

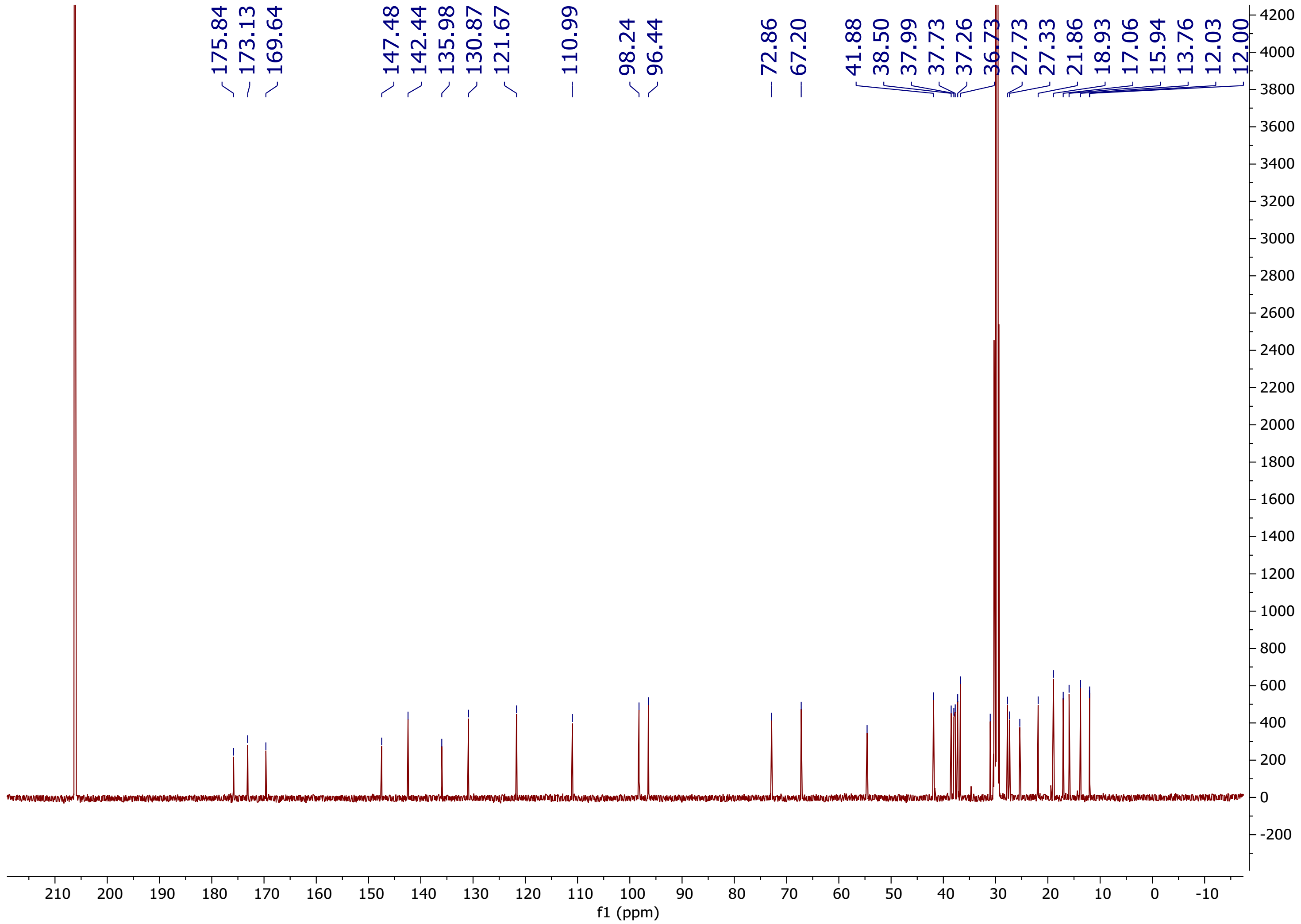

Figure 4: ^{13}C NMR spectrum (125 MHz, acetone-d_6) of compound 1

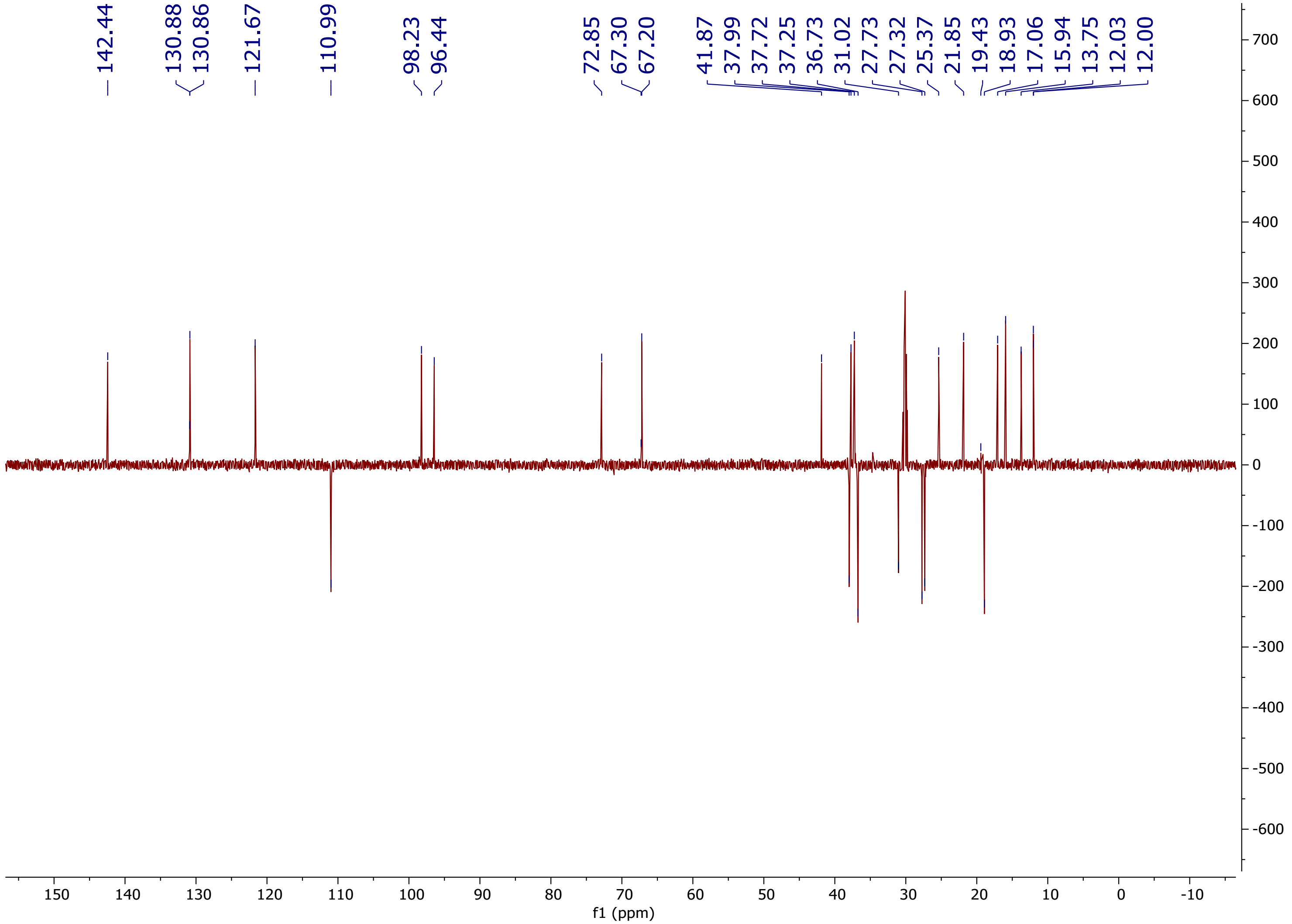

Figure 5: DEPT 135 spectrum (125MHz, acetone-d_6) of compound 1

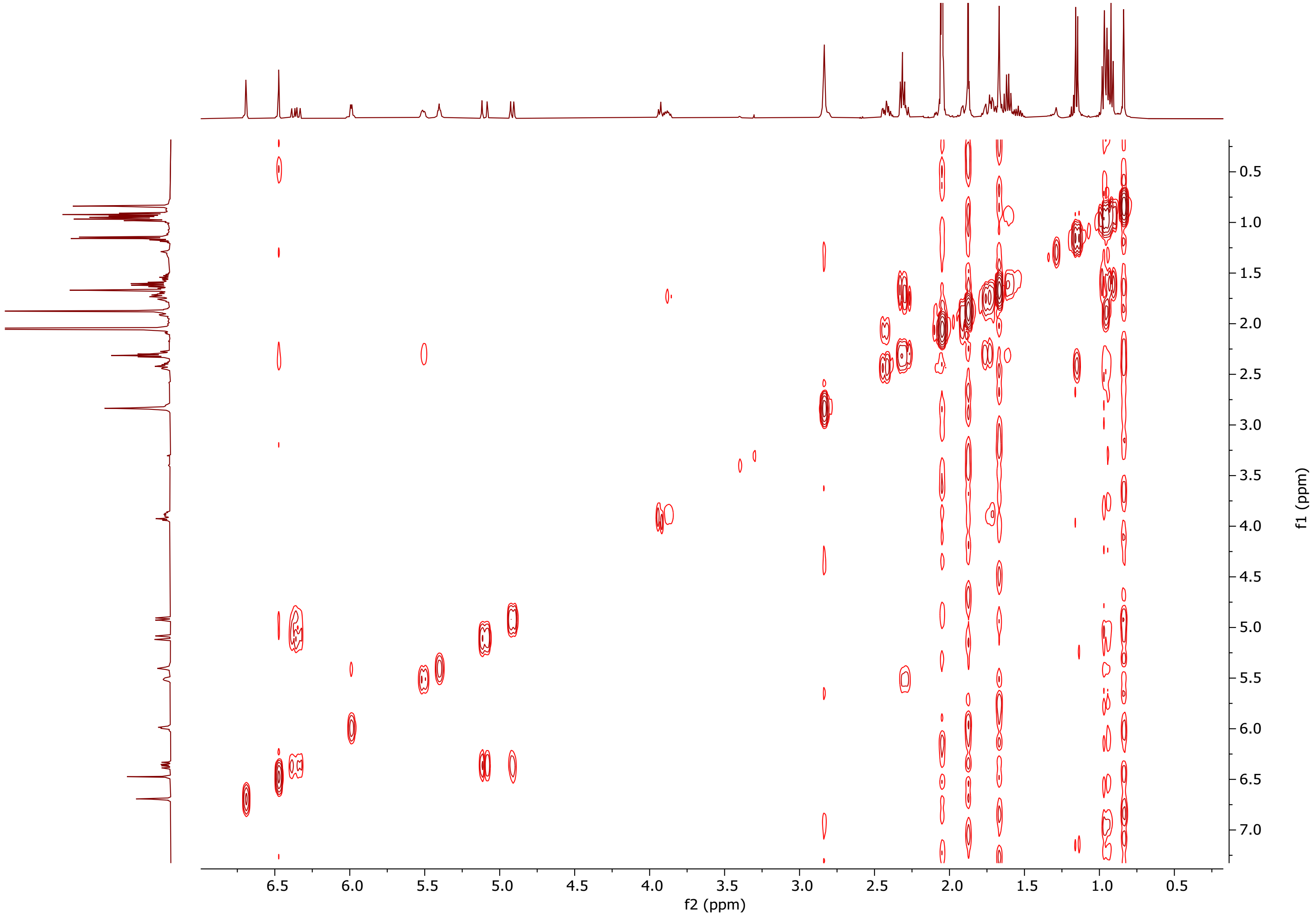

Figure 6: COSY spectrum (500 MHz, acetone-d_6) of compound 1

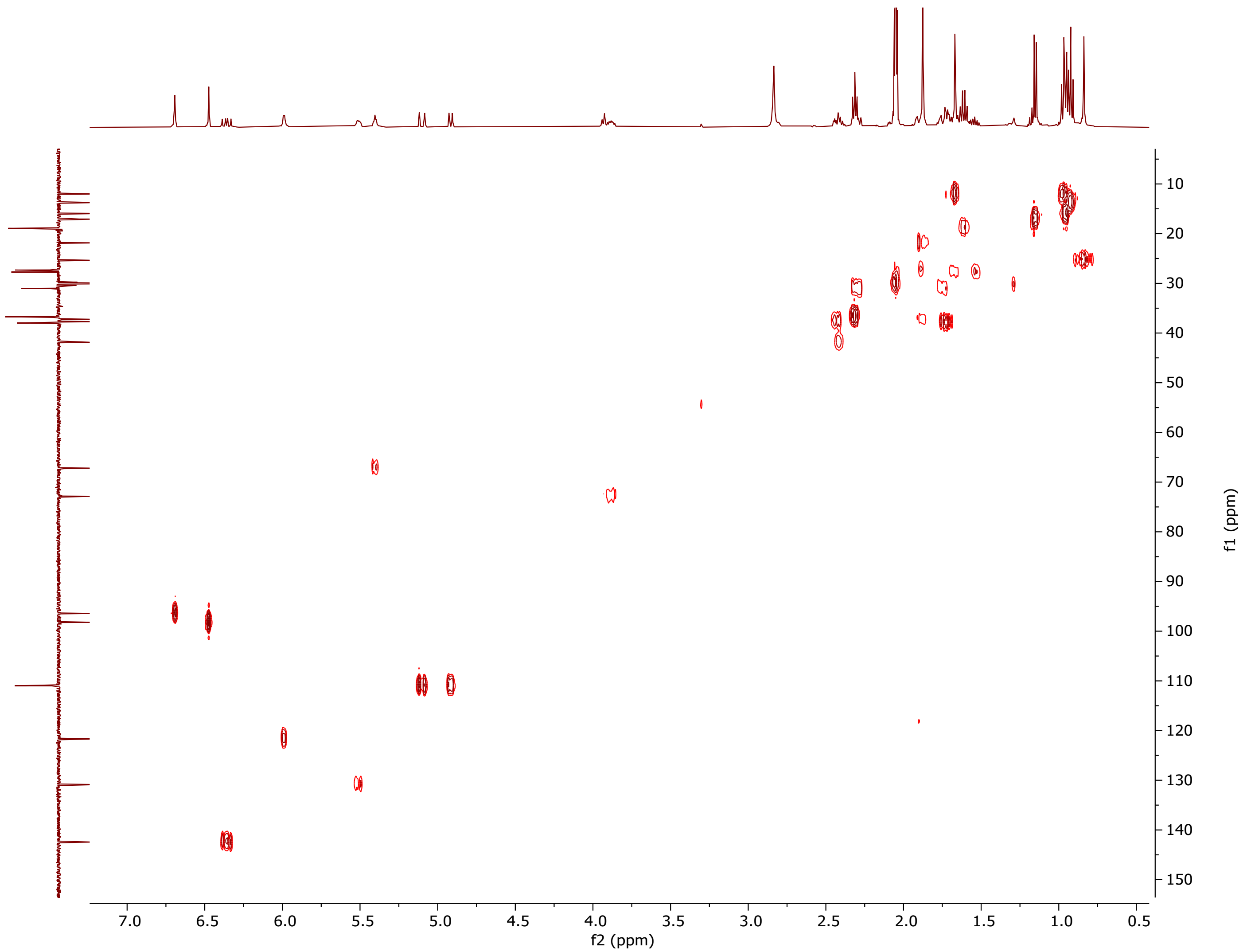

Figure 7: HSQC spectrum (500 MHz, acetone-d_6) of compound 1

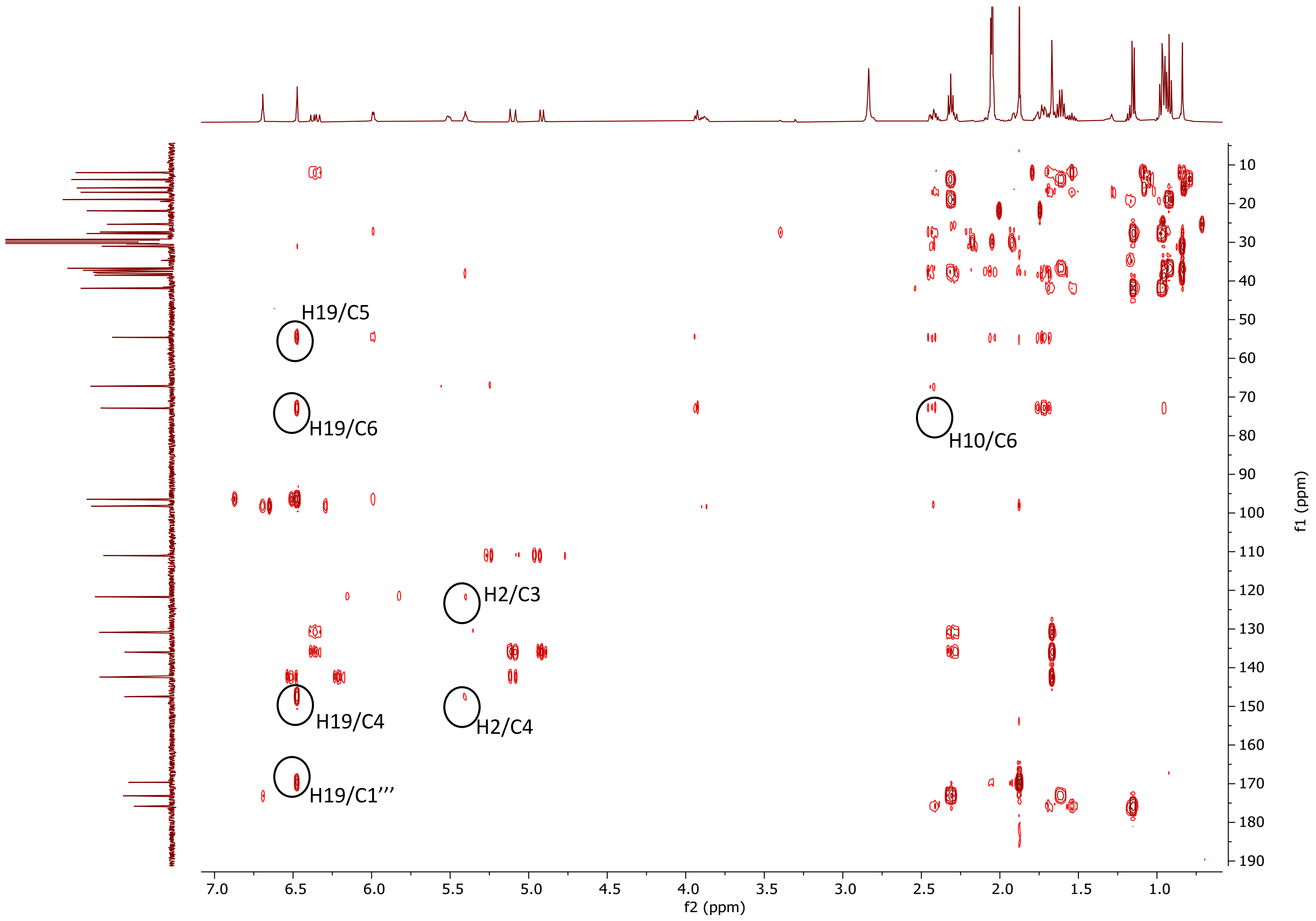

Figure 8: HMBC spectrum (500 MHz, acetone-d_6) of compound 1

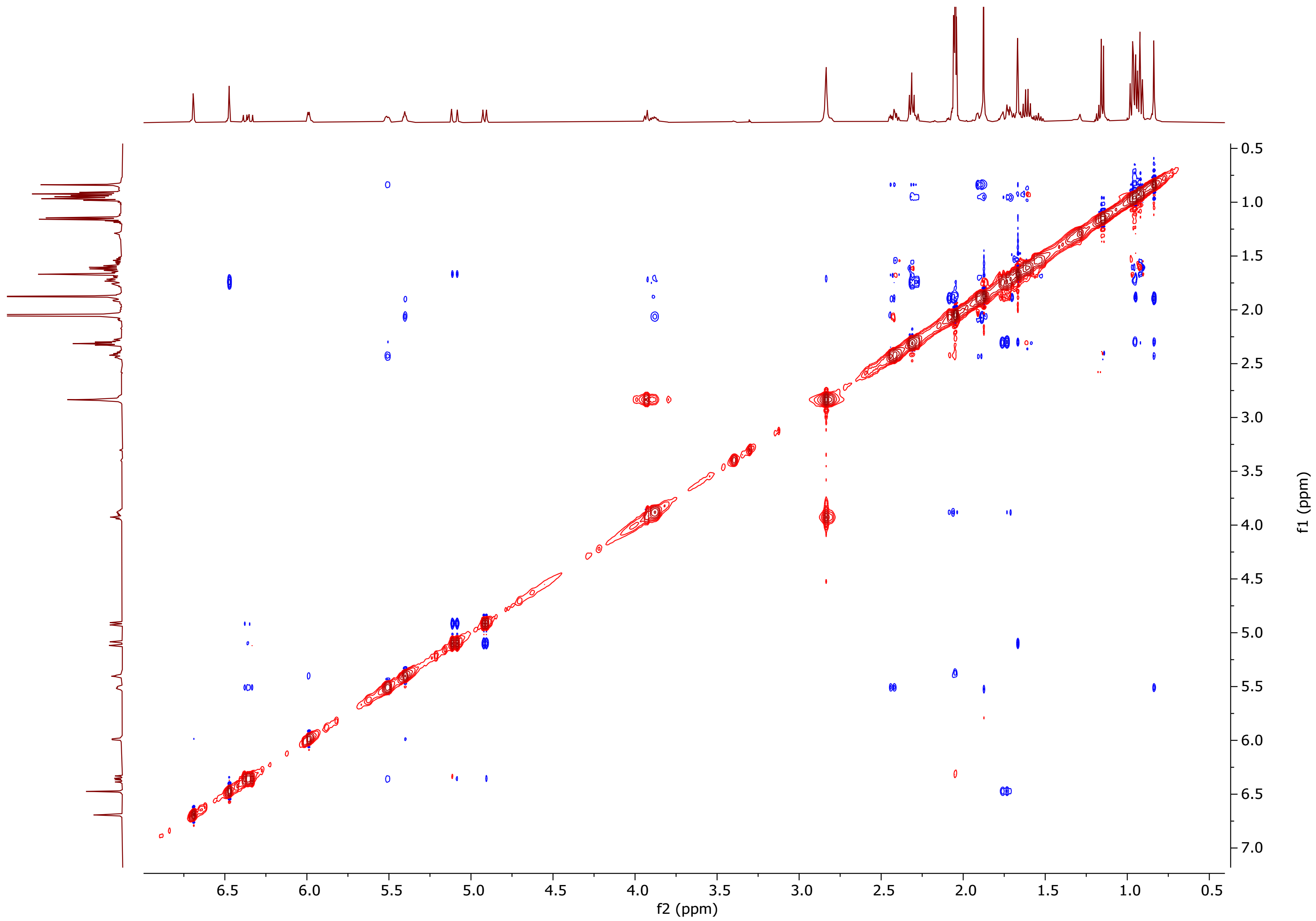

Figure 9: NOESY spectrum (500 MHz, acetone-d_6) of compound 1

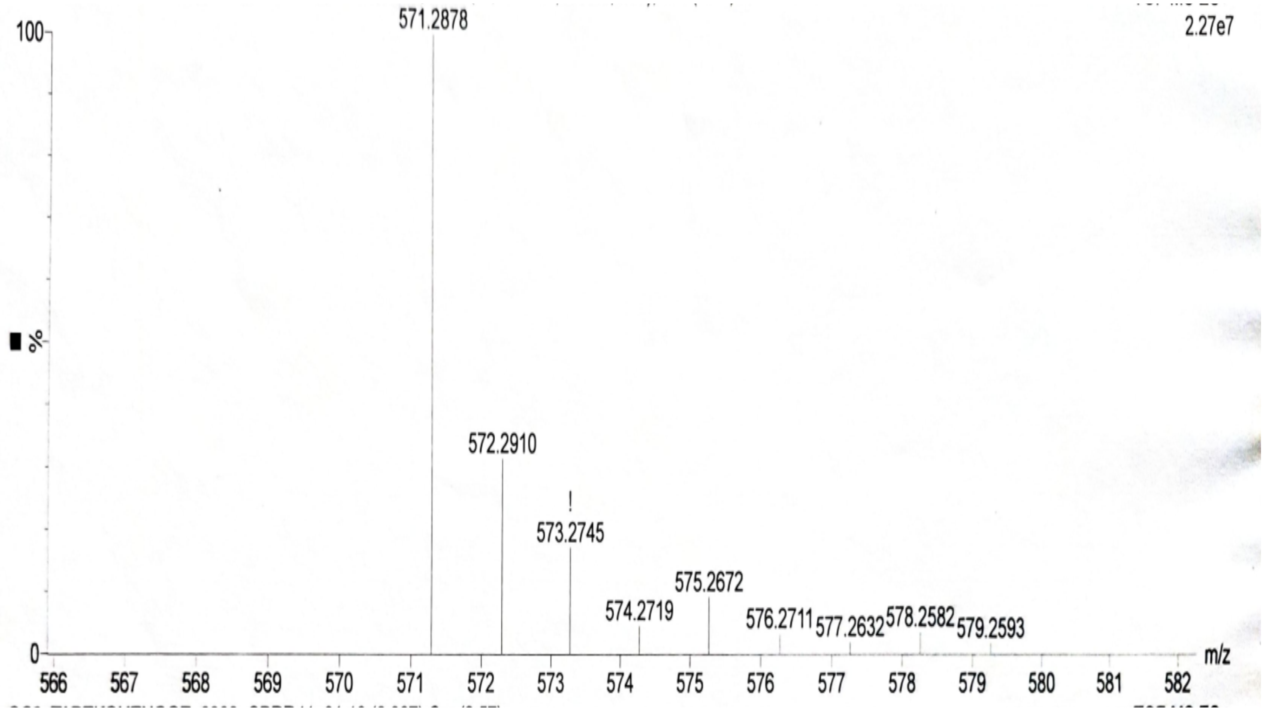

**Measured Ion Mass(es) :**	571.2878	**Deviation [mmu] :**	0.04 [mmu]
**Calculated Ion Mass(es) :**	571.28776	**Deviation [ppm] :**	0.07 [ppm]
**Potential Molecular Formula :**	$C_{30}H_{44}O_9Na^+$		

Figure 10: ESI-HR-MS (+) spectrum of compound 2

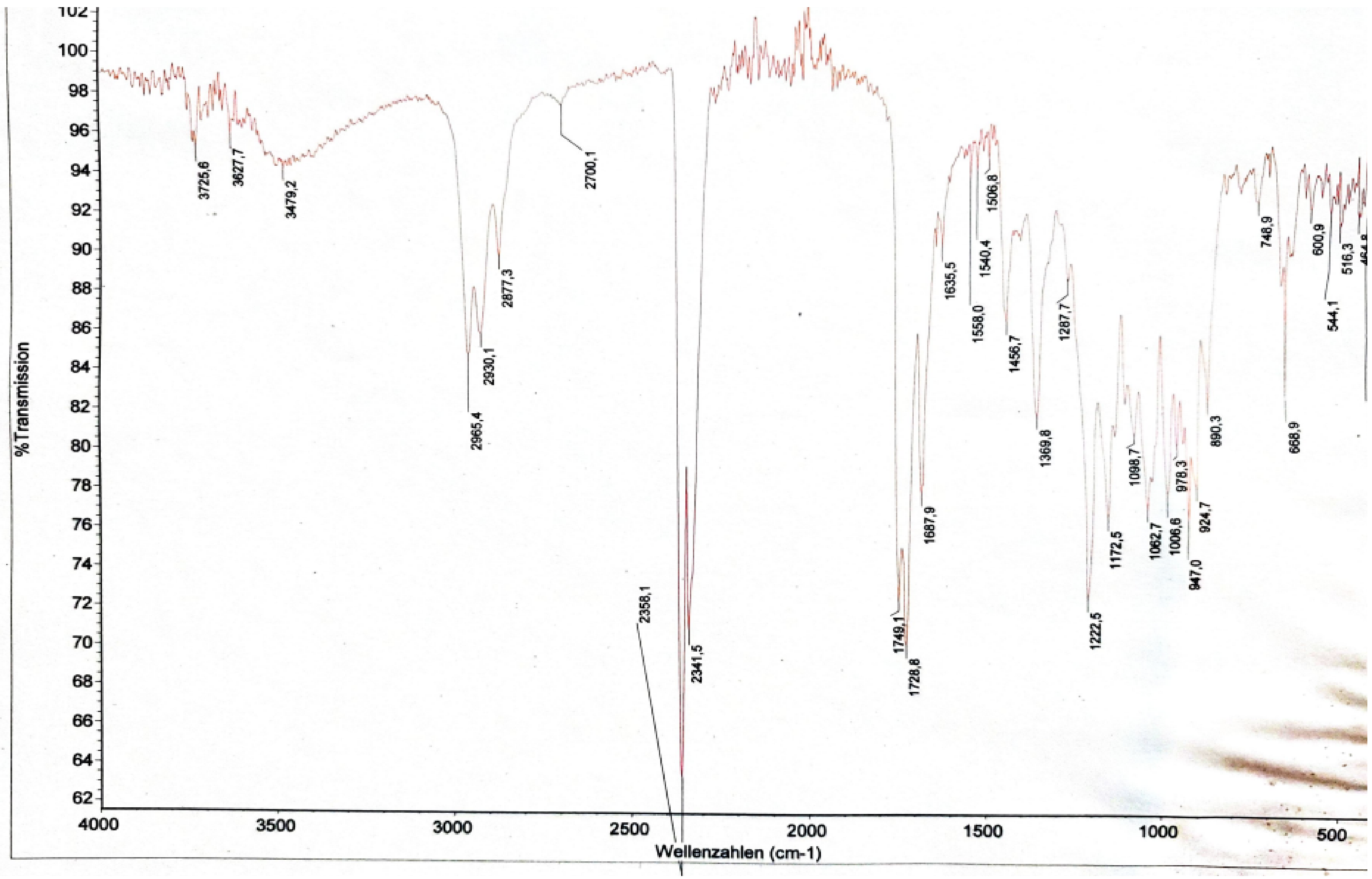

Figure 11: FT-IR spectrum of compound 2

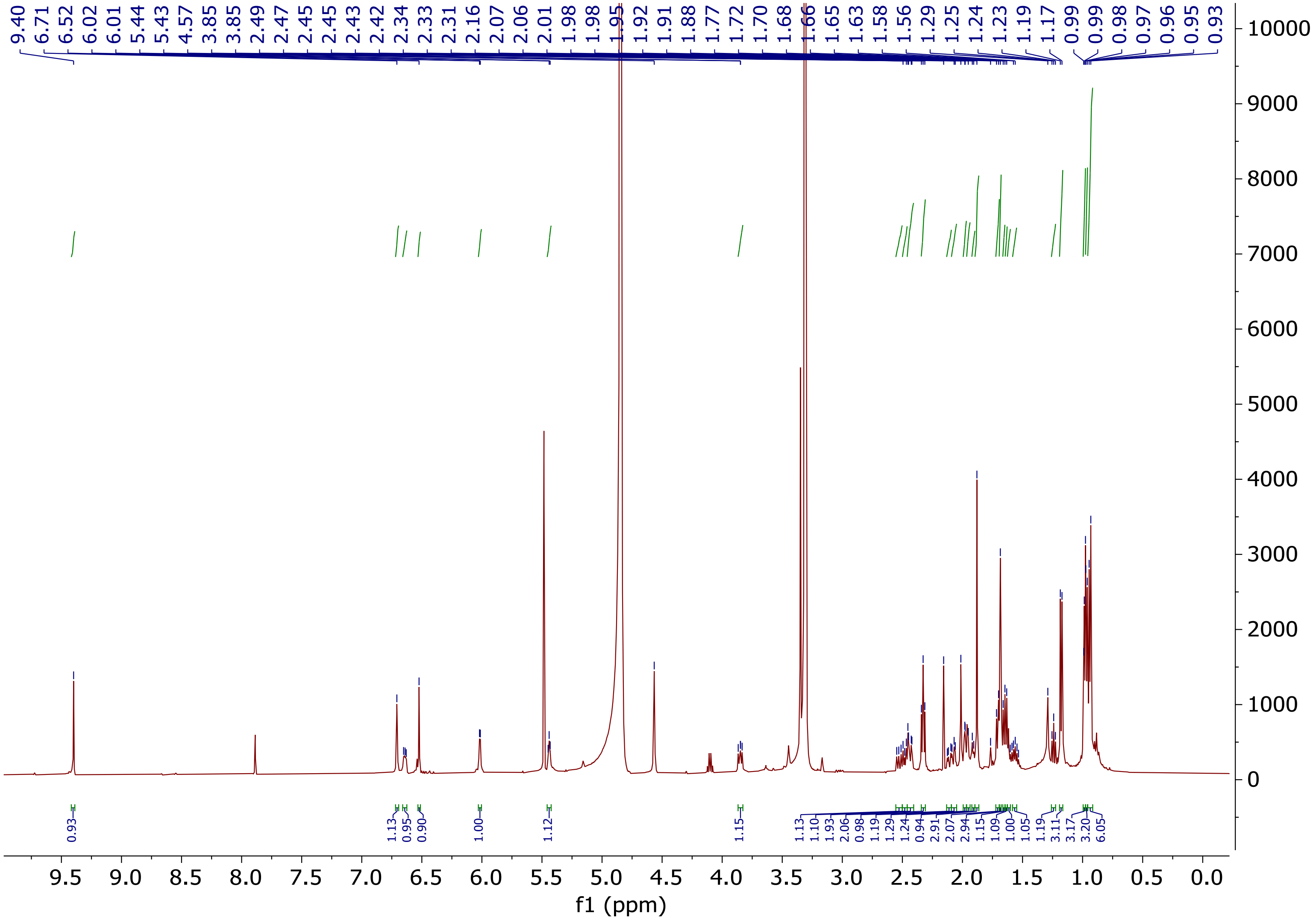

Figure 12: ^{1}H NMR spectrum (500 MHz, methanol-d_4) of compound 2

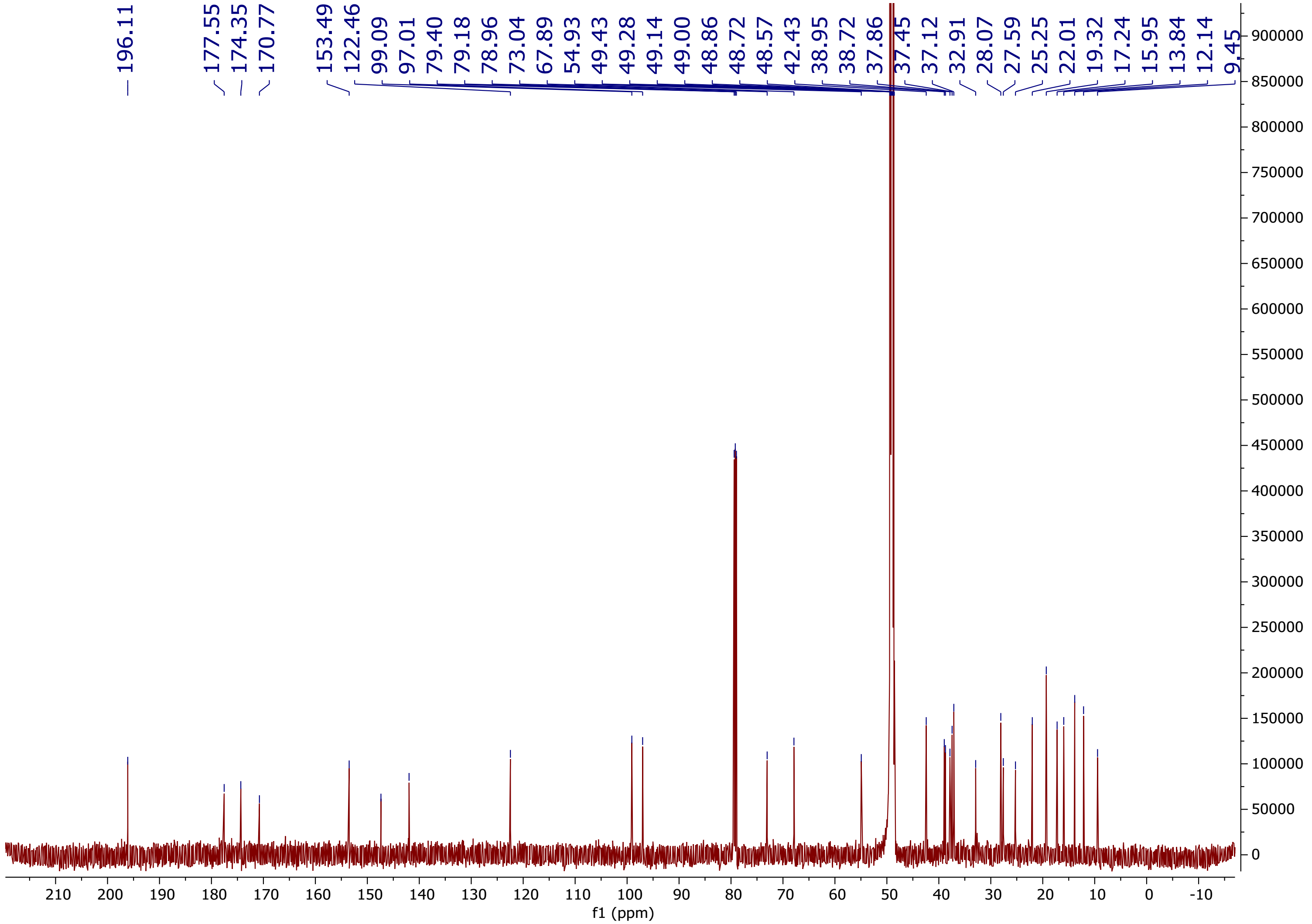

Figure 13: ^{13}C NMR spectrum (125 MHz, methanol-d_4) of compound 2

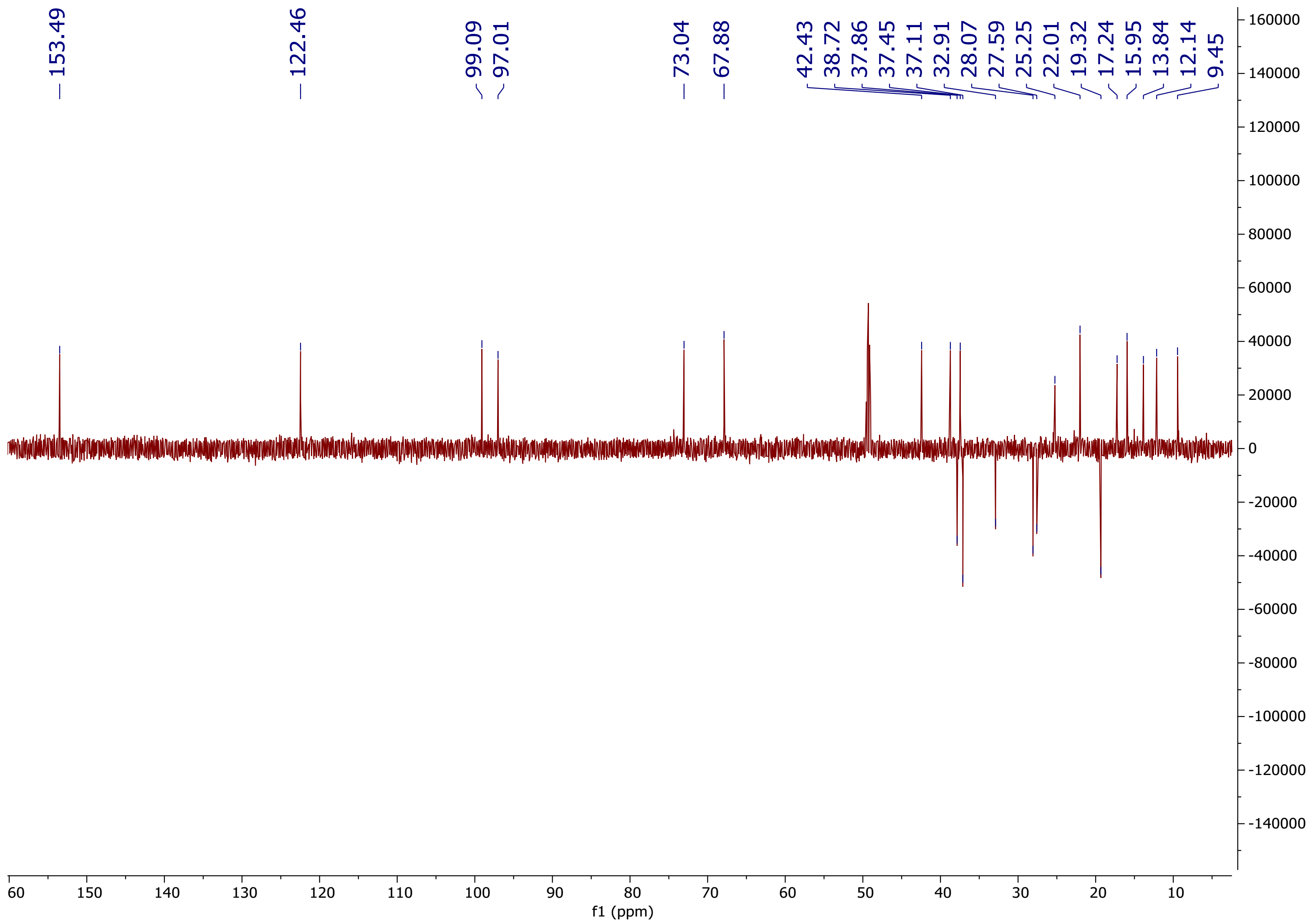

Figure 14: DEPT 135 spectrum (125MHz, methanol-d_4) of compound 2

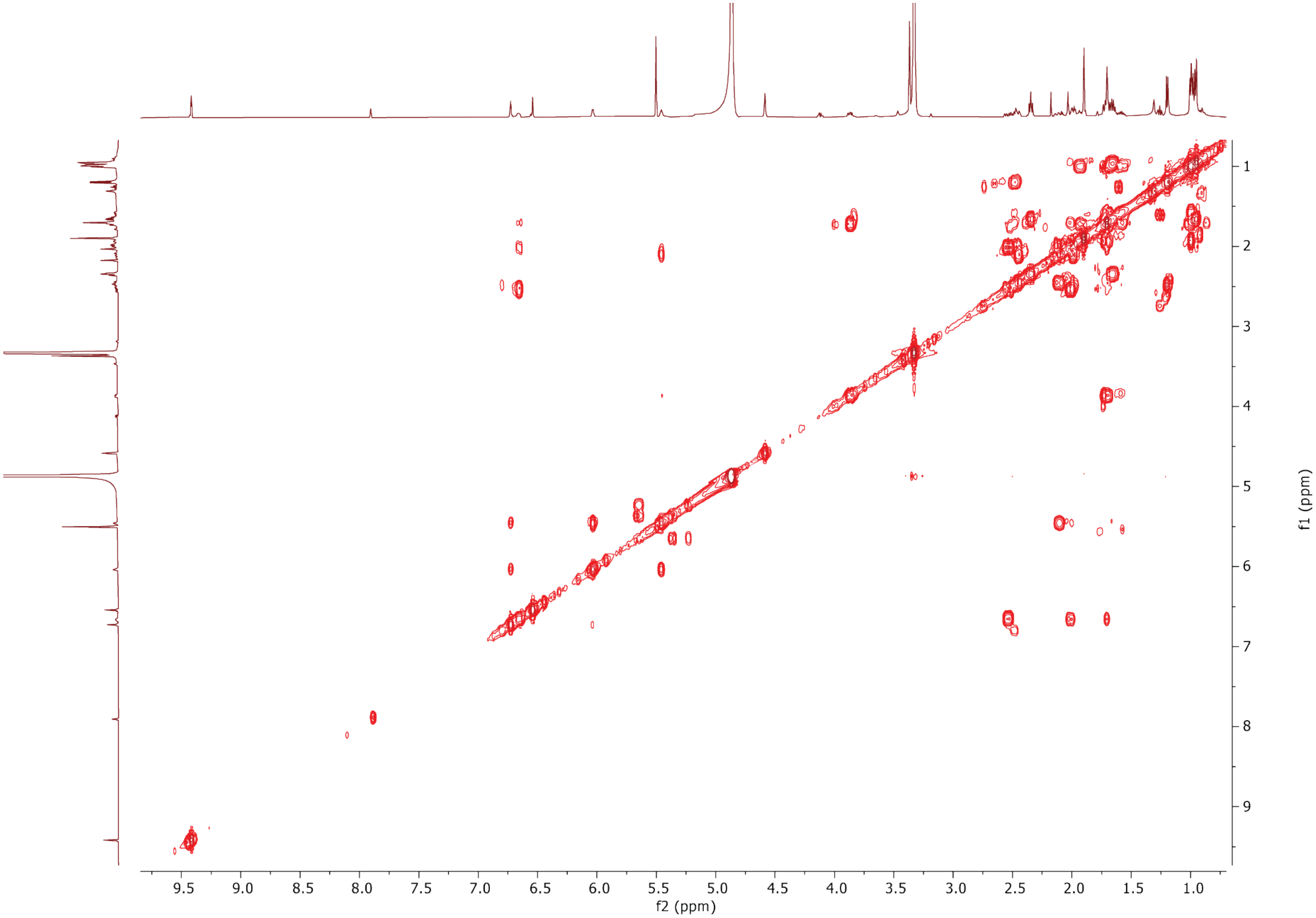

Figure 15: COSY spectrum (500 MHz, methanol-d_4) of compound 2

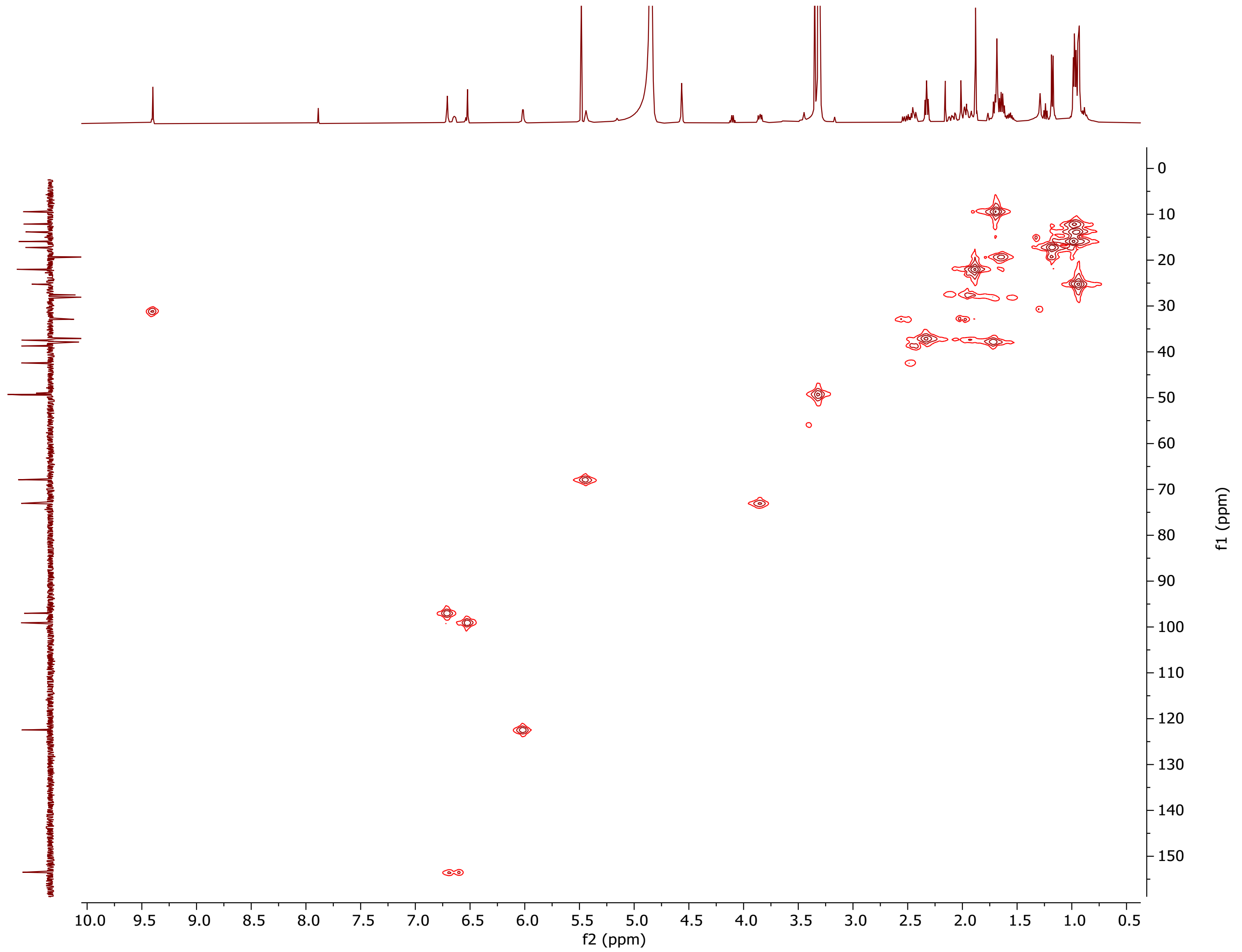

Figure 16: HSQC spectrum (500 MHz, methanol-d_4) of compound 2

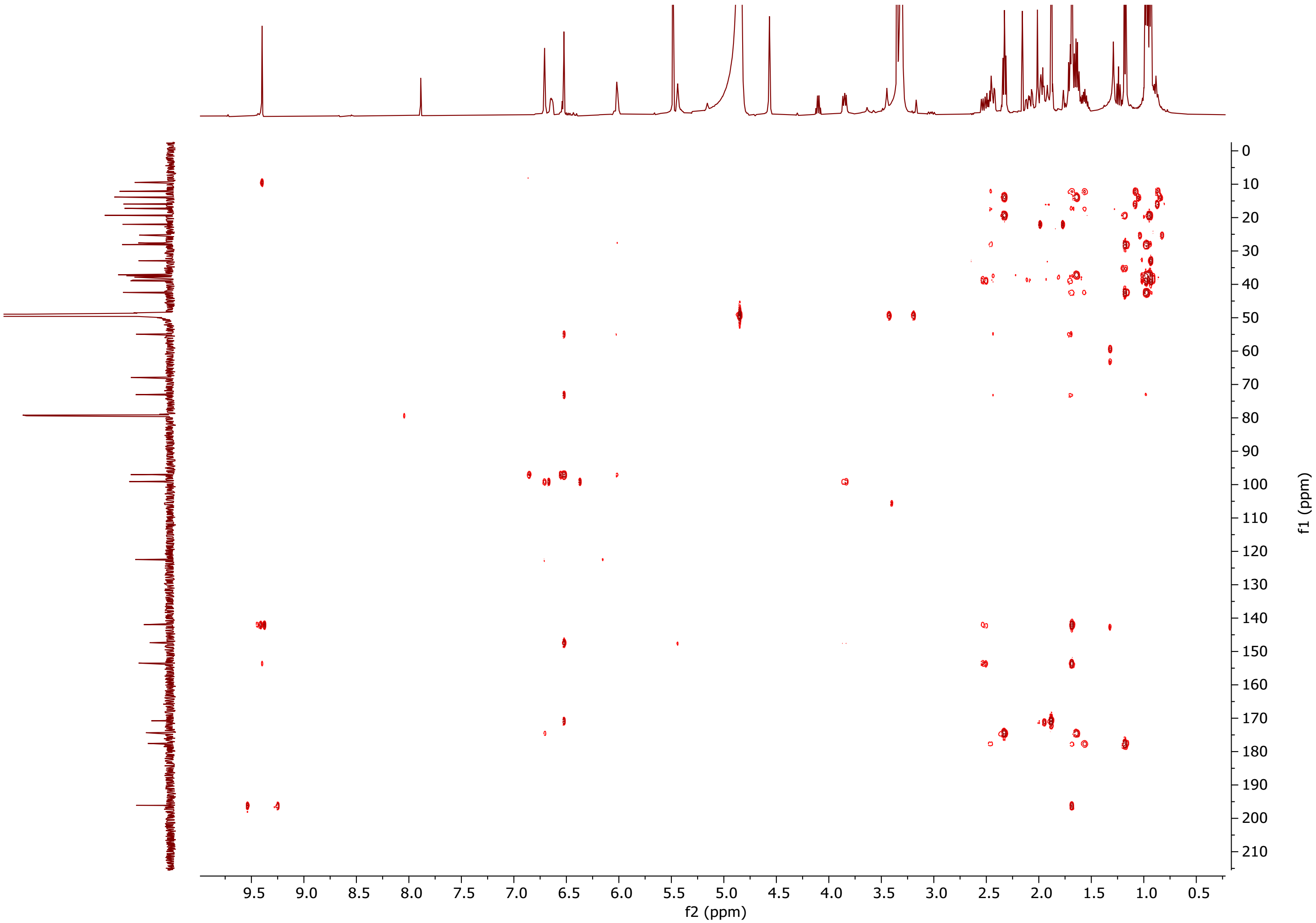

Figure 17: HMBC spectrum (500 MHz, methanol-d_4) of compound 2

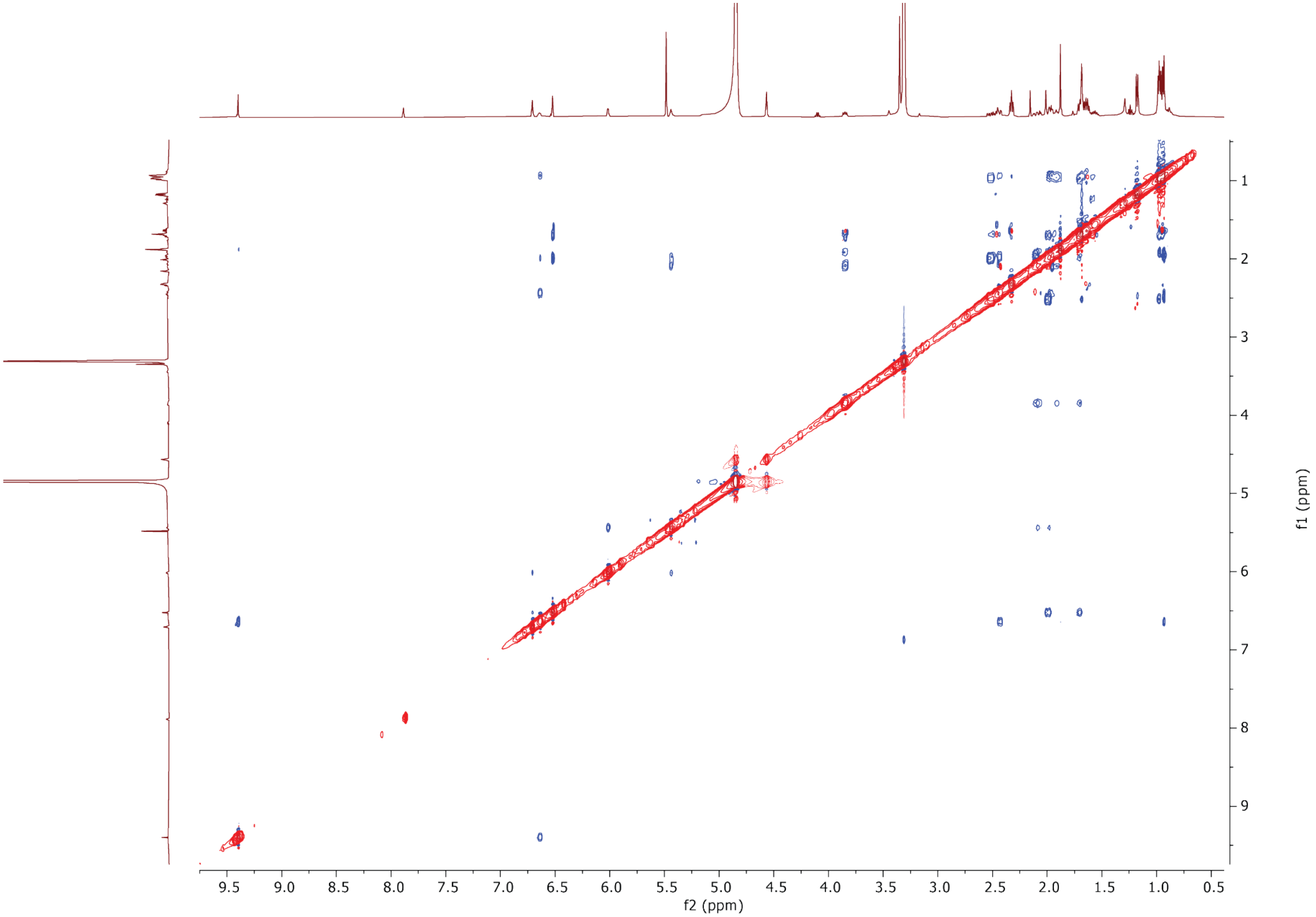

Figure 18: NOESY spectrum (500 MHz, methanol-d_4) of compound 2

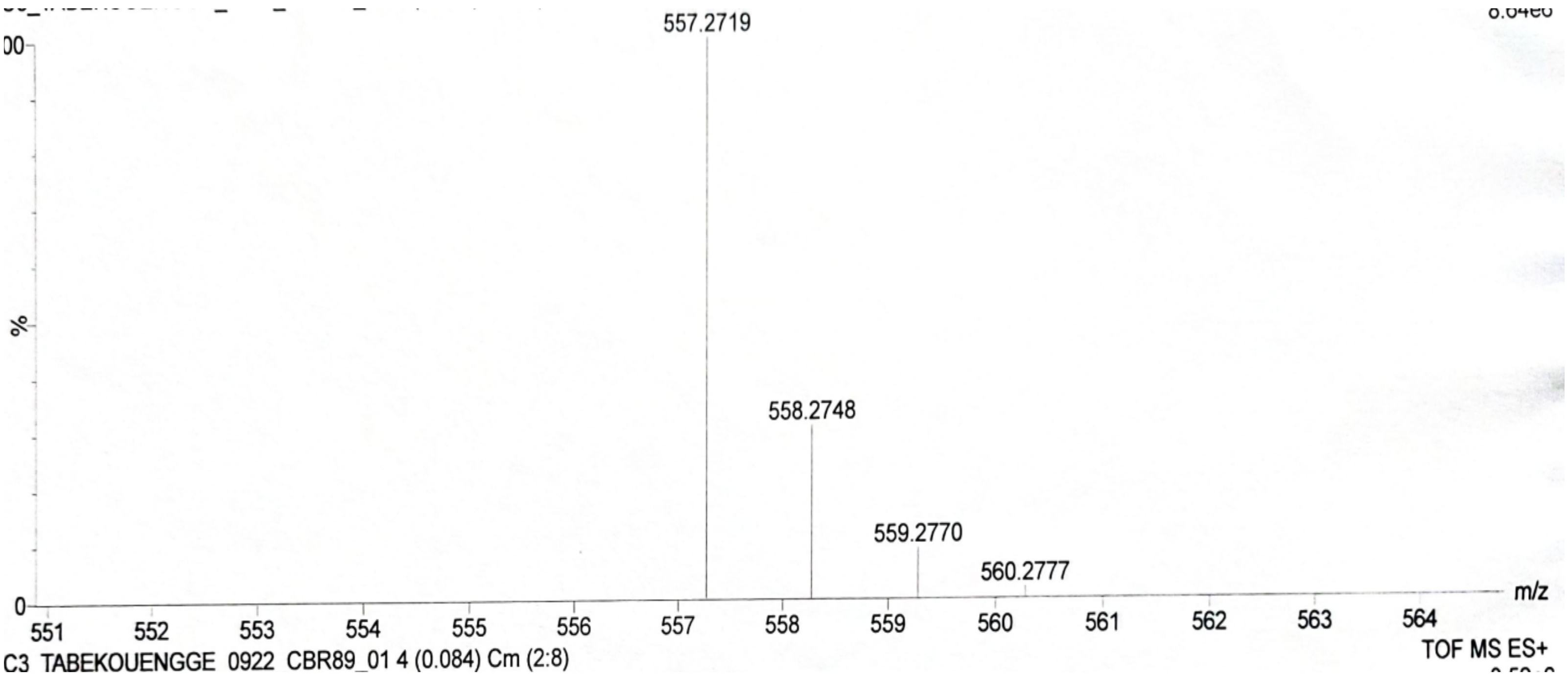

Measured Ion Mass(es) : 557.2719 **Deviation [mmu] :** 0.21 [mmu]

Calculated Ion Mass(es) : 557.27211 **Deviation [ppm] :** 0.38 [ppm]

Potential Molecular Formula : C29H42O9Na+

Figure 19: ESI-HR-MS (+) spectrum of compound 3

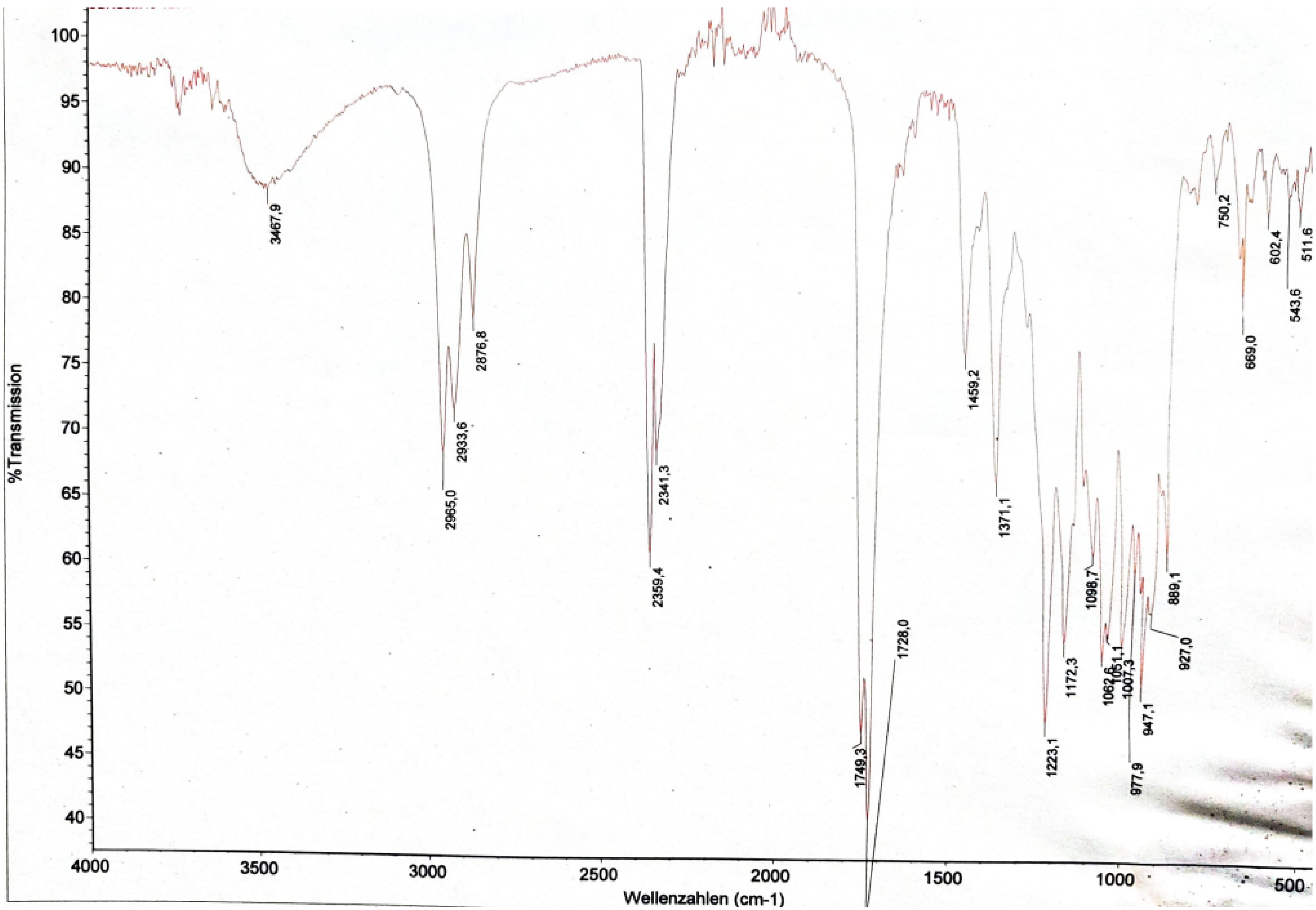

Figure 20: FT-IR spectrum of compound 3

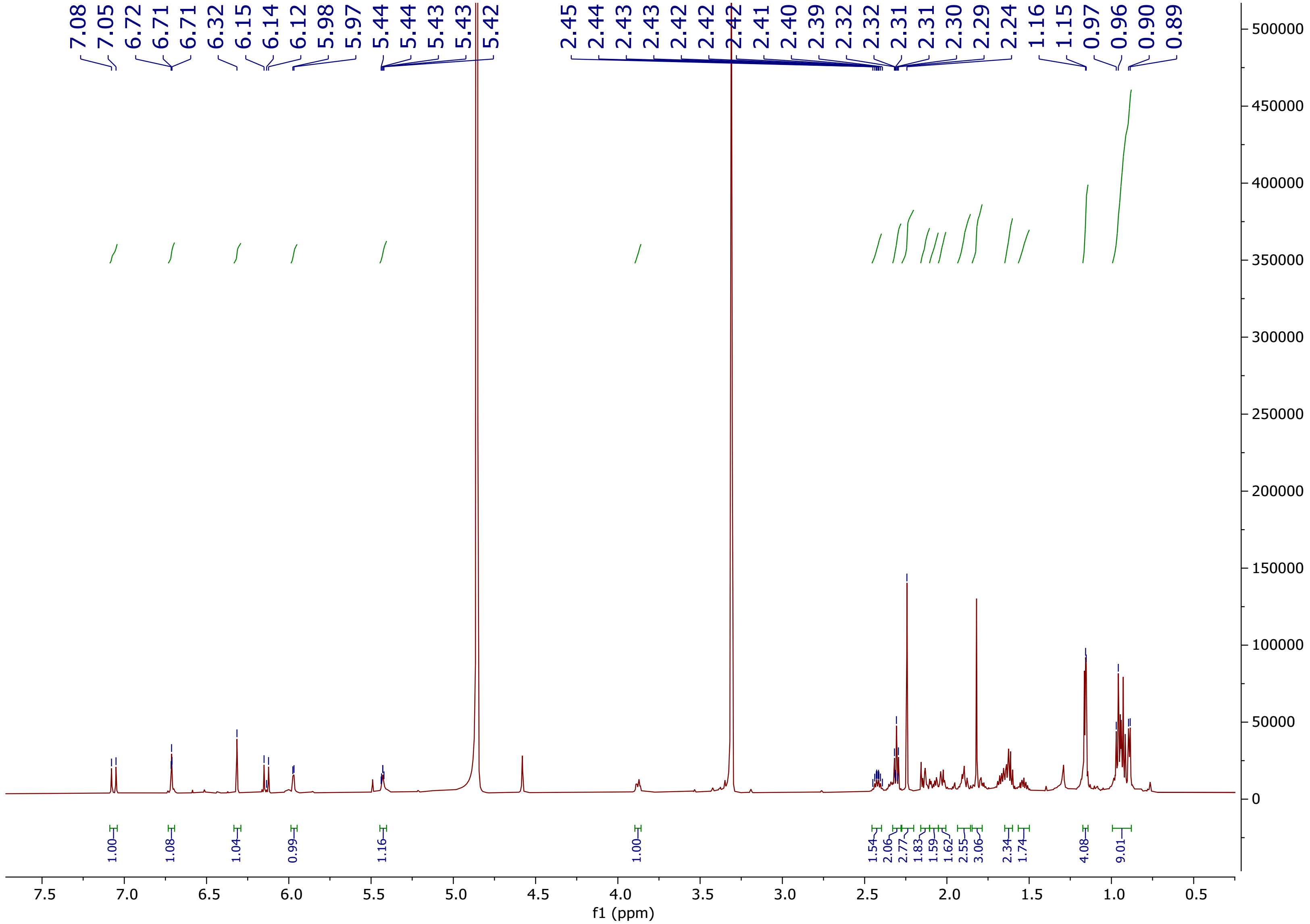

Figure 21: ^{1}H NMR spectrum (500 MHz, methanol-d_4) of compound 3

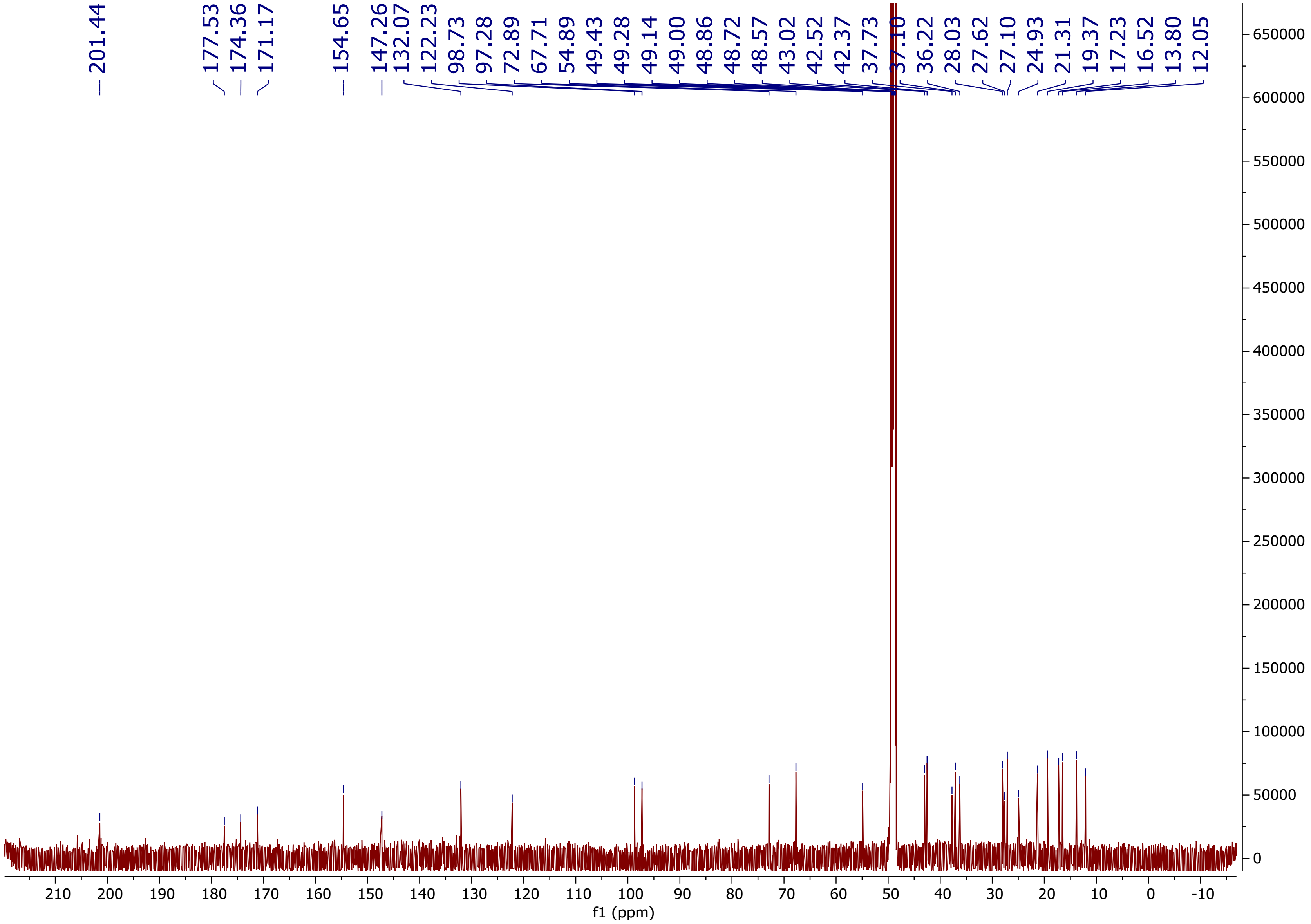

Figure 22: ^{13}C NMR spectrum (125 MHz, methanol-d_4) of compound 3

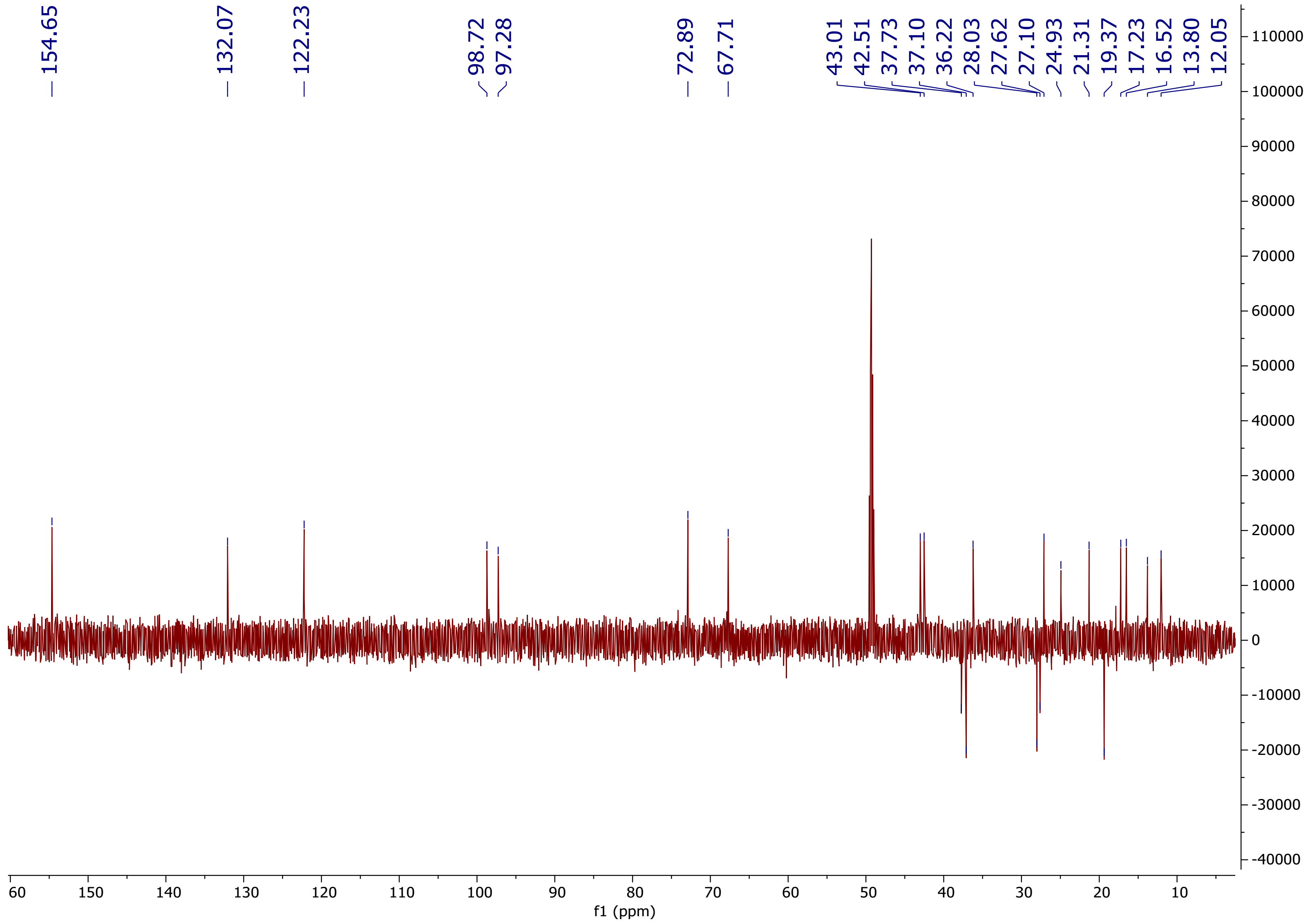

Figure 23: DEPT 135 spectrum (125MHz, methanol-d_4) of compound 3

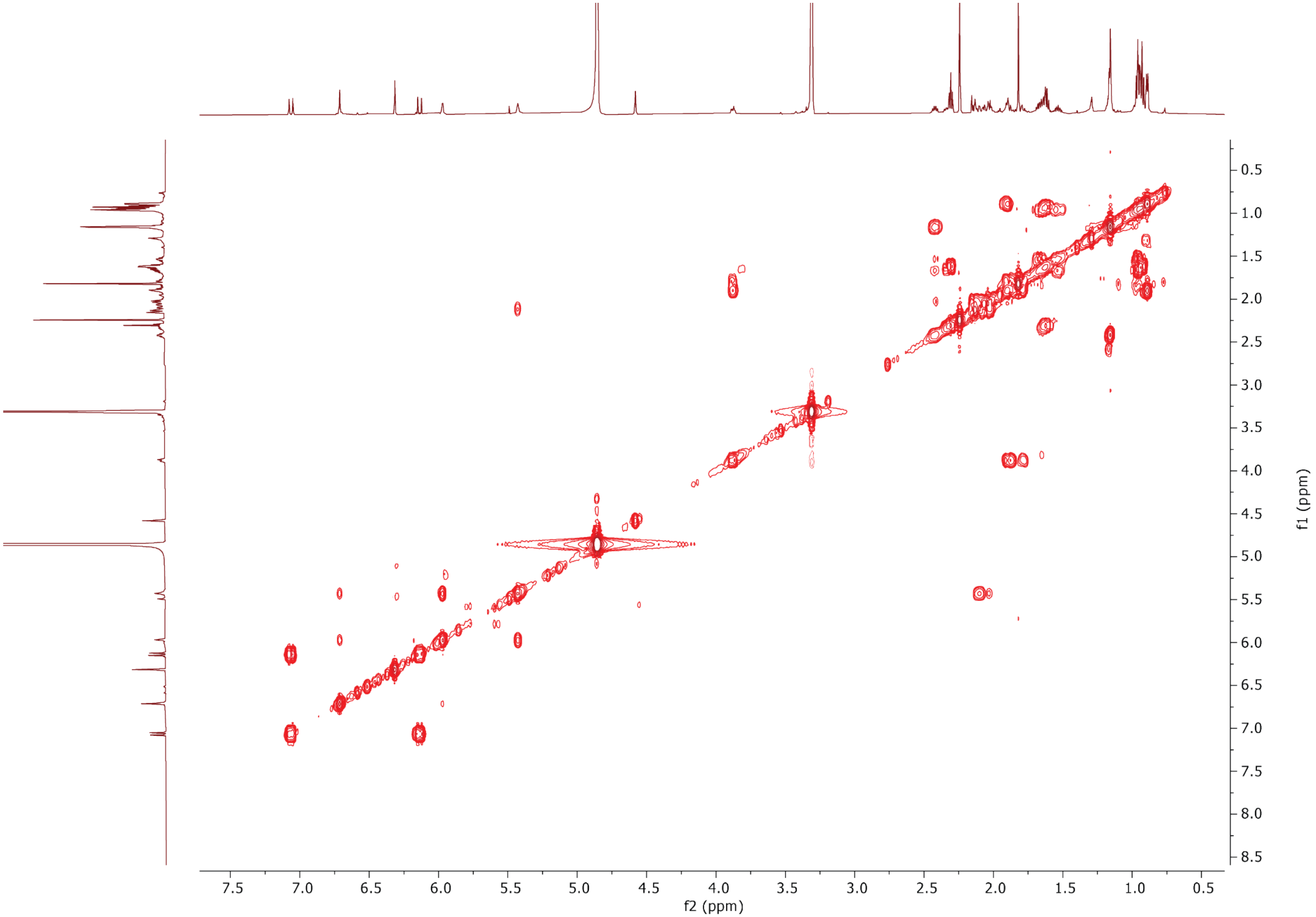

Figure 24: COSY spectrum (500 MHz, methanol-d_4) of compound 3

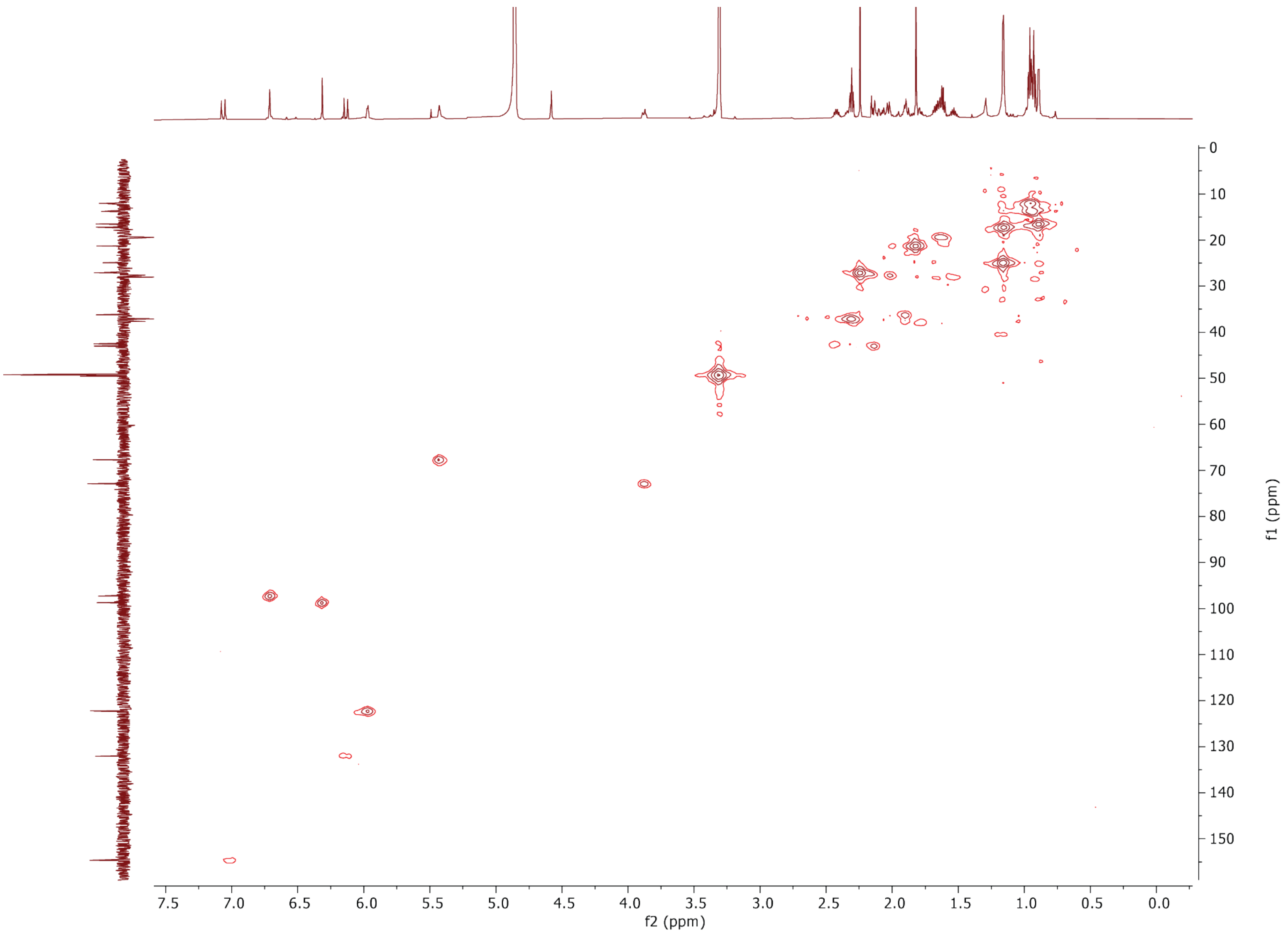

Figure 25: HSQC spectrum (500 MHz, methanol-d_4) of compound 3

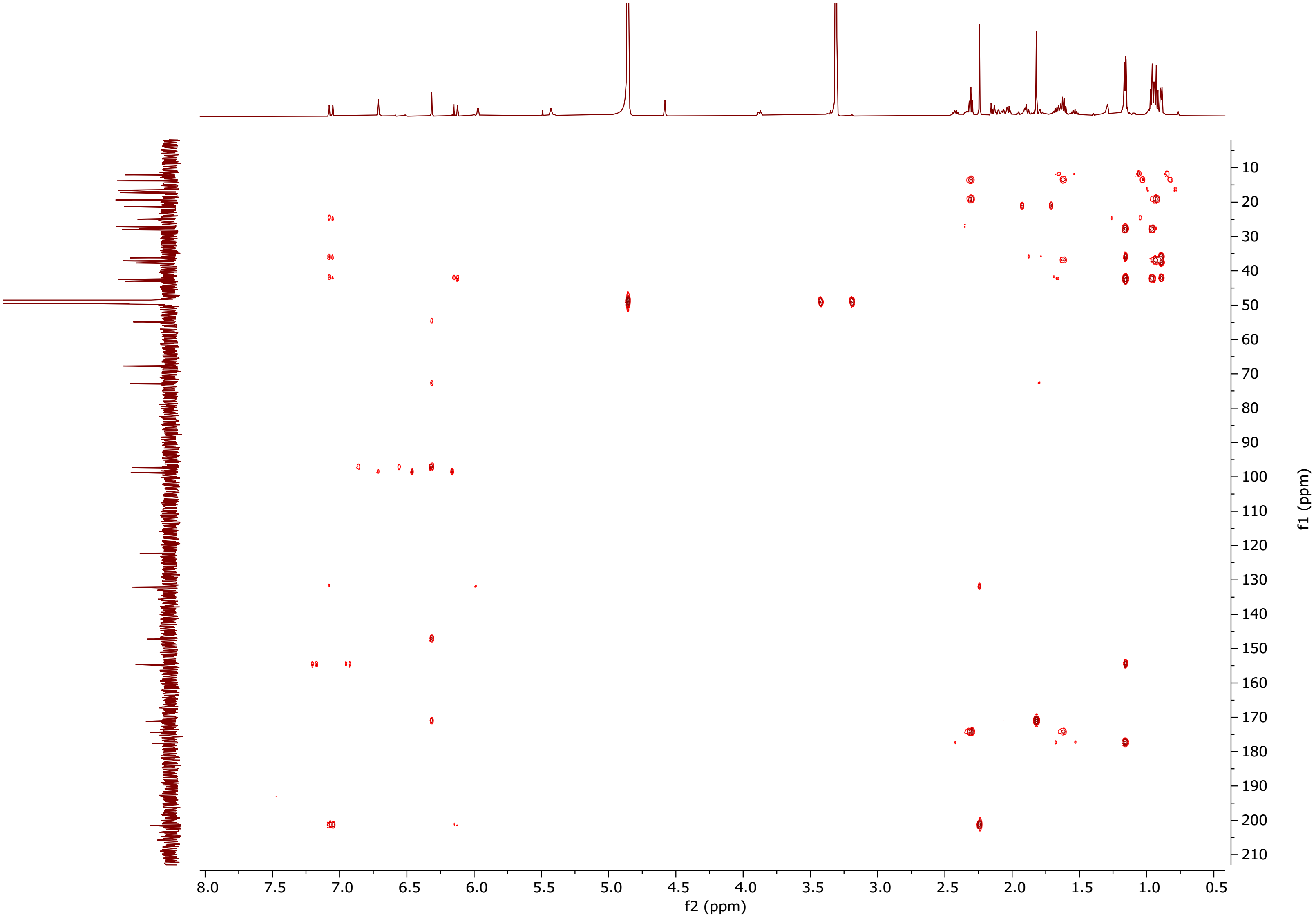

Figure 26: HMBC spectrum (500 MHz, methanol-d_4) of compound 3

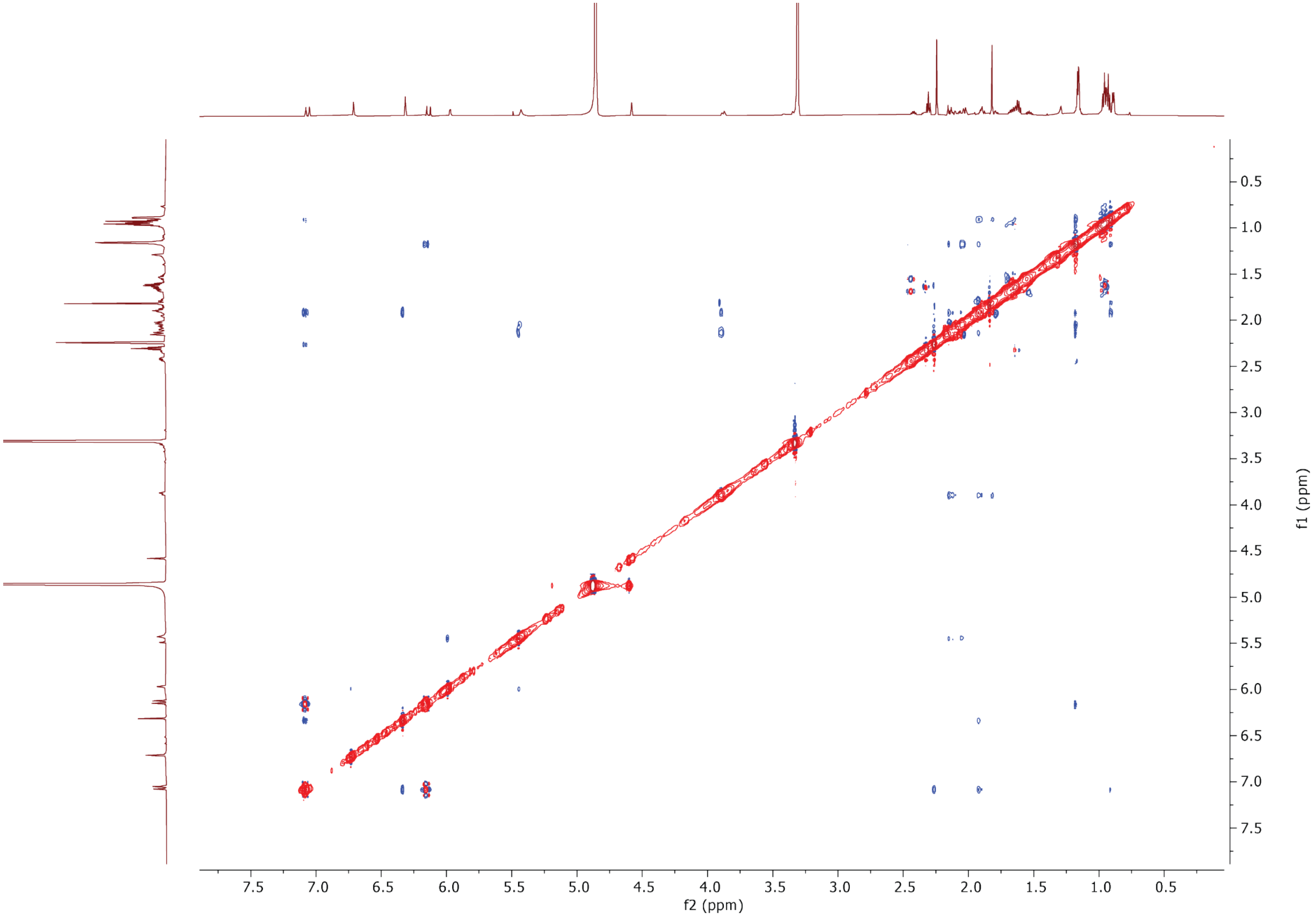

Figure 27: NOESY spectrum (500 MHz, methanol-d_4) of compound 3

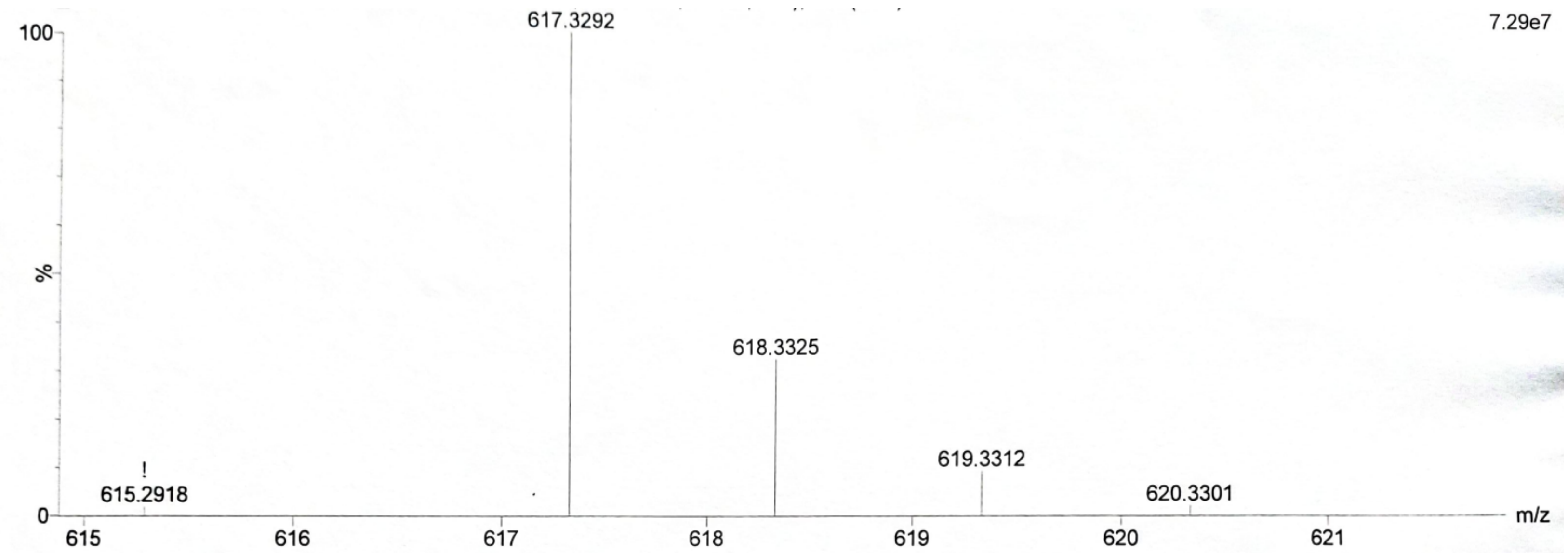

**Measured Ion Mass(es) :**	617.3292	**Deviation [mmu] :**	0.42 [mmu]
**Calculated Ion Mass(es) :**	617.32962	**Deviation [ppm] :**	0.68 [ppm]
**Potential Molecular Formula :**	C32H50O10Na [illegible]+		

Figure 28: ESI-HR-MS (+) spectrum of compound 4

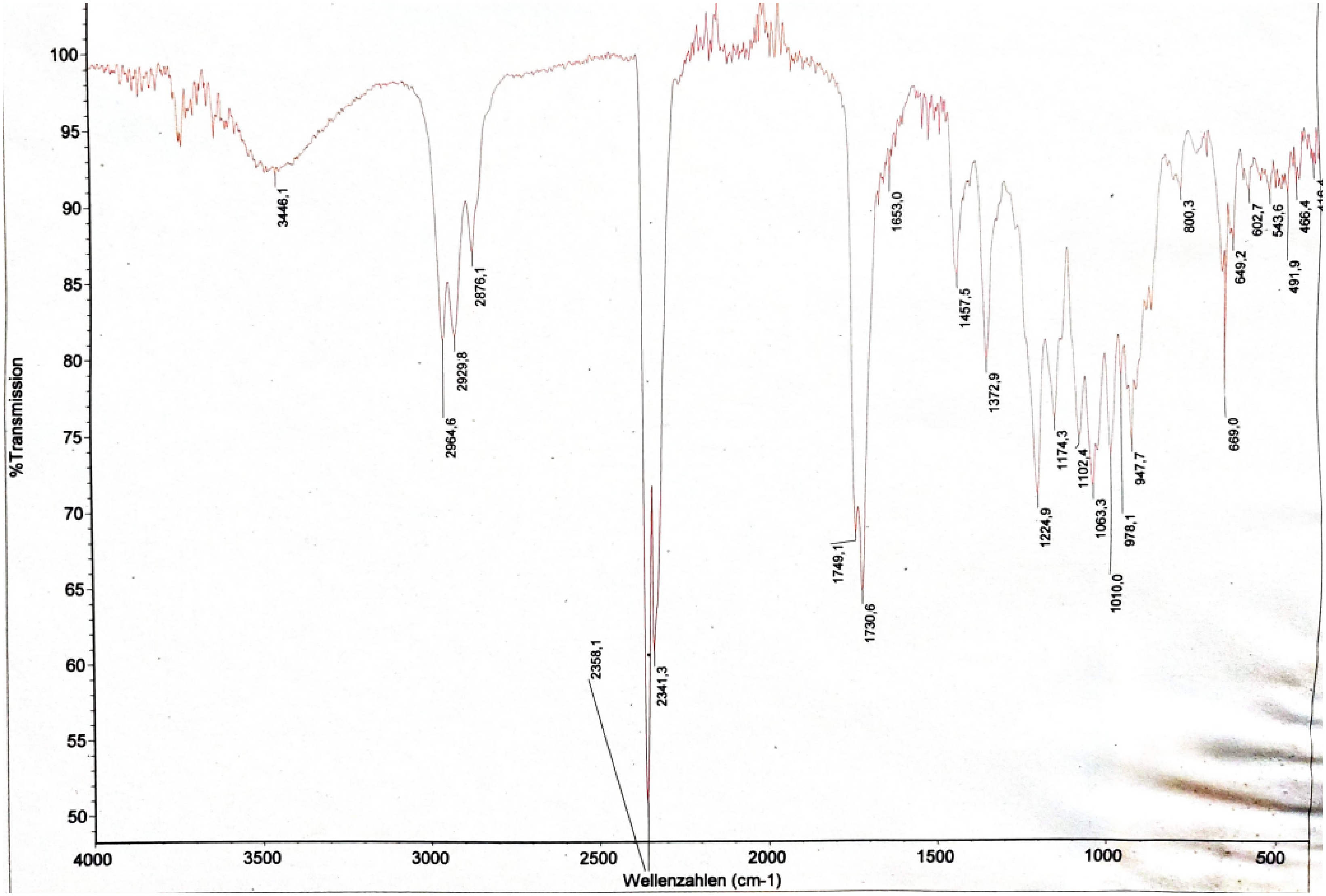

Figure 29: FT-IR spectrum of compound 4

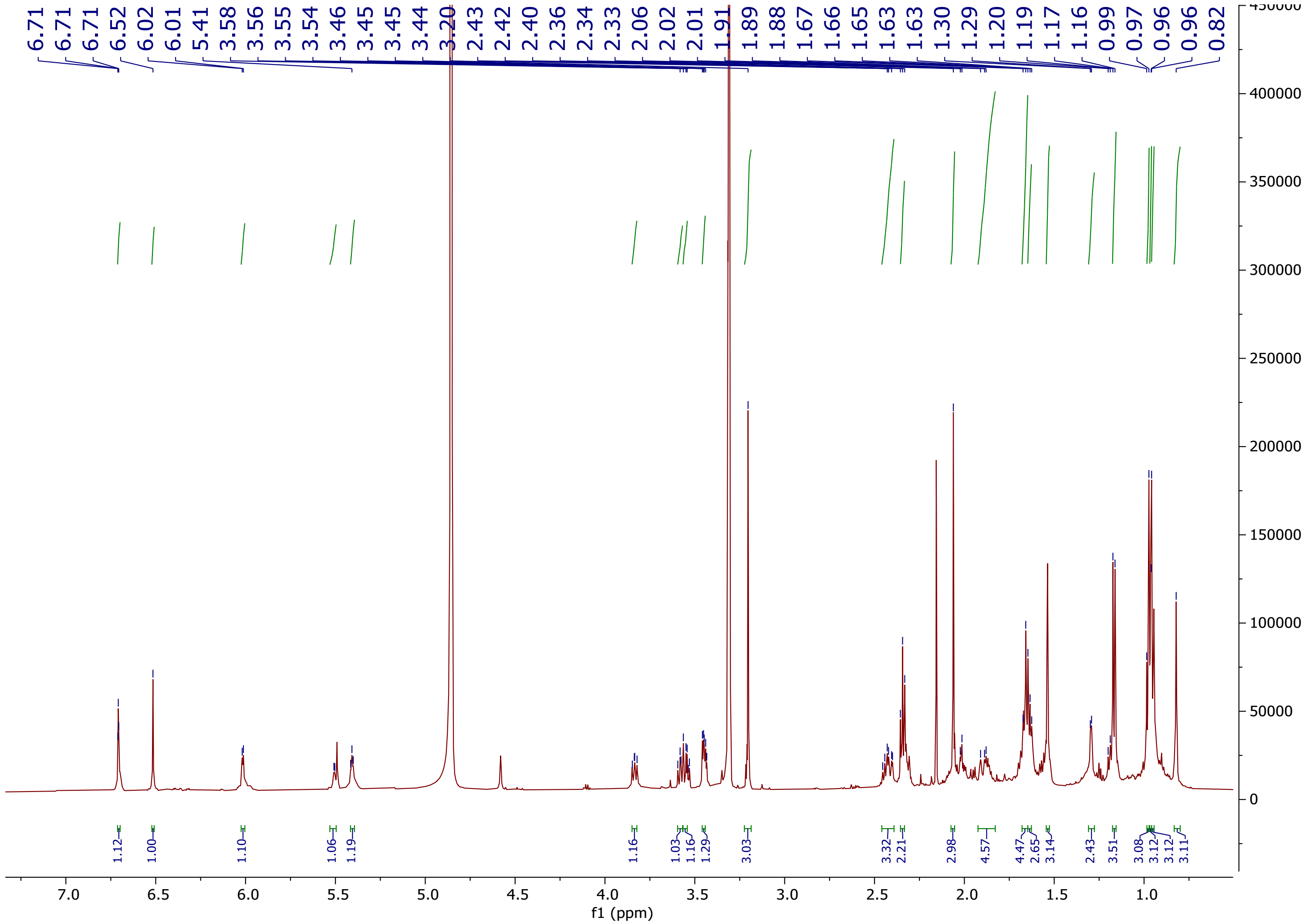

Figure 30: ^{1}H NMR spectrum (500 MHz, methanol-d_4) of compound 4

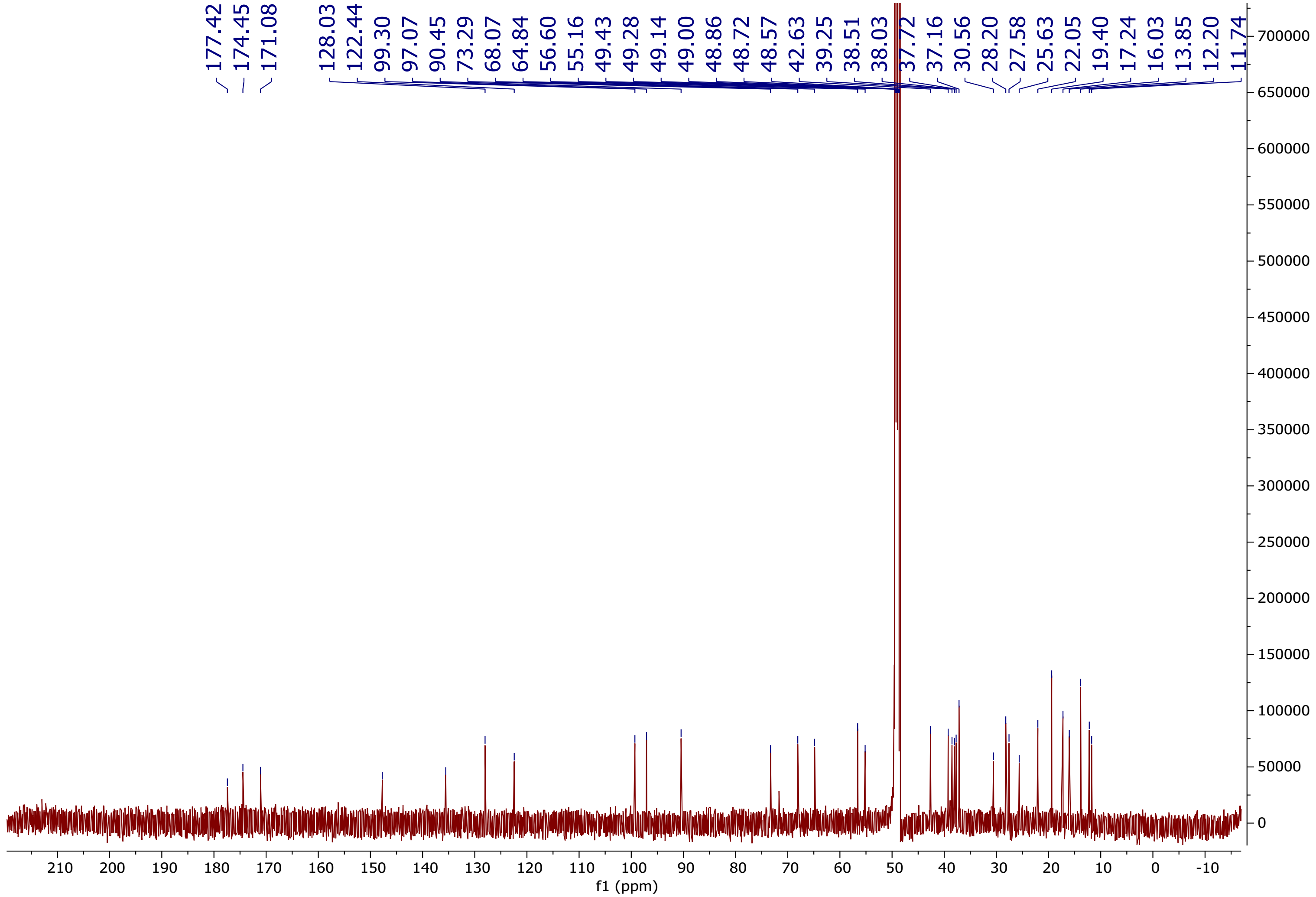

Figure 31: ^{13}C NMR spectrum (125 MHz, methanol-d_4) of compound 4

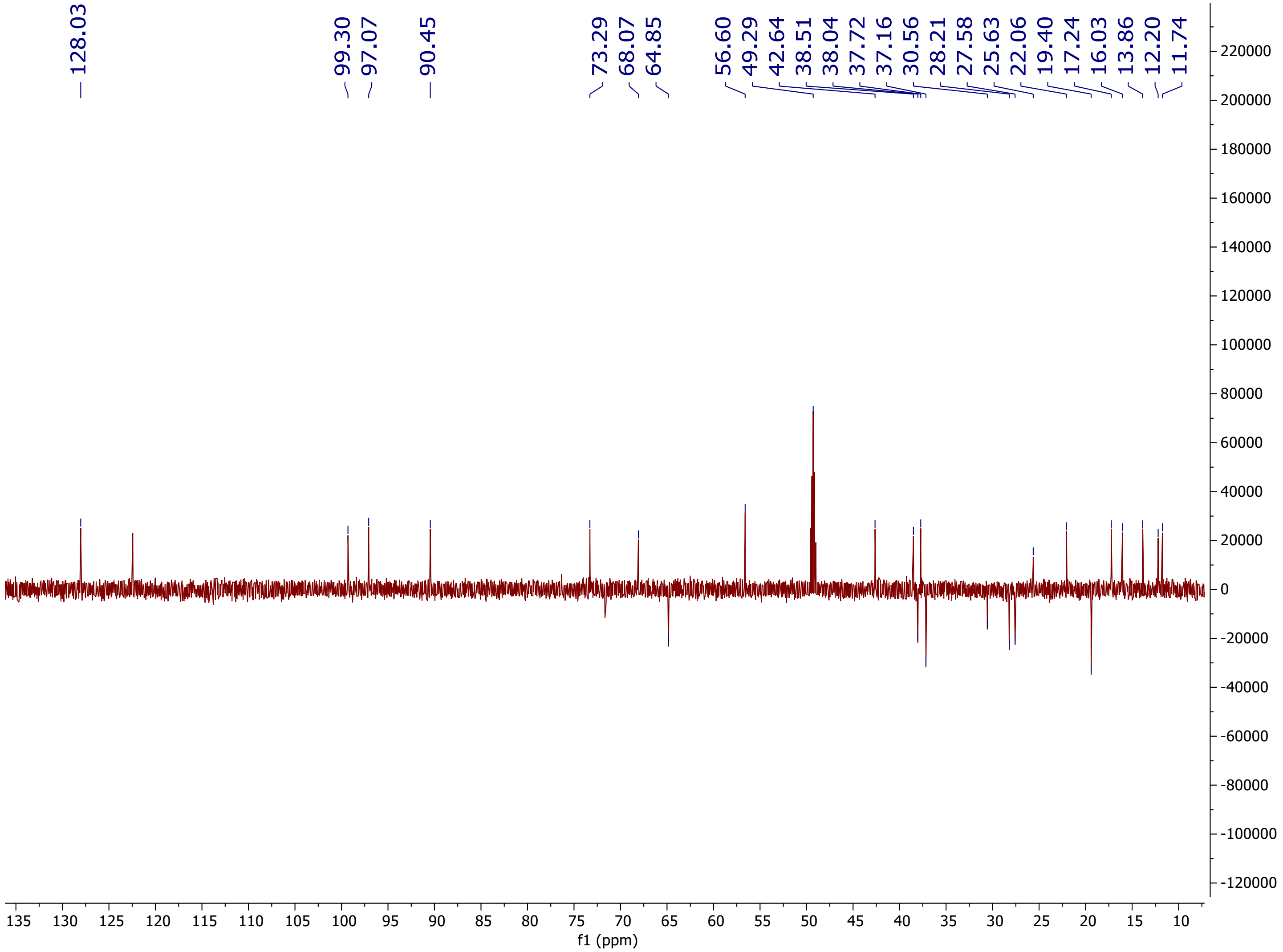

Figure 32: DEPT 135 spectrum (125MHz, methanol-d_4) of compound 4

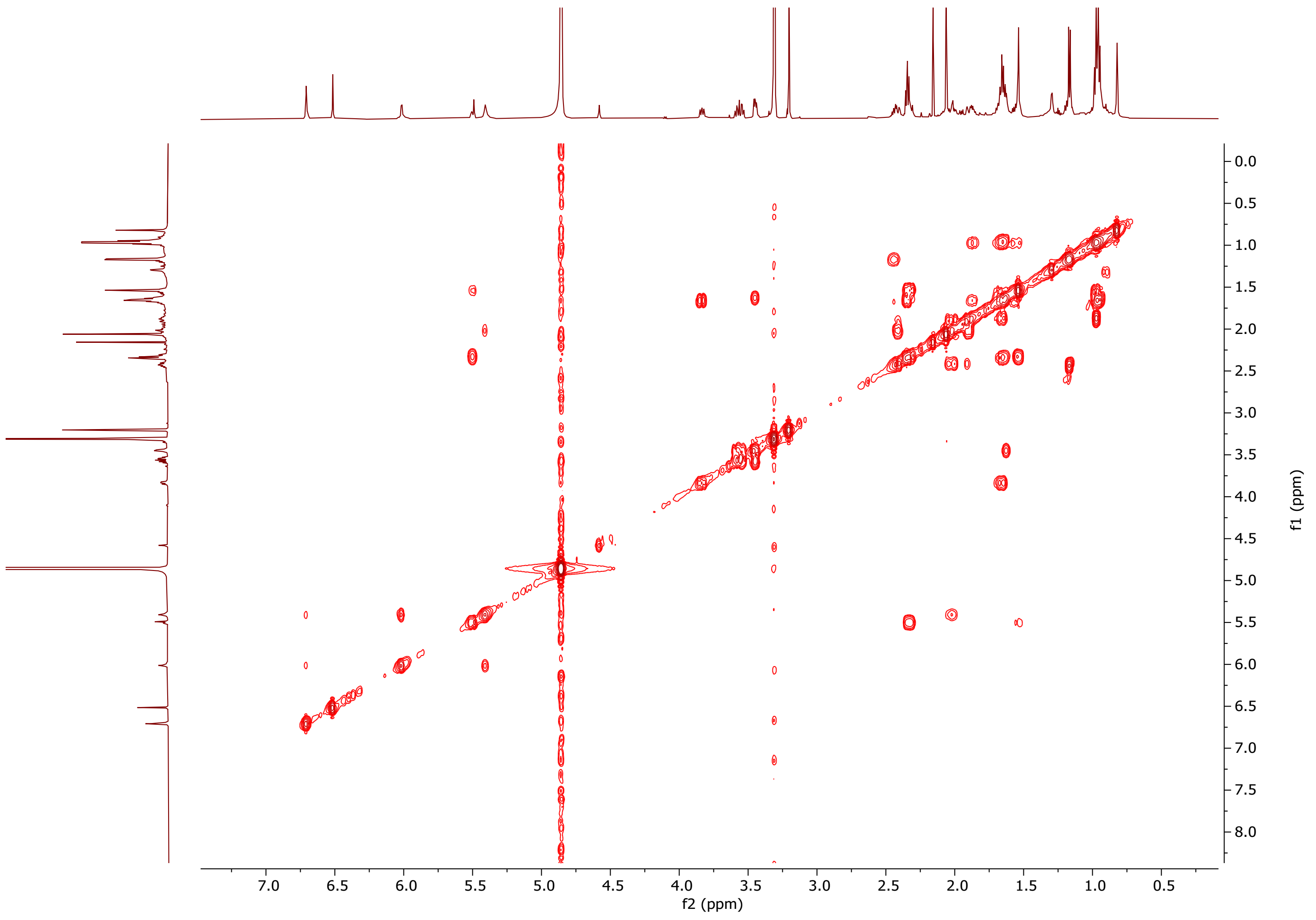

Figure 33: COSY spectrum (500 MHz, methanol-d_4) of compound

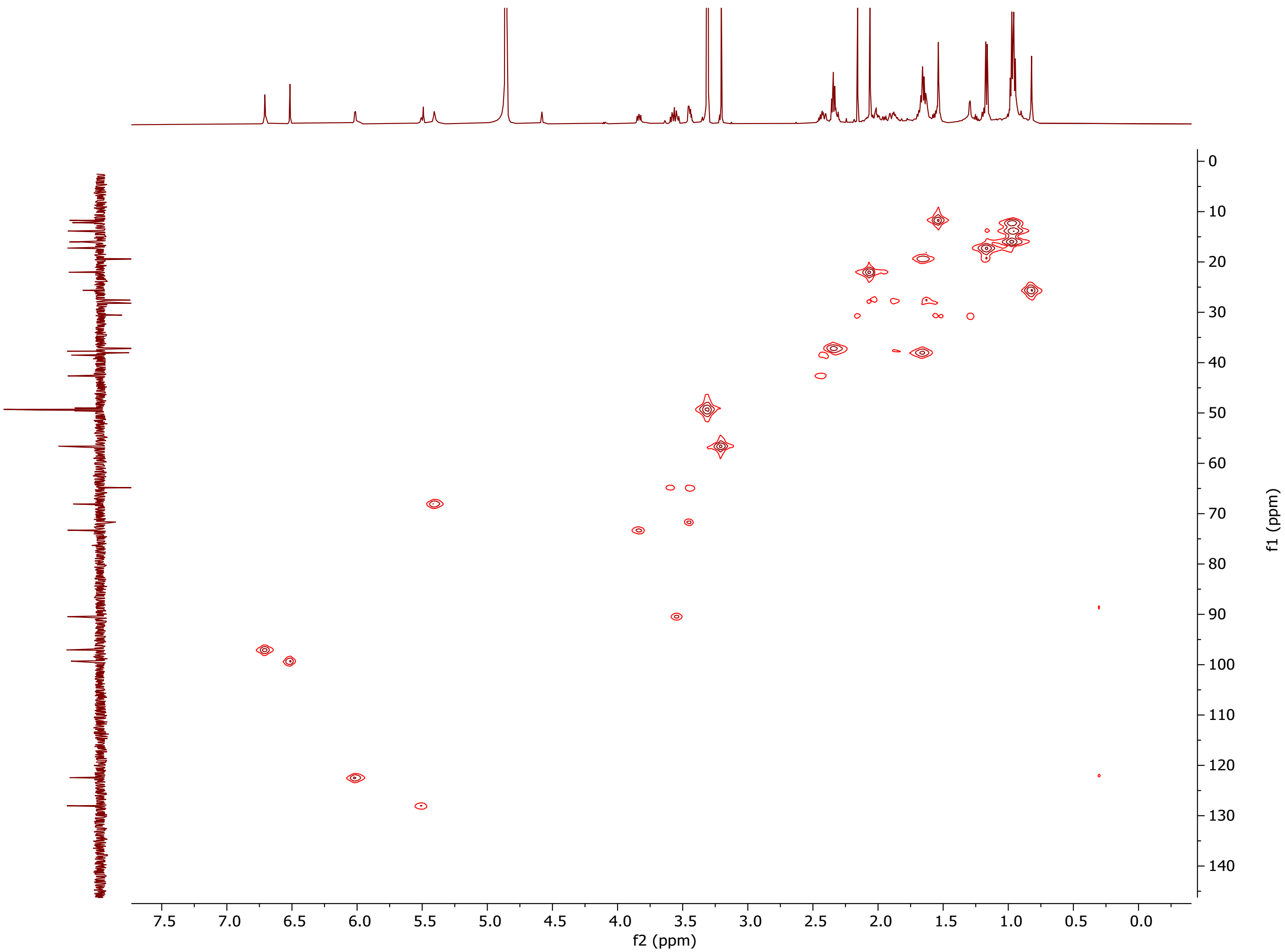

Figure 34: HSQC spectrum (500 MHz, methanol-d_4) of compound 4

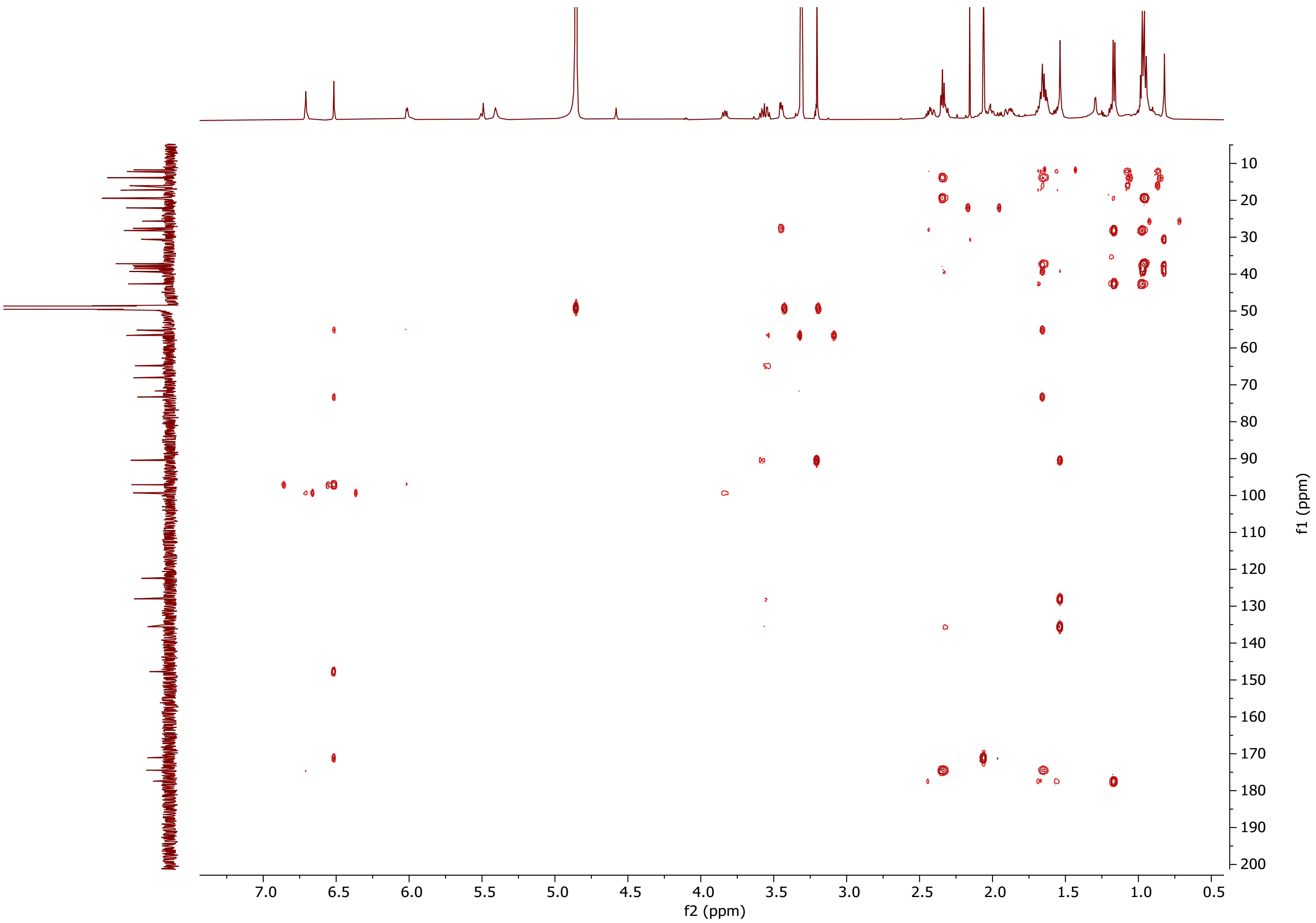

Figure 35: HMBC spectrum (500 MHz, methanol-d_4) of compound 4

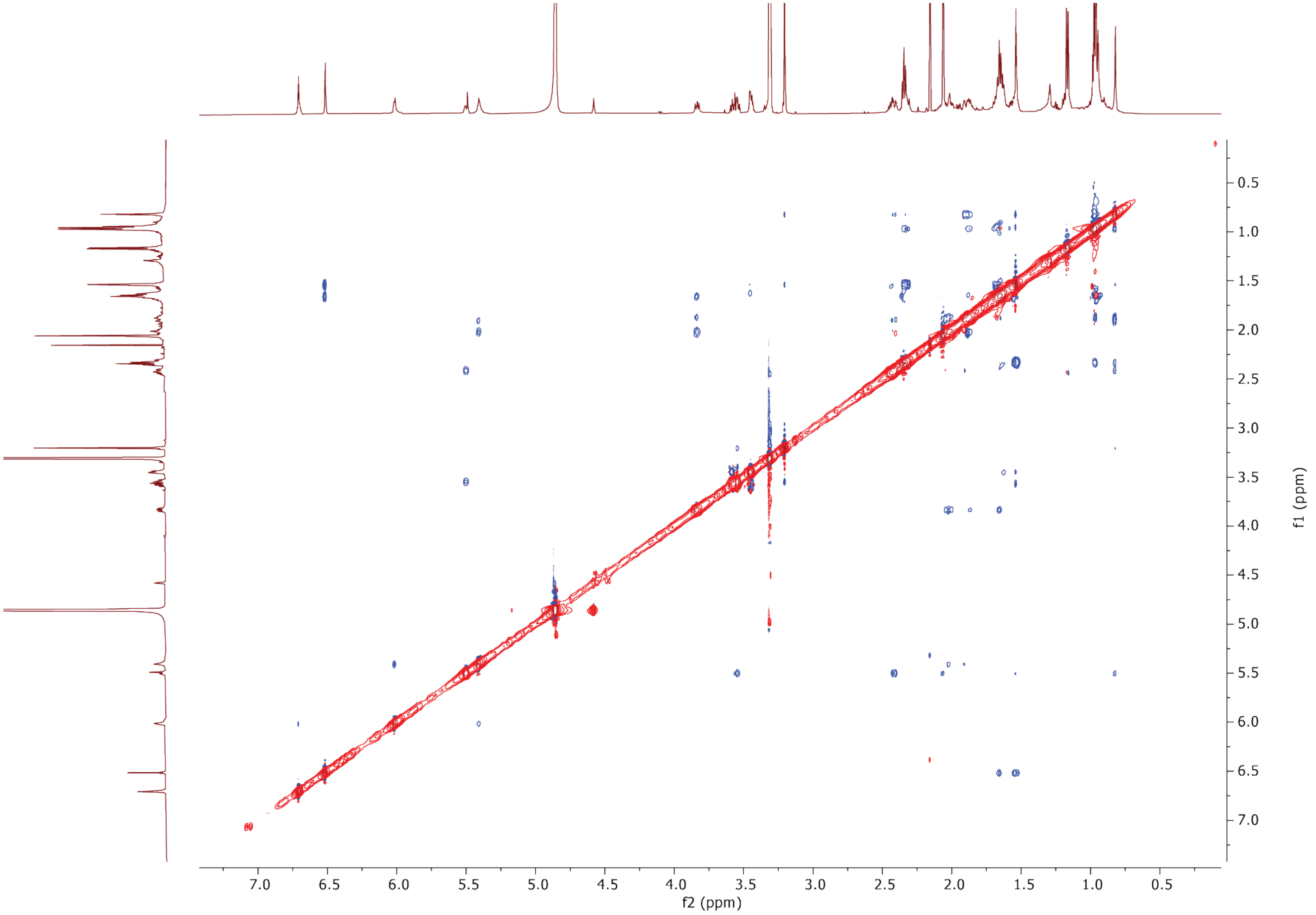

Figure 36: NOESY spectrum (500 MHz, methanol-d_4) of compound 4

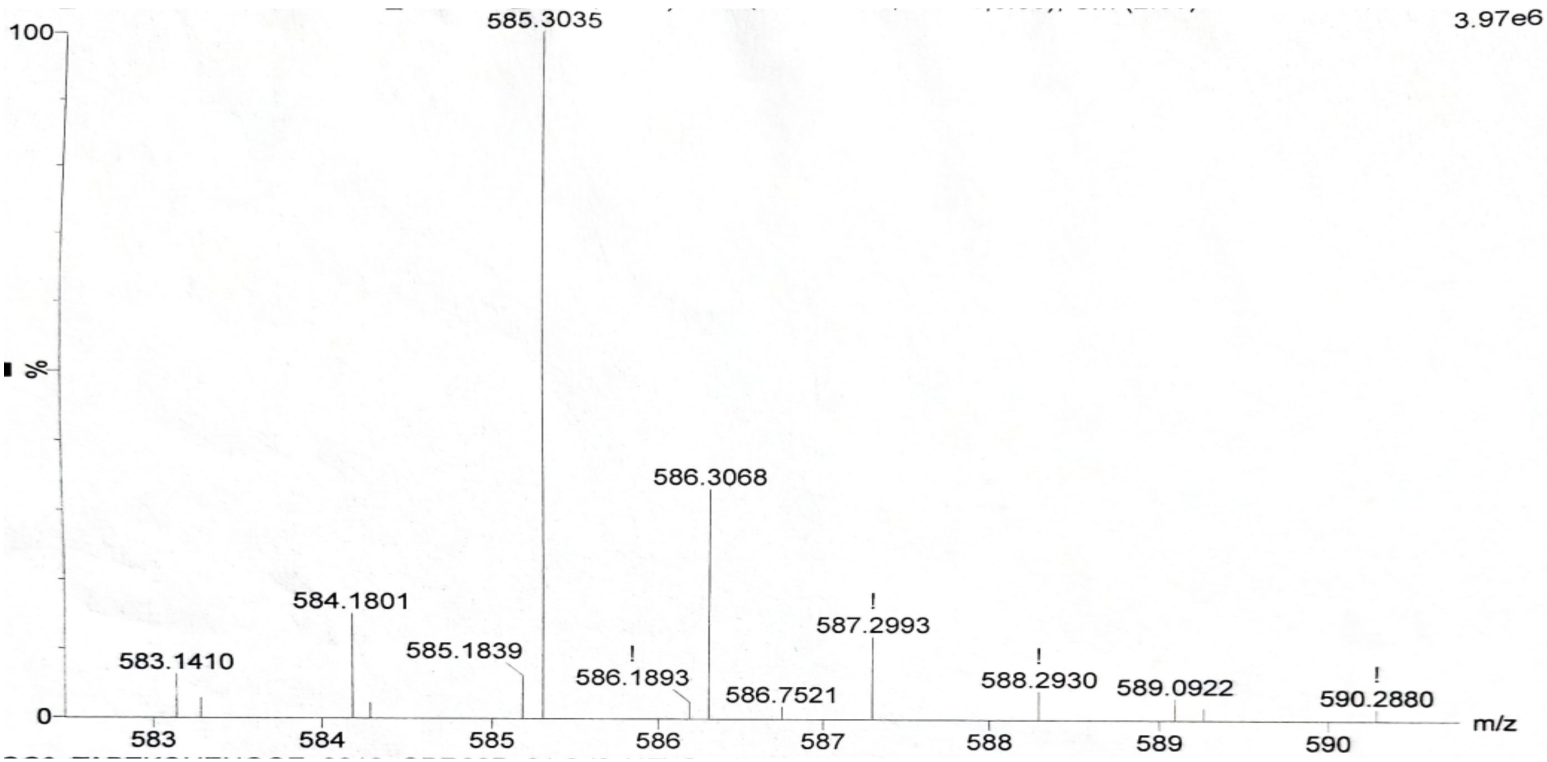

**Measured Ion Mass(es) :**	585.3035	**Deviation [mmu] :**	0.09 [mmu]
**Calculated Ion Mass(es) :**	585.30341	**Deviation [ppm] :**	0.15 [ppm]
**Potential Molecular Formula :**	C31H46O9Na +		

Figure 37: ESI-HR-MS (+) spectrum of compound 5

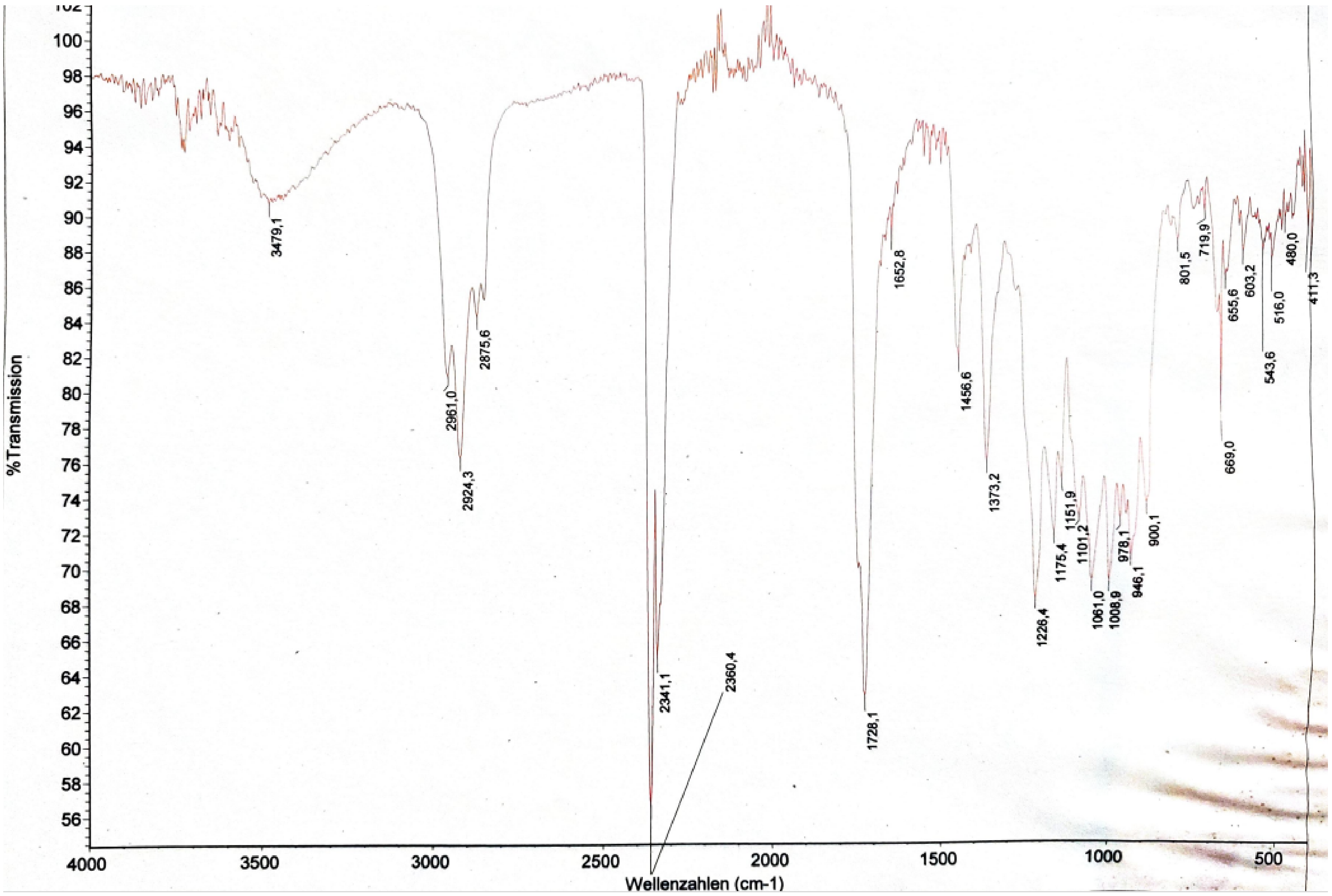

Figure 38: FT-IR spectrum of compound 5

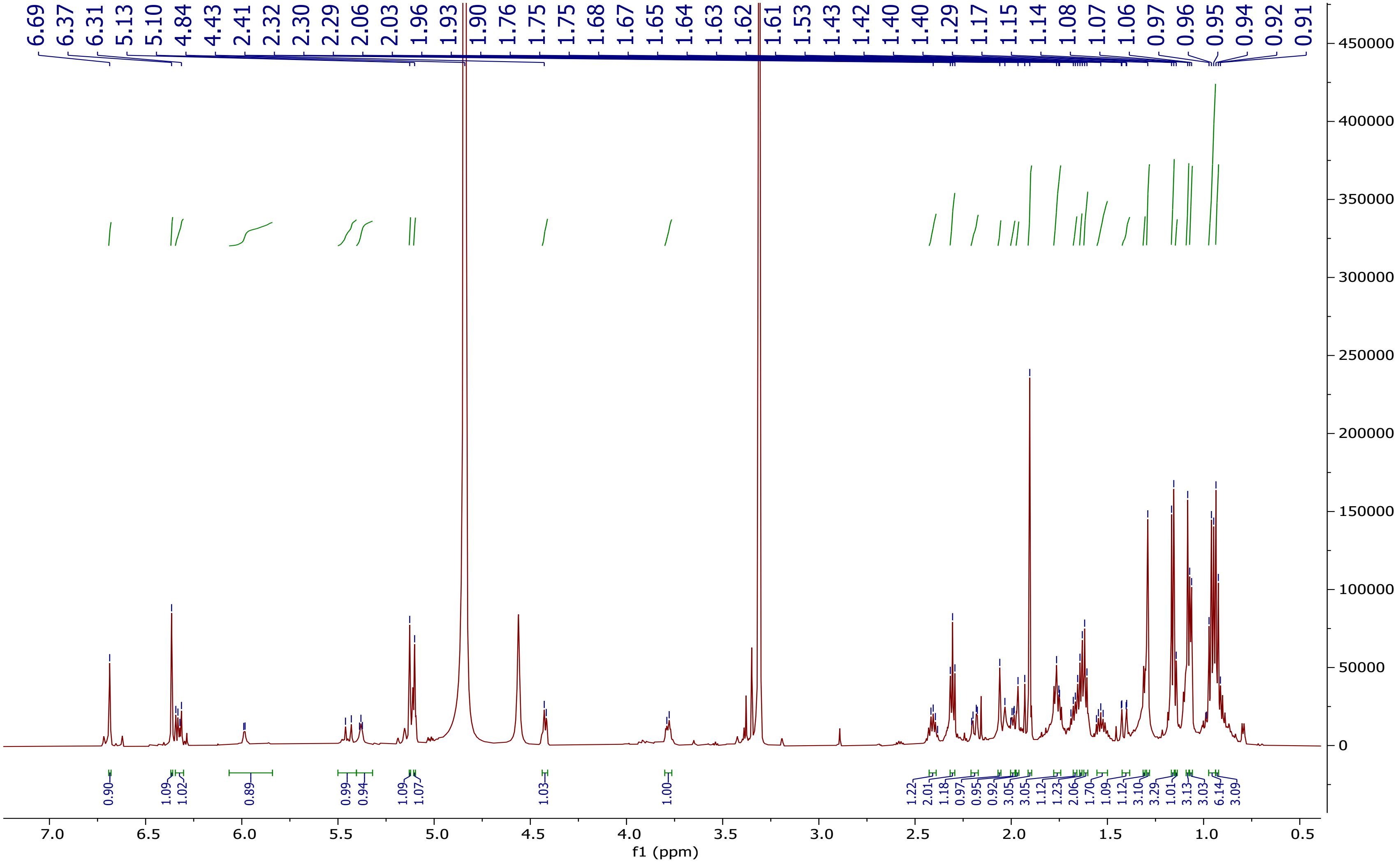

Figure 39: ^{1}H NMR spectrum (500 MHz, methanol-d_4) of compound 5

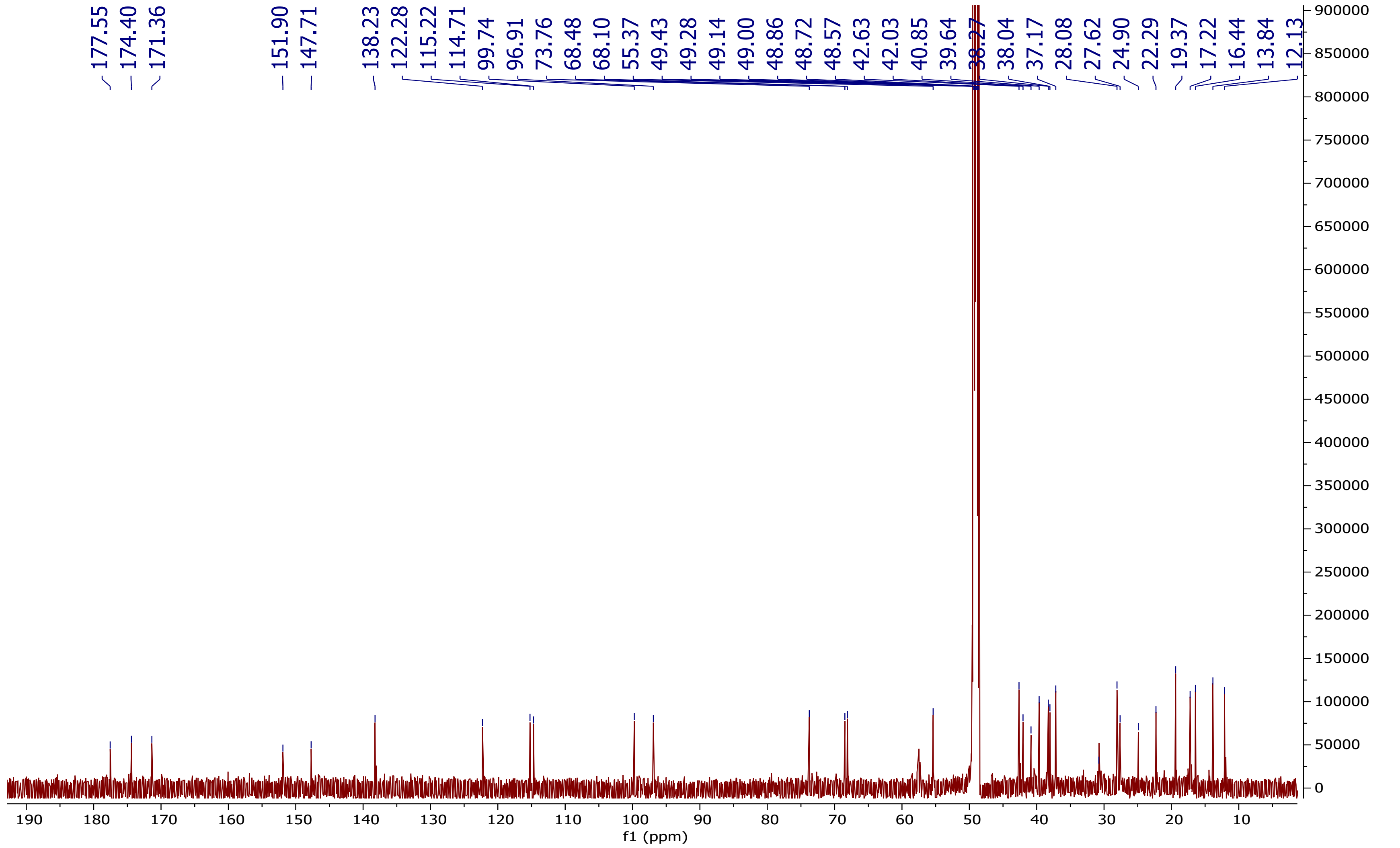

Figure 40: ^{13}C NMR spectrum (125 MHz, methanol-d_4) of compound 5

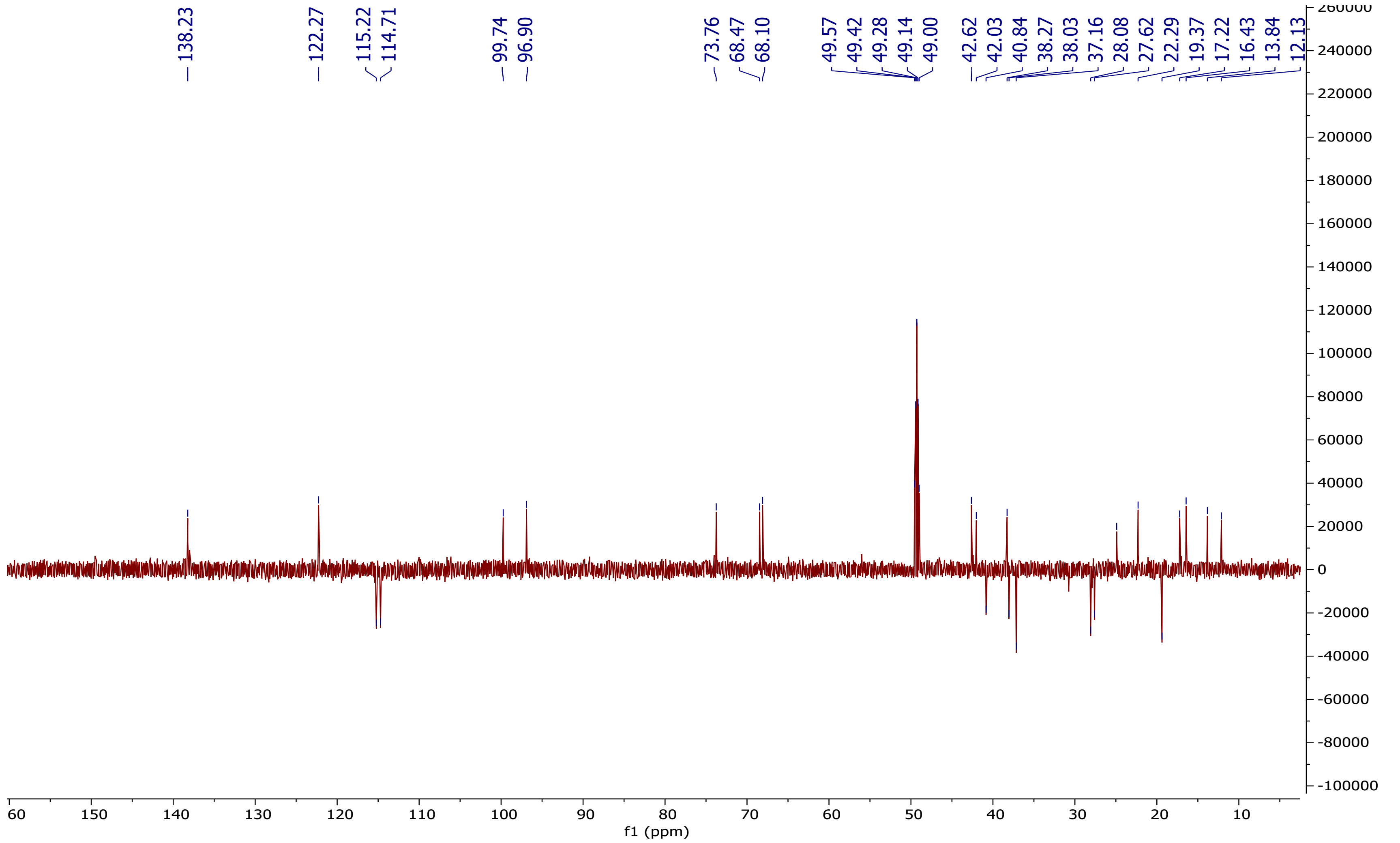

Figure 41: DEPT 135 spectrum (125MHz, methanol-d_4) of compound 5

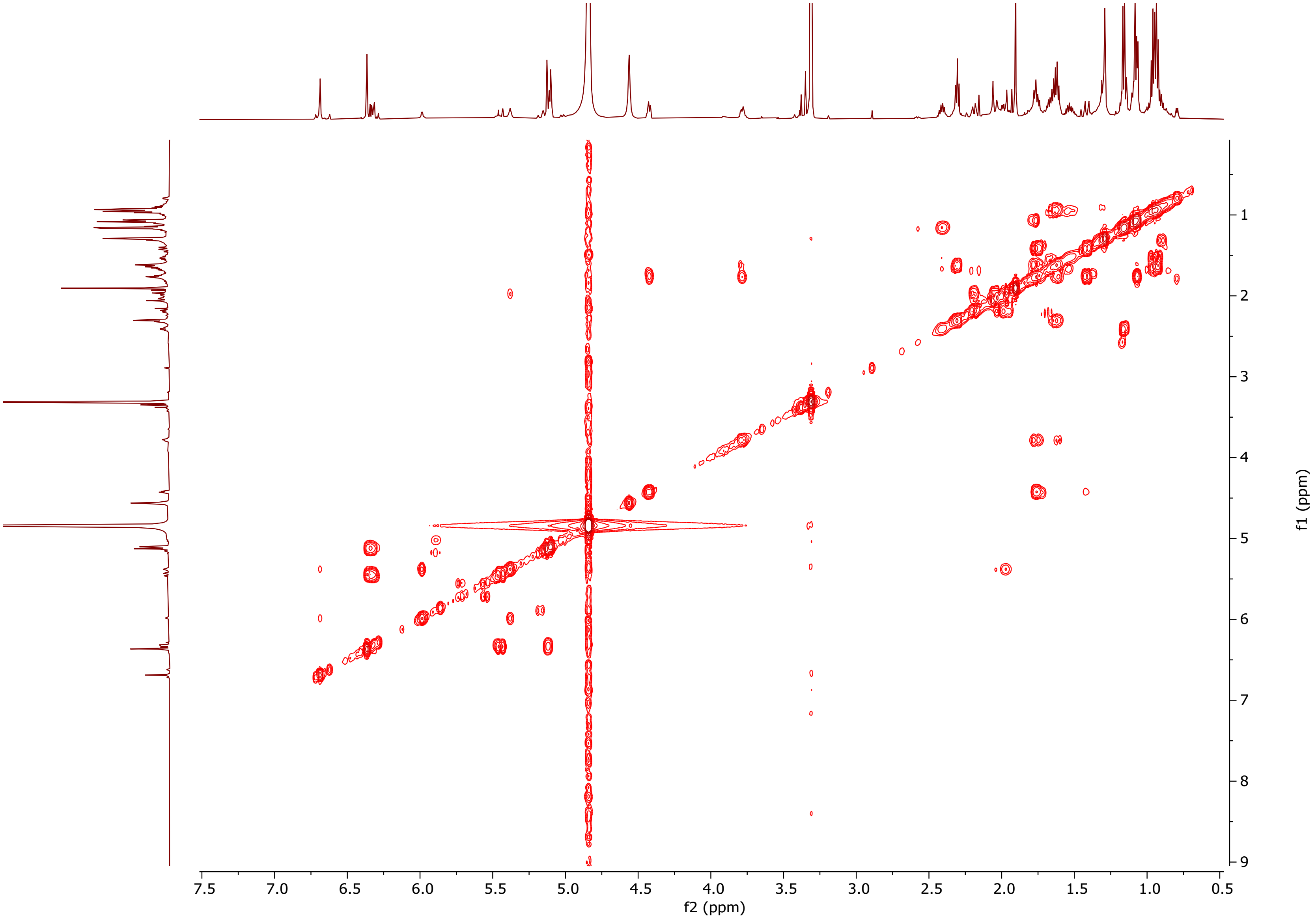

Figure 42: COSY spectrum (500 MHz, methanol-d_4) of compound 5

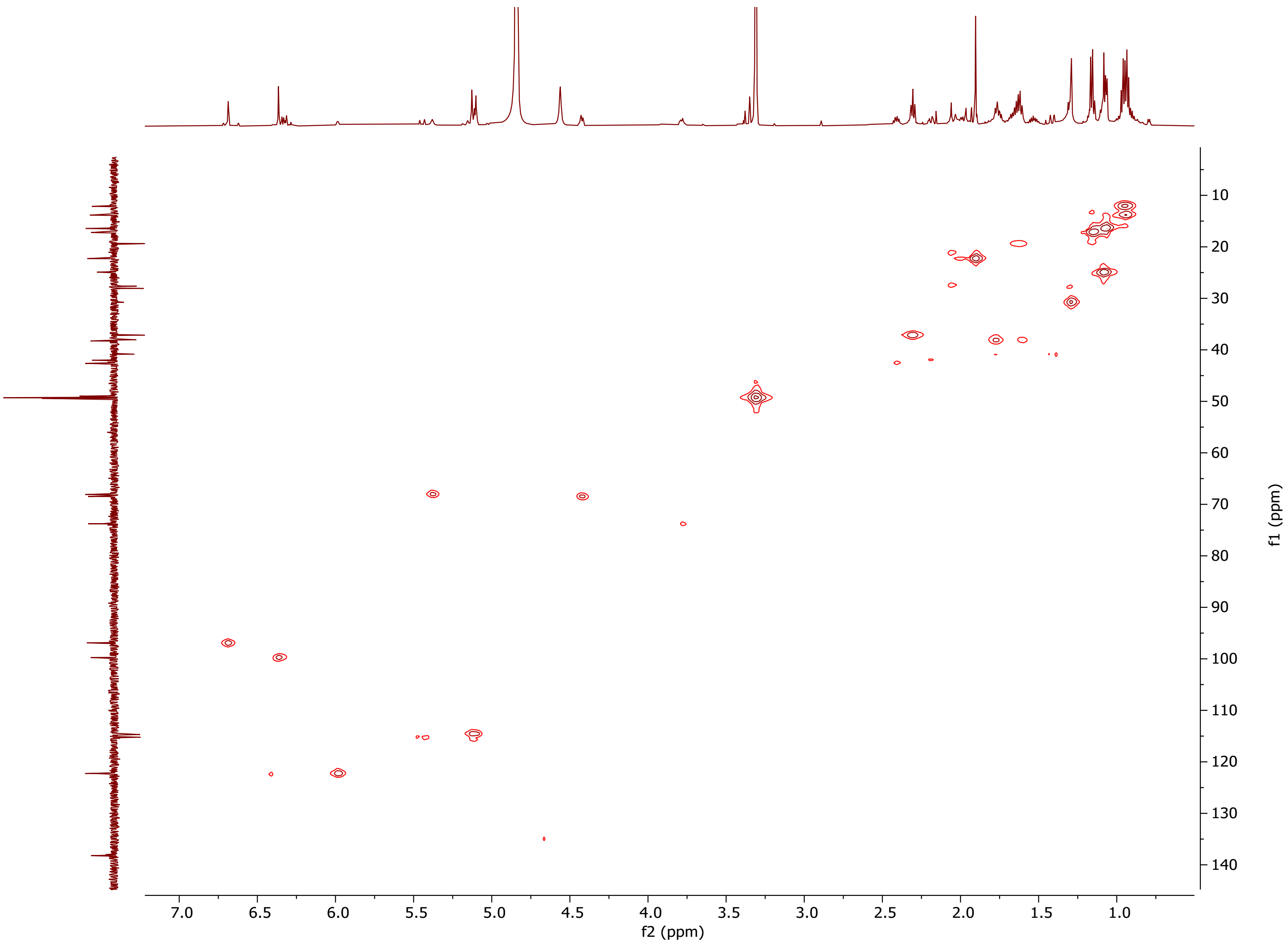

Figure 43: HSQC spectrum (500 MHz, methanol-d_4) of compound 5

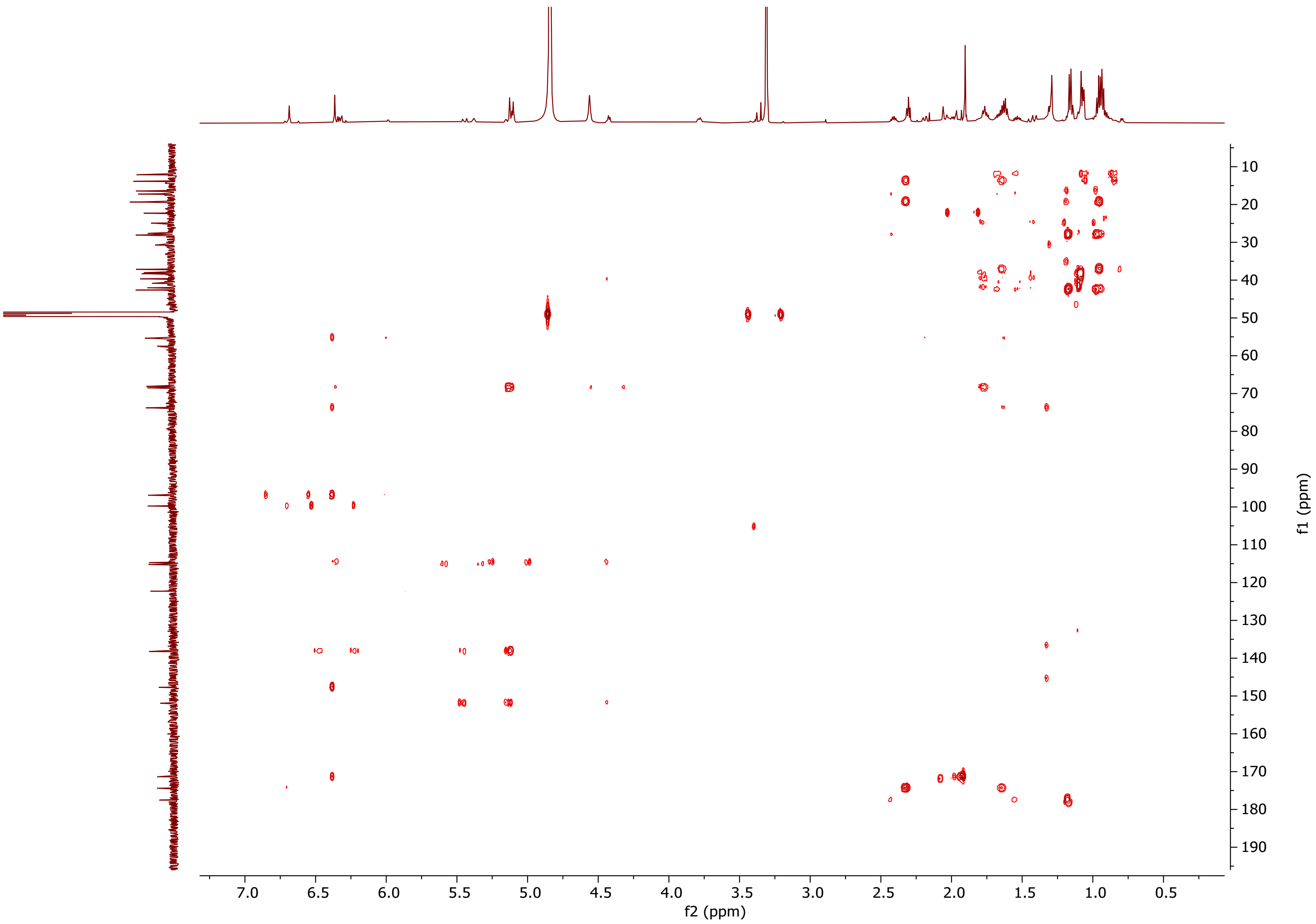

Figure 44: HMBC spectrum (500 MHz, methanol-d_4) of compound 5

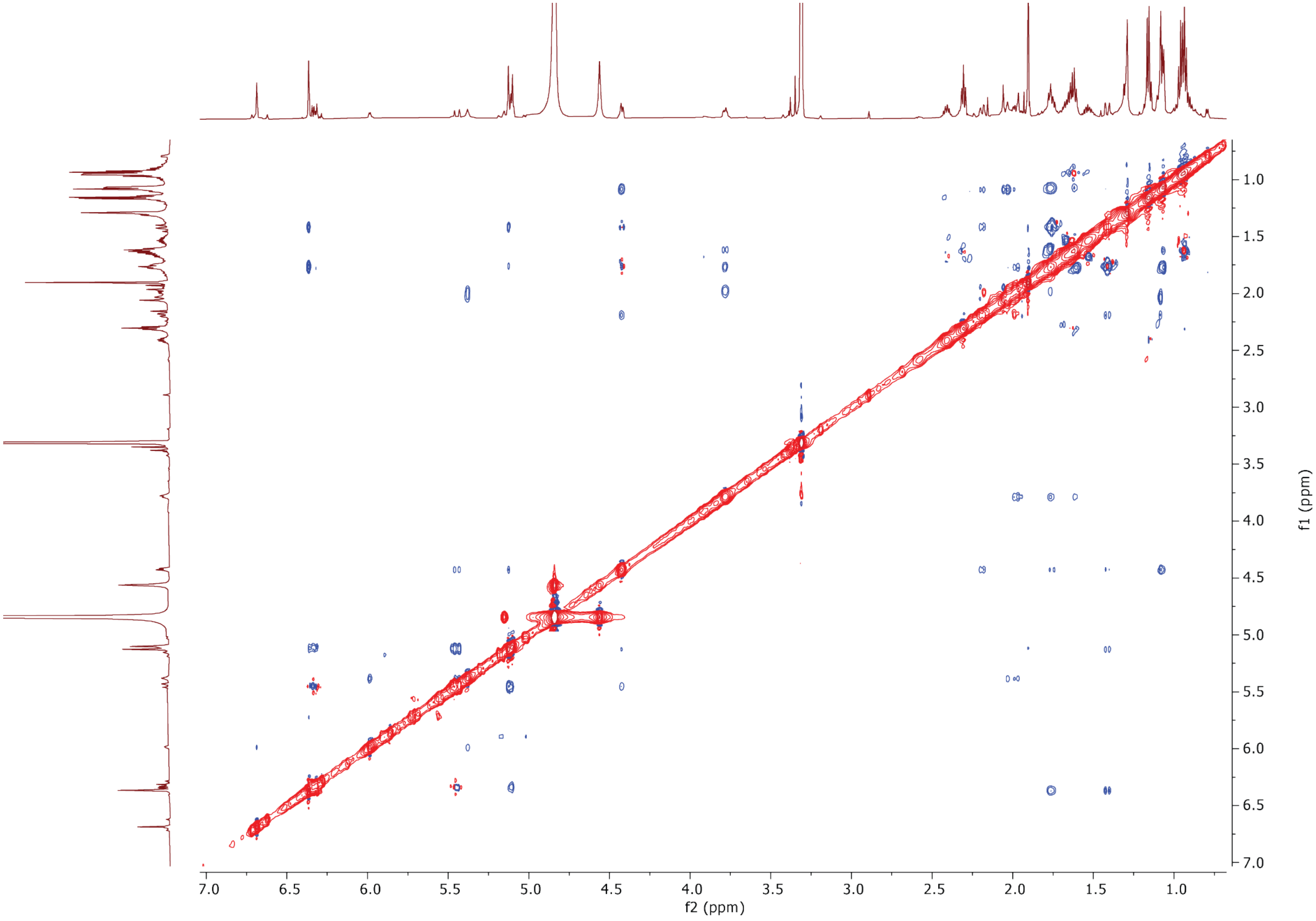

Figure 45: NOESY spectrum (500 MHz, methanol-d_4) of compound 5

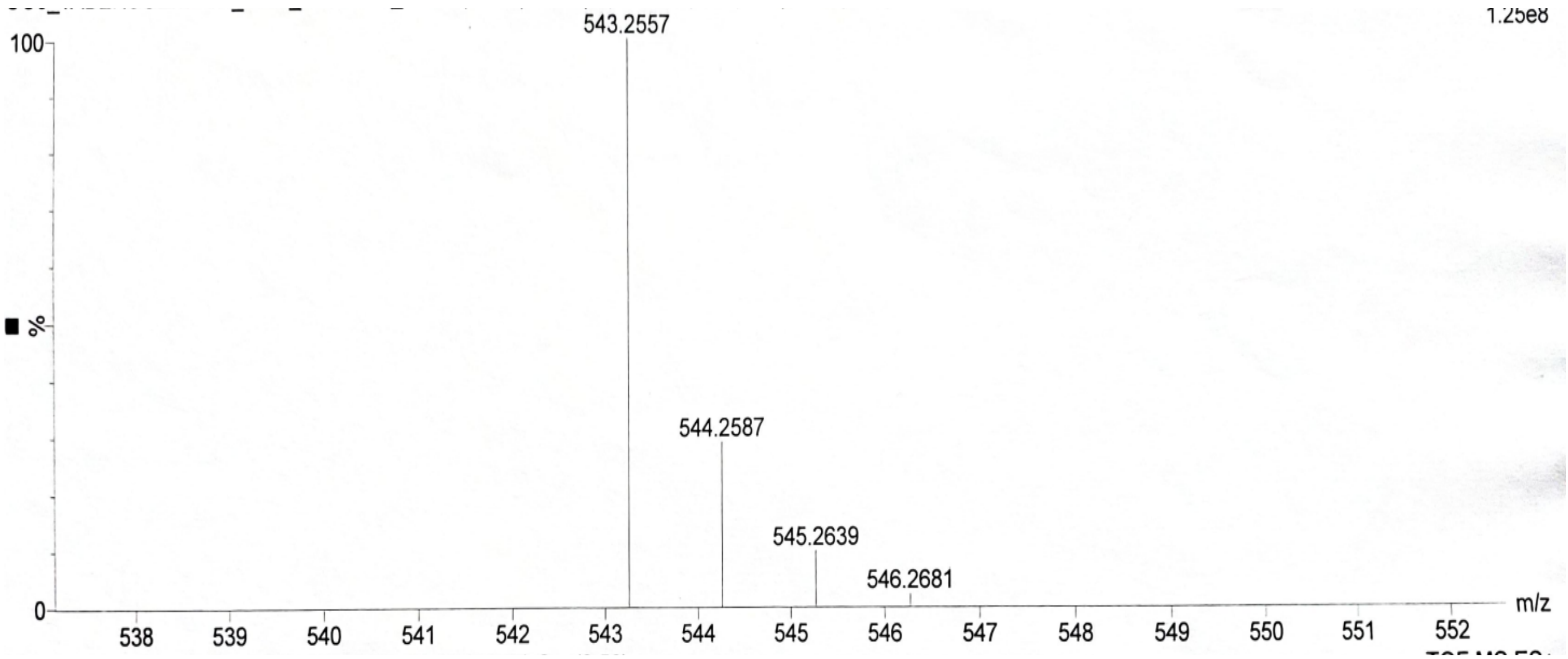

**Measured Ion Mass(es) :**	543.2557	**Deviation [mmu] :**	0.76 [mmu]
**Calculated Ion Mass(es) :**	543.25646	**Deviation [ppm] :**	1.40 [ppm]
**Potential Molecular Formula :**	C28H40O9Na+		

Figure 46: ESI-HR-MS (+) spectrum of compound 6

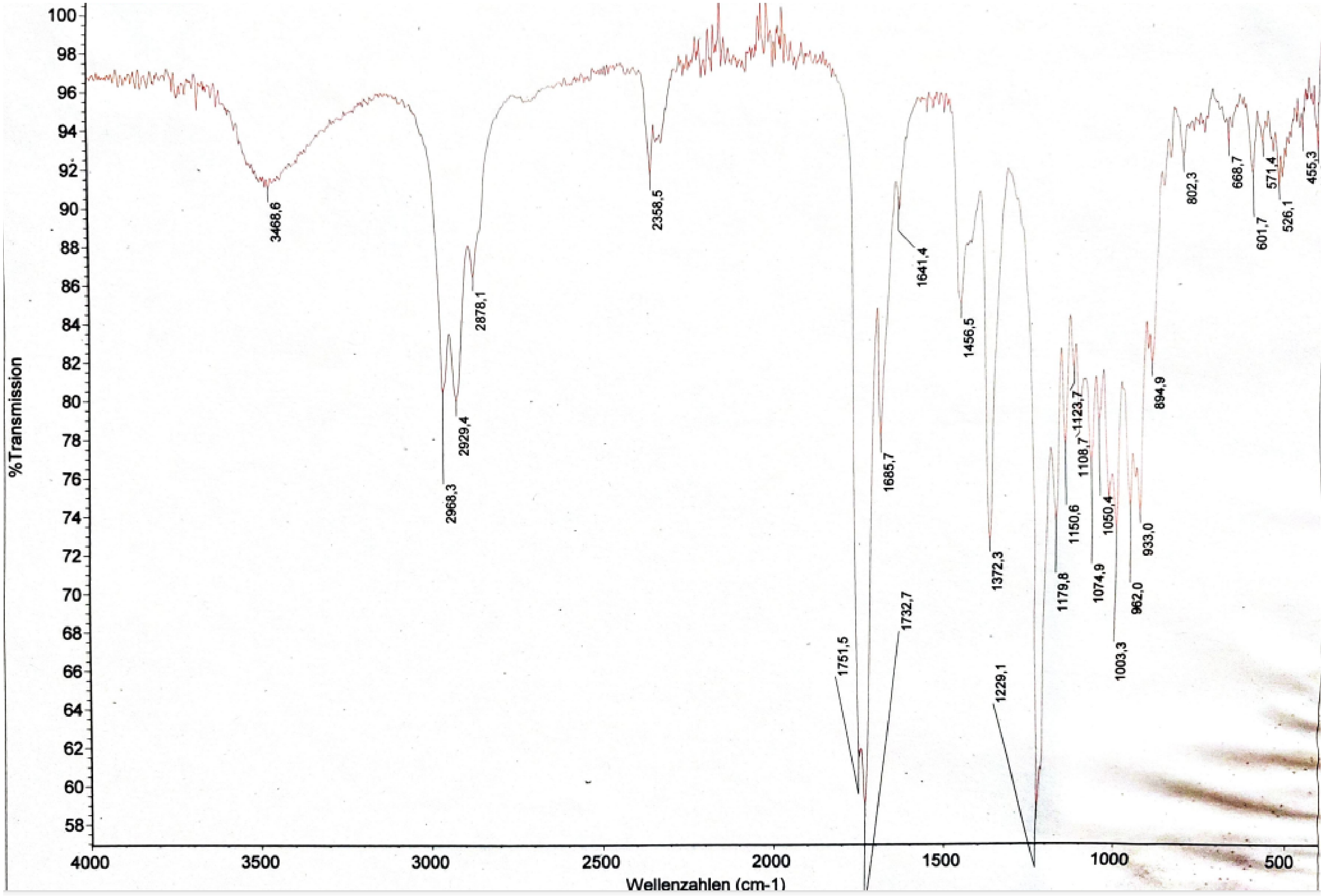

Figure 47: FT-IR spectrum of compound 6

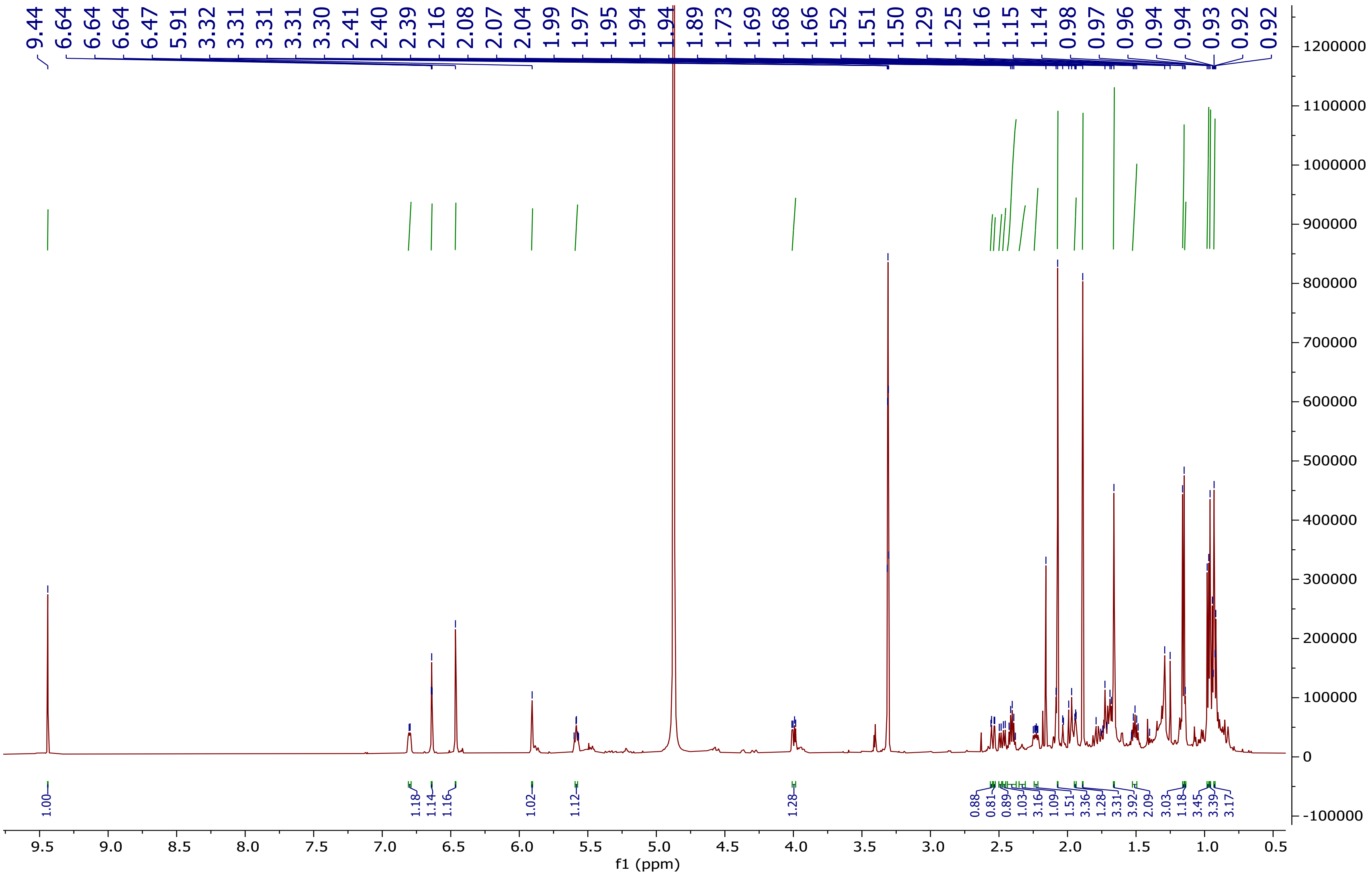

Figure 48: ^{1}H NMR spectrum (500 MHz, methanol-d_4) of compound 6

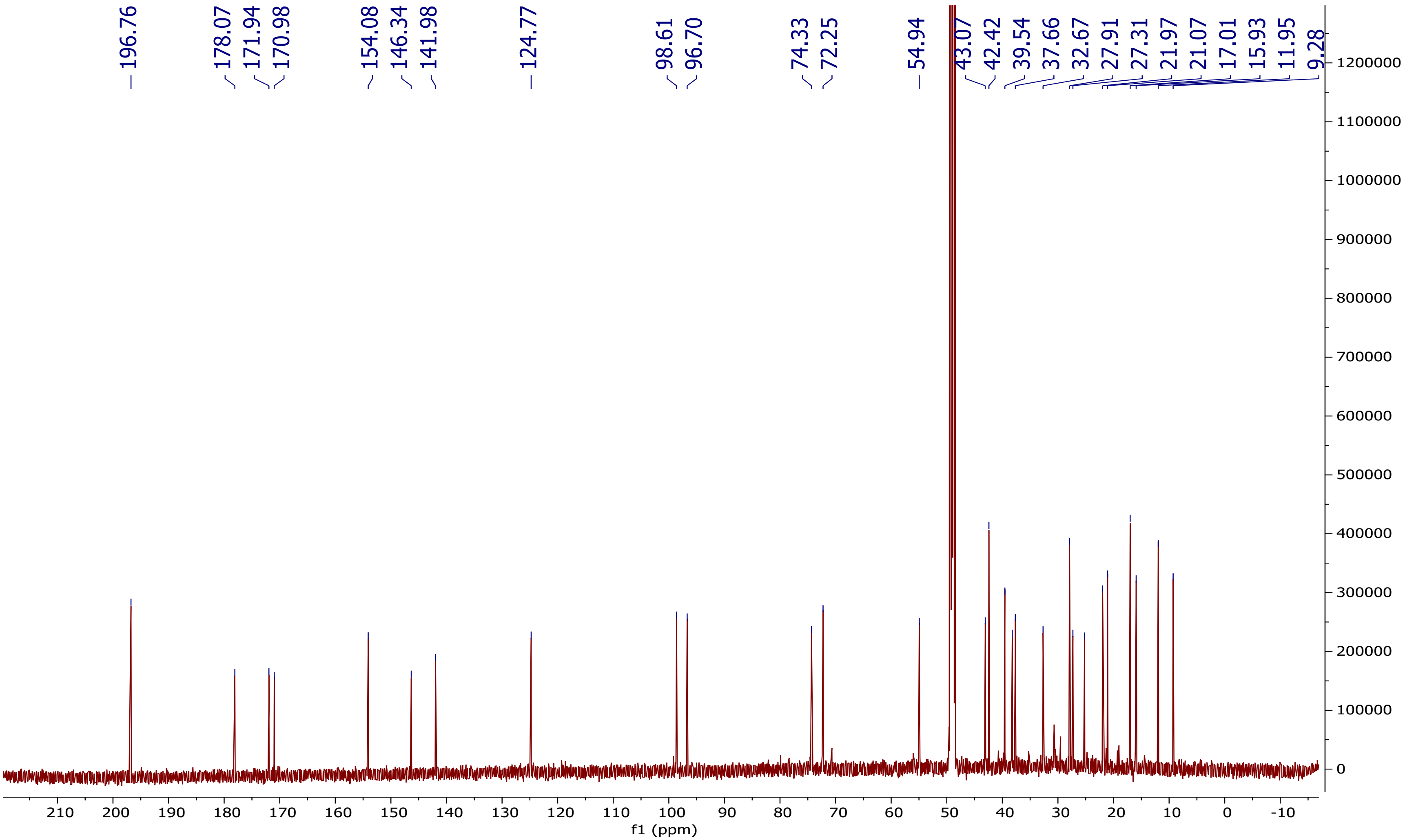

Figure 49: ^{13}C NMR spectrum (125 MHz, methanol-d_4) of compound 6

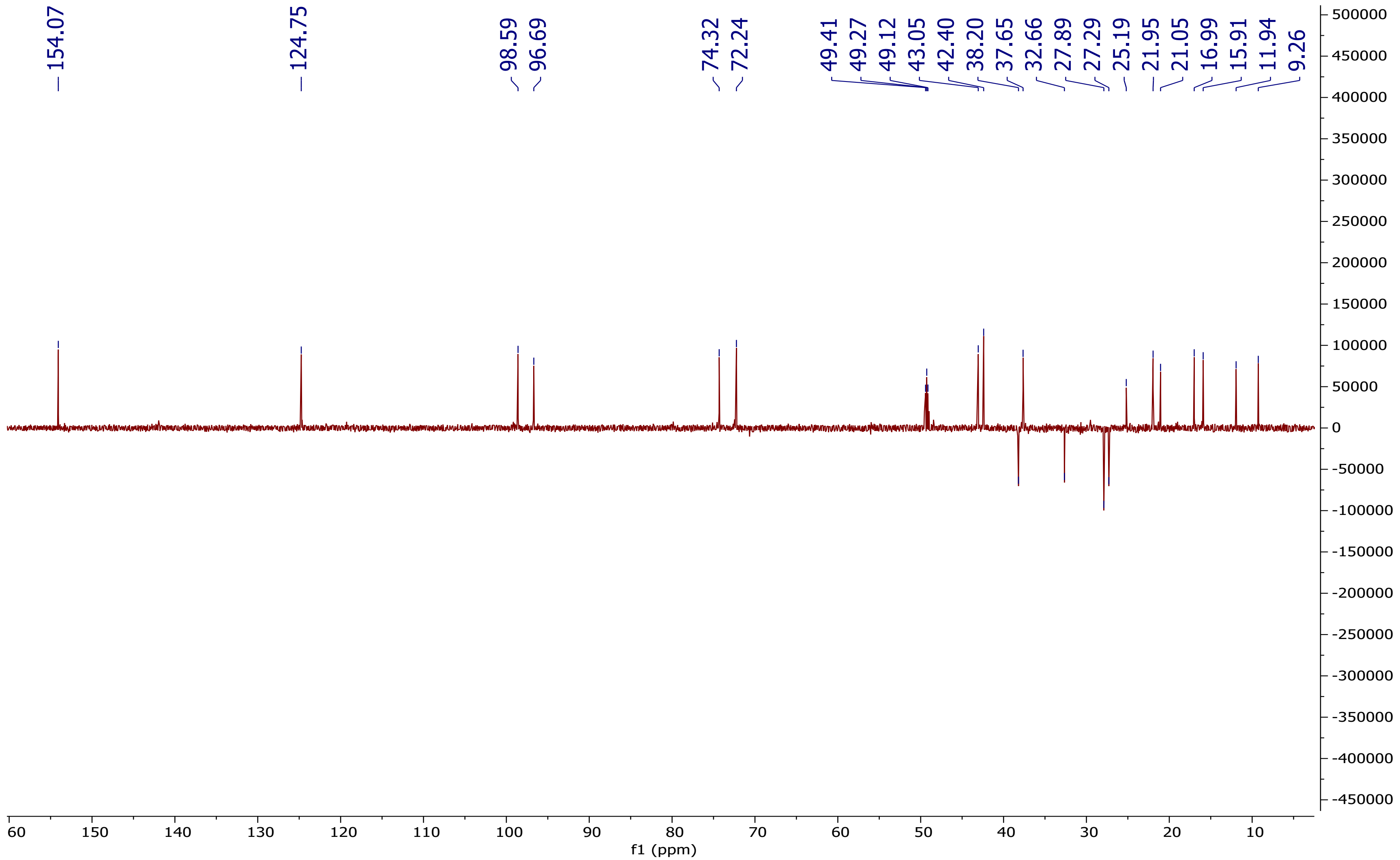

Figure 50: DEPT 135 spectrum (125MHz, methanol-d_4) of compound 6

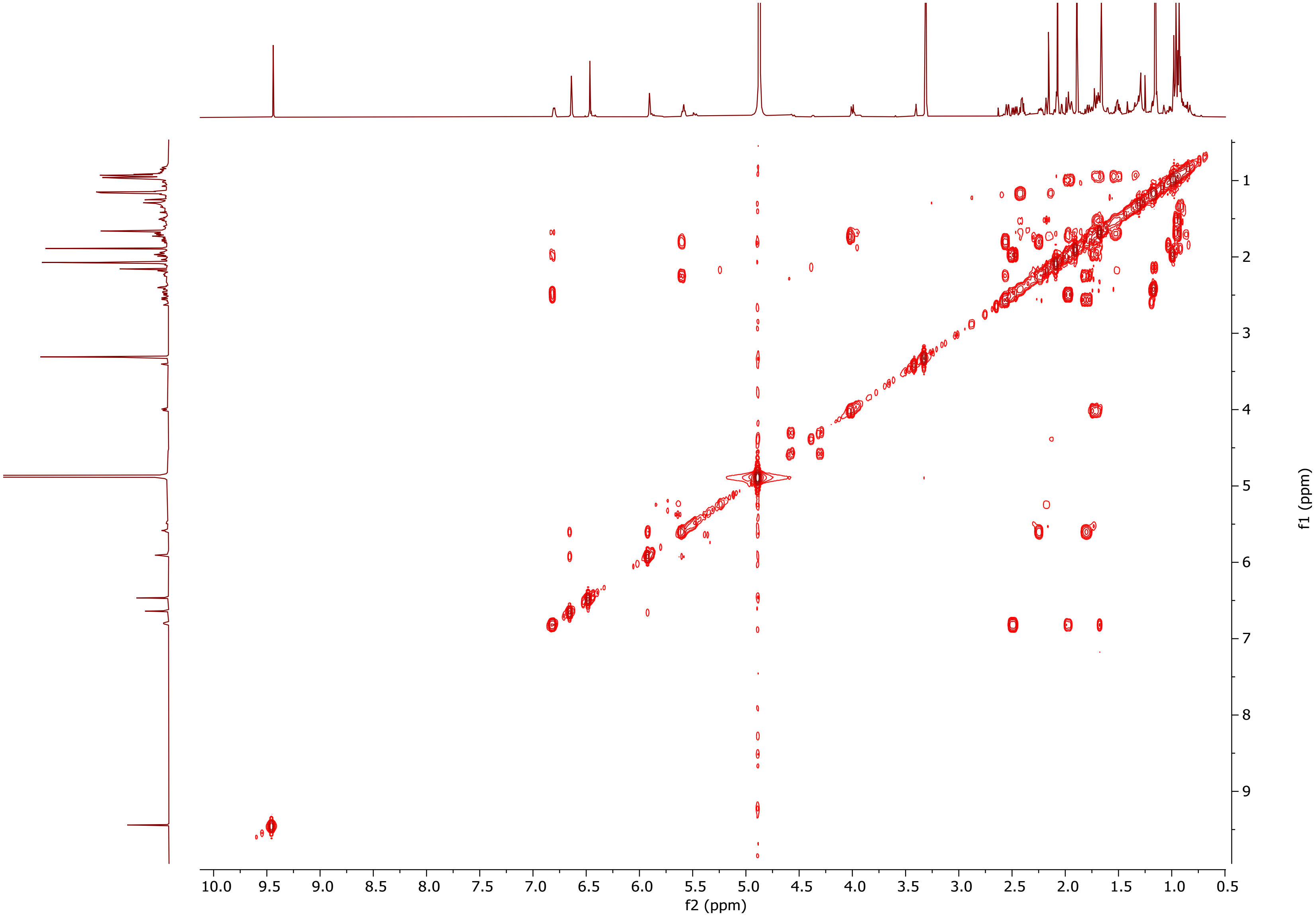

Figure 51: COSY spectrum (500 MHz, methanol-d_4) of compound 6

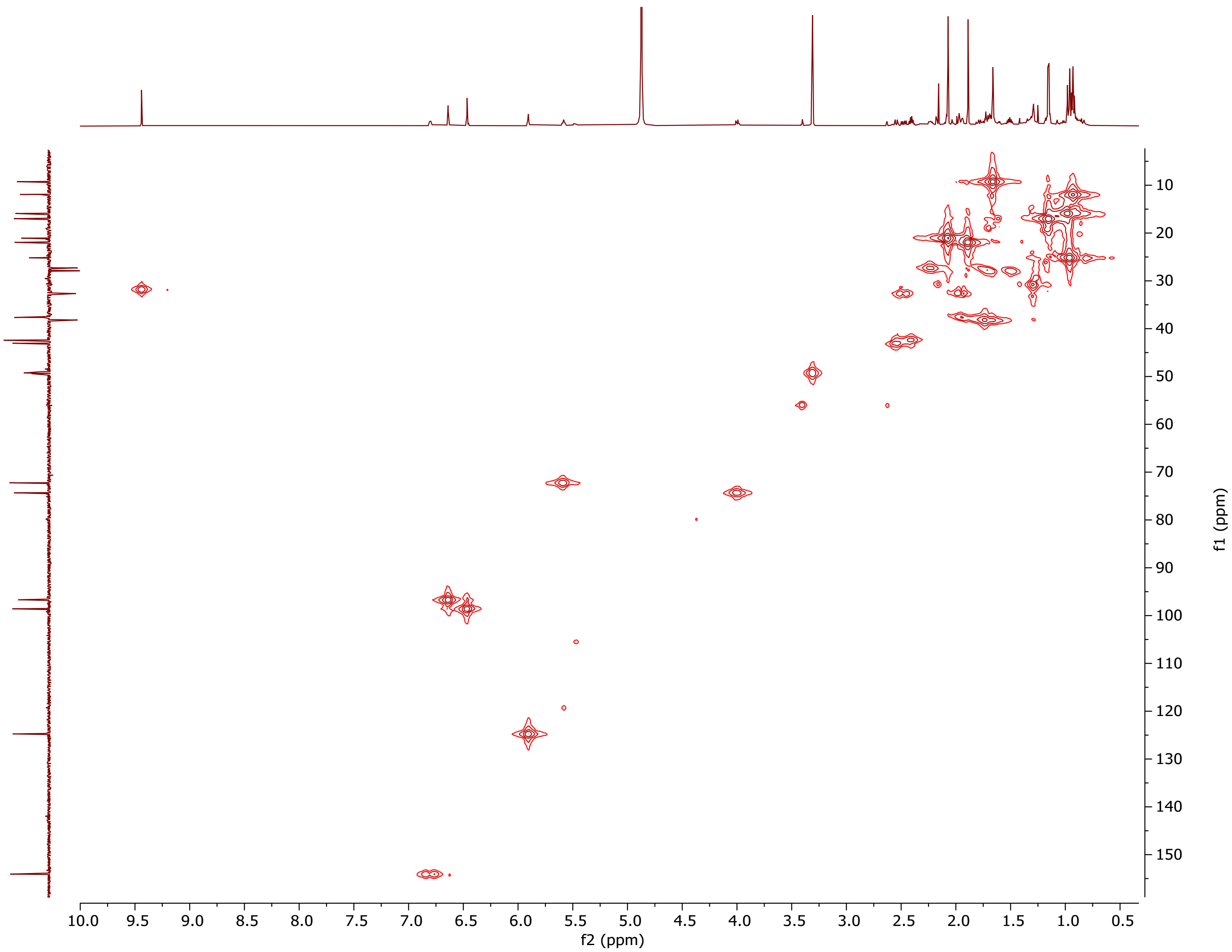

Figure 52: HSQC spectrum (500 MHz, methanol-d_4) of compound 6

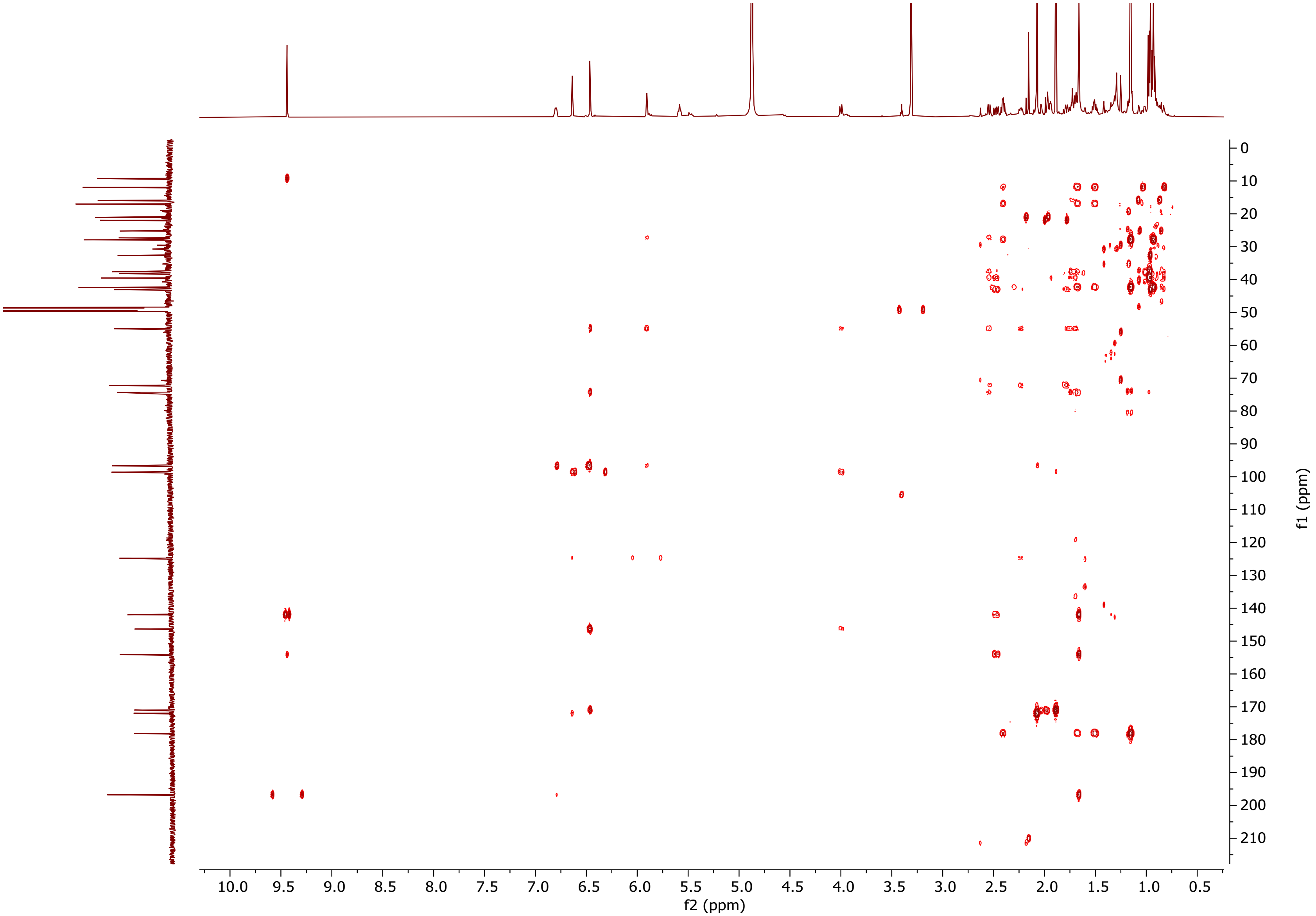

Figure 53: HMBC spectrum (500 MHz, methanol-d_4) of compound 6

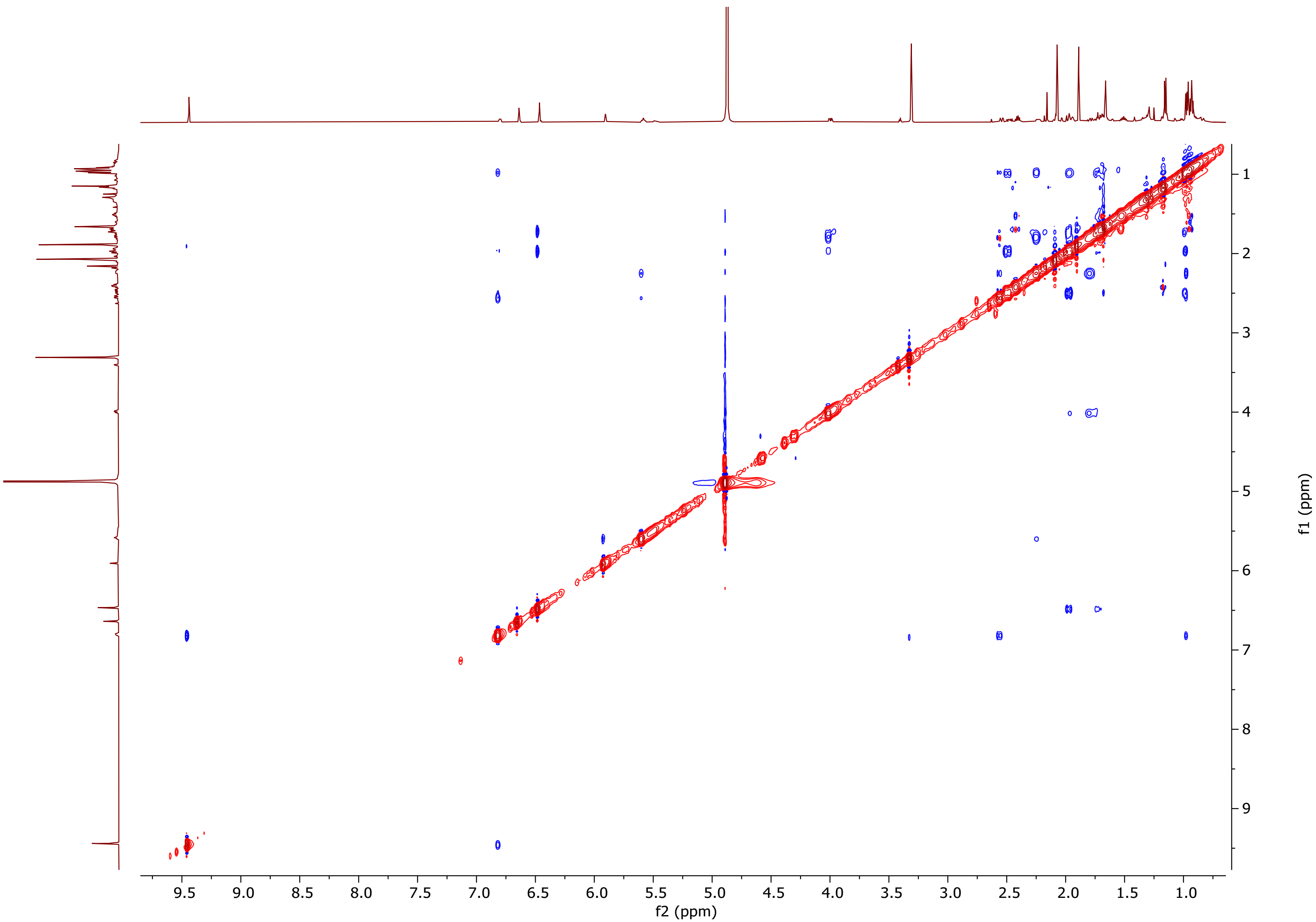

Figure 54: NOESY spectrum (500 MHz, methanol-d_4) of compound 6

Experimental detail for cytotoxic assay.

Cytotoxic activity screening of the crude extract and compounds was performed according to a previously described protocol by Sammet et al., 2010. The KB-3-1 and KB-V1 cells were cultivated as a monolayer in DMEM (Dulbecco's modified Eagle medium) with glucose (4.5 g L^{-1}), L-glutamine, sodium pyruvate and phenol red, supplemented with 10 % (KB-3-1) and 15 % (KB-V1) foetal bovine serum (FBS). 50 µg mL^{-1} gentamycin was added for the KB-V1 cells. The cells were maintained at 37 °C and 5.3 % CO_2-humidified air. KB-V1 cells were continuously selected during cultivation with vinblastine sulfate (150 nM). On the day before the test, the cells (70 % confluence) were detached with trypsin-ethylenediamine tetraacetic acid (EDTA) solution (0.05 %; 0.02 % in DPBS) and placed in sterile 96-well plates in a density of 10 000 cells in 100 µL medium per well. The dilution series of the compounds were prepared from stock solutions in DMSO of concentrations of 1 mM or 10 mM. The stock solutions were diluted with culture medium (15 % FBS [KB-V1]; 10 % FBS [KB-3-1]) at least 50 times. Some culture medium was added to the wells to adjust the volume of the wells to the wanted dilution factor. The dilution prepared from stock solution was added to the wells. Each concentration was tested in six replicates. Dilution series were prepared by pipetting liquid from well to well. The control contained the same concentration of DMSO as the first dilution. After incubation for 72 h at 37 ◦ C and 5.3 % CO_2-humidified air, 30 µL of an aqueous resazurin solution (175 µM) was added to each well. The cells were incubated at the same conditions for 6 h. Subsequently, the fluorescence was measured. The excitation was effected at a wavelength of 530 nm, whereas the emission was recorded at a wavelength of 588 nm. The IC_{50} values were calculated as a sigmoidal dose response curve using GRAPHPAD PRISM 4.03. The IC_{50} values equal the drug concentrations, at which vitality is 50 %.

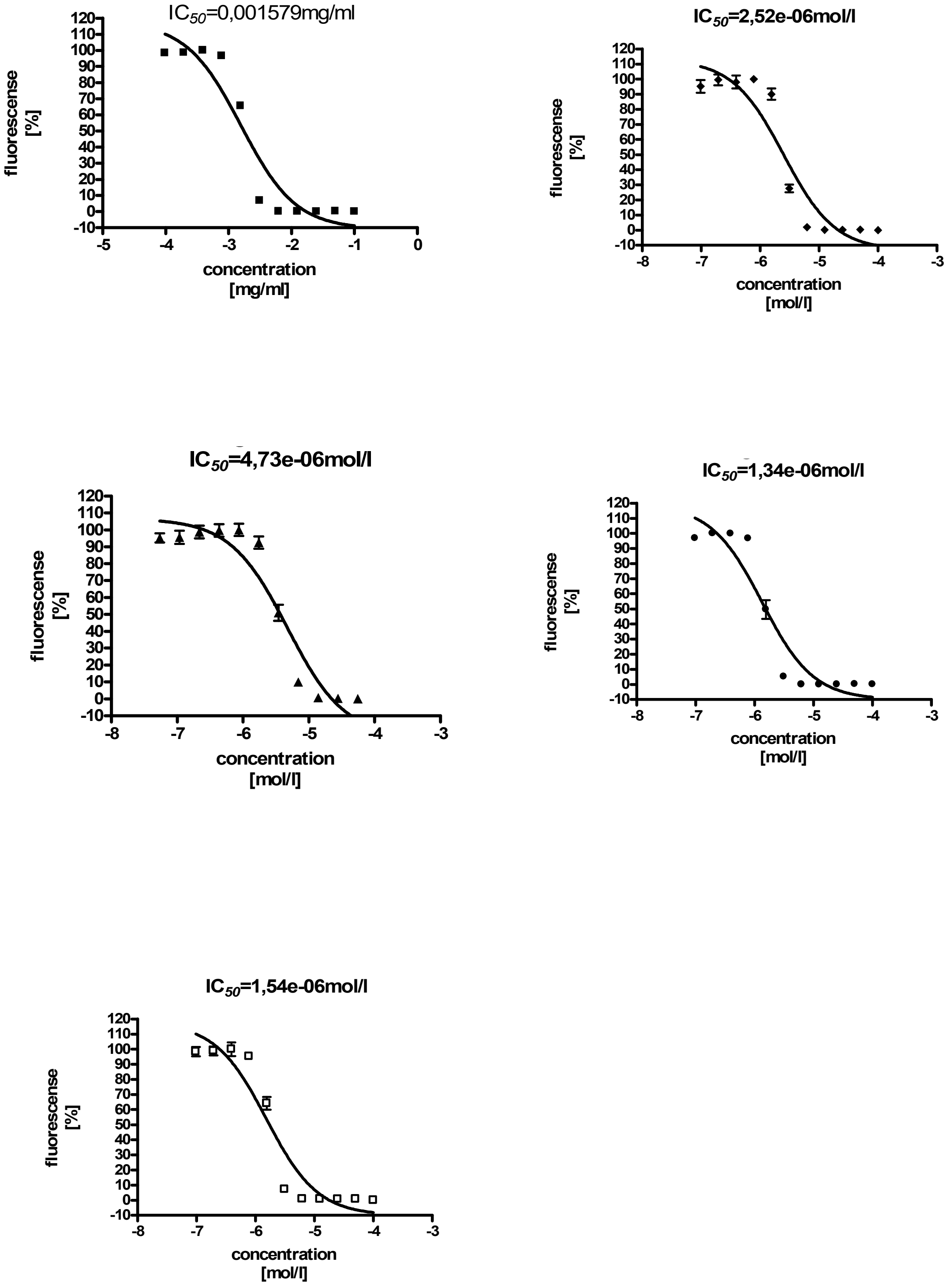

Figure 55: Dose-response curves

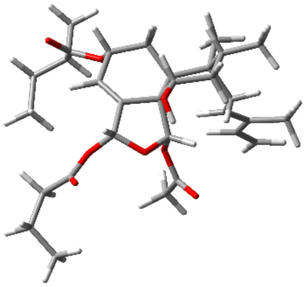

Conf A
18.4%

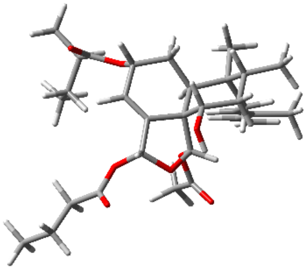

Conf B
18.1%

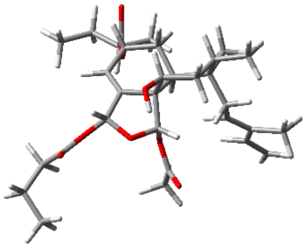

Conf C
17.2%

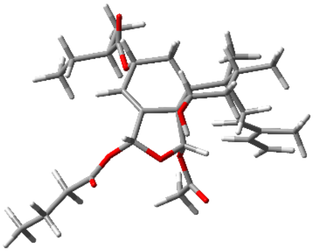

Conf D
16.9%

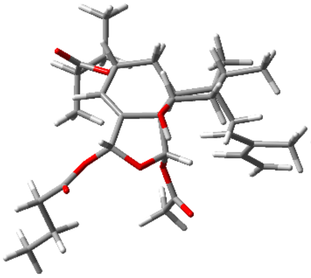

Conf E
7.6%

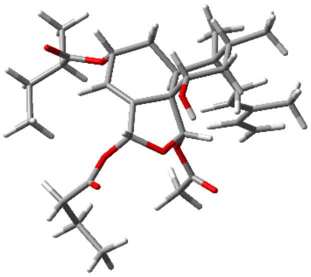

Conf F
7.4%

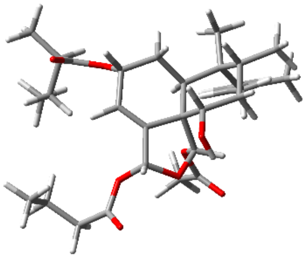

Conf G
4.0%

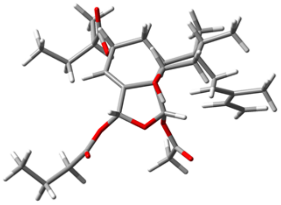

Conf H
3.9%

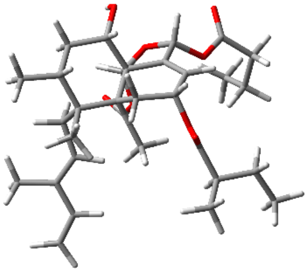

Conf I
3.4%

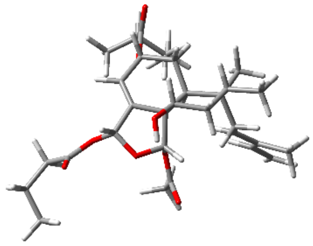

Conf J
3.1%

Figure 56: Low-energy conformers (≥ 1%) of compound **1** obtained at the B3LYP/6-311+G(d,p) IEFPCM/MeCN level of theory

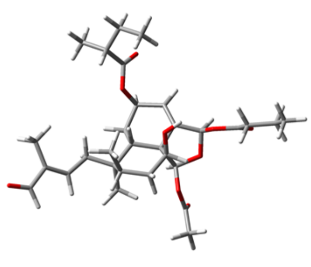

Conf A
81.7%

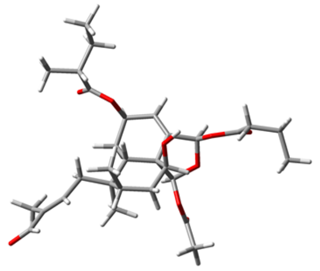

Conf B
15.3%

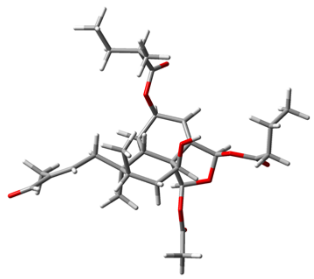

Conf C
3.1%

Figure 57: Low-energy conformers (≥ 1%) of compound **2** obtained at the B3LYP/6-311+G(d,p) IEFPCM/MeCN level of theory

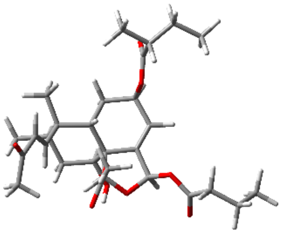

Conf A
17.7%

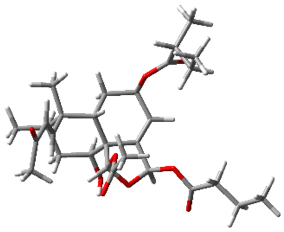

Conf B
15.6%

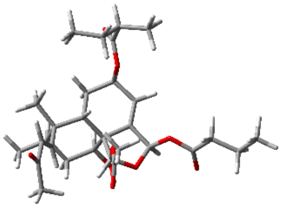

Conf C
12.4%

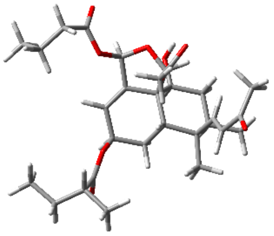

Conf D
7.7%

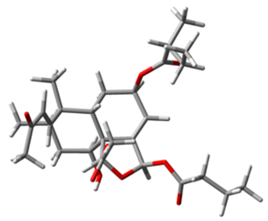

Conf E
7.5%

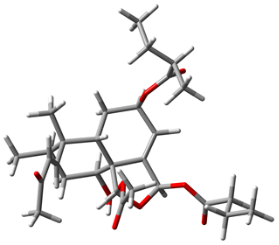

Conf F
6.9%

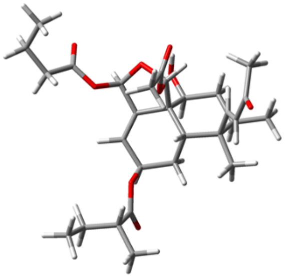

Conf G
6.7%

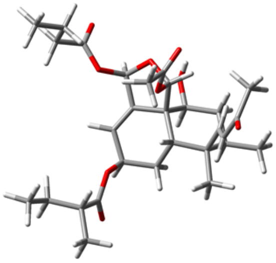

Conf H
6.2%

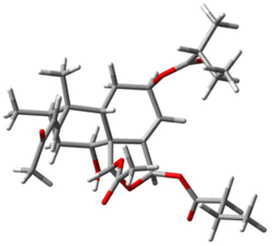

Conf I
6.1%

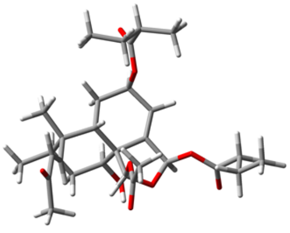

Conf J
4.8%

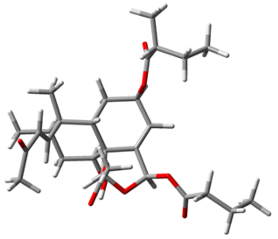

Conf K
3.7%

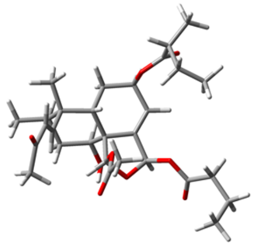

Conf L
3.1%

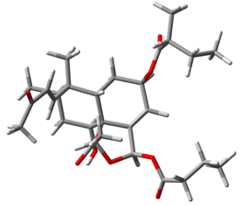

Conf M
1.6%

Figure 58: Low-energy conformers (≥ 1%) of compound **3** obtained at the B3LYP/6-311+G(d,p) IEFPCM/MeCN level of theory

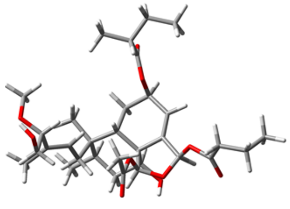

Conf A
46.6%

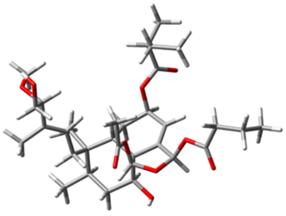

Conf B
10.0%

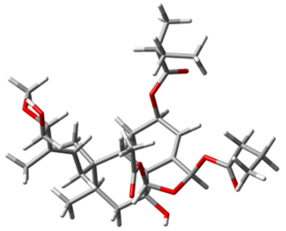

Conf C
9.7%

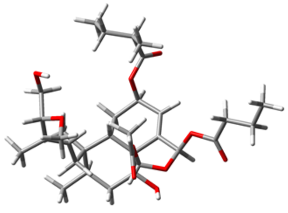

Conf D
7.8%

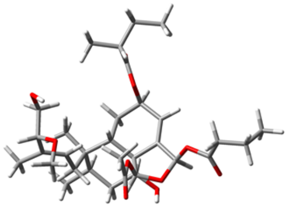

Conf E
7.0%

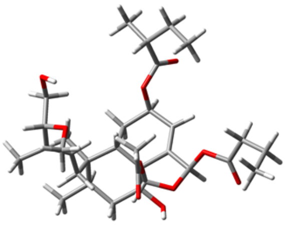

Conf F
6.9%

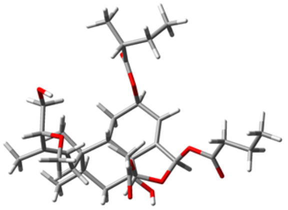

Conf G
3.0%

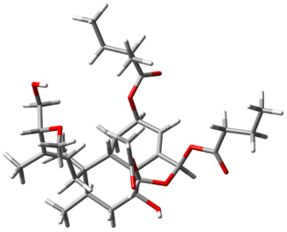

Conf H
2.9%

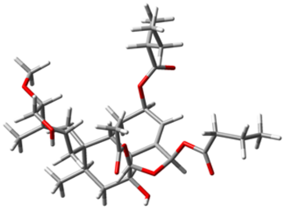

Conf I
1.8%

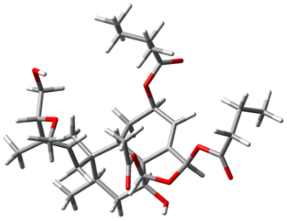

Conf J
1.8%

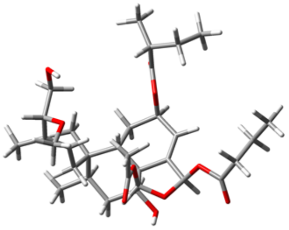

Conf K
1.3%

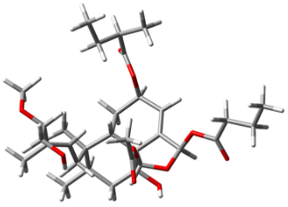

Conf L
1.2%

Figure 59: Low-energy conformers (≥ 1%) of compound **4** obtained at the B3LYP/6-311+G(d,p) IEFPCM/MeCN level of theory.

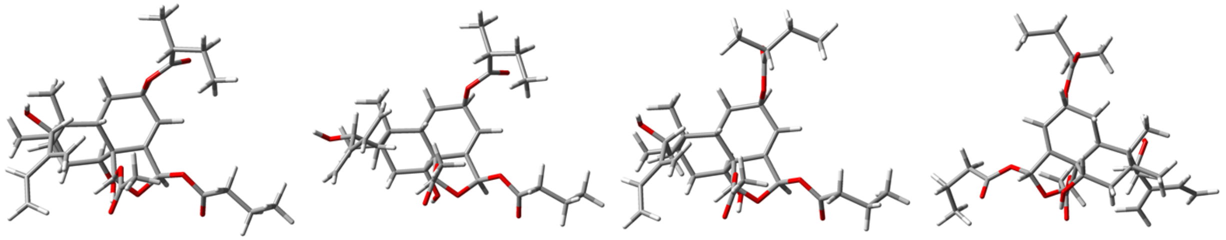

Conf A 45.8% | Conf B 29.4% | Conf C 23.2% | Conf D 1.6%

Figure 60: Low-energy conformers (≥ 1%) of compound **5** obtained at the B3LYP/6-311+G(d,p) IEFPCM/MeCN level of theory.

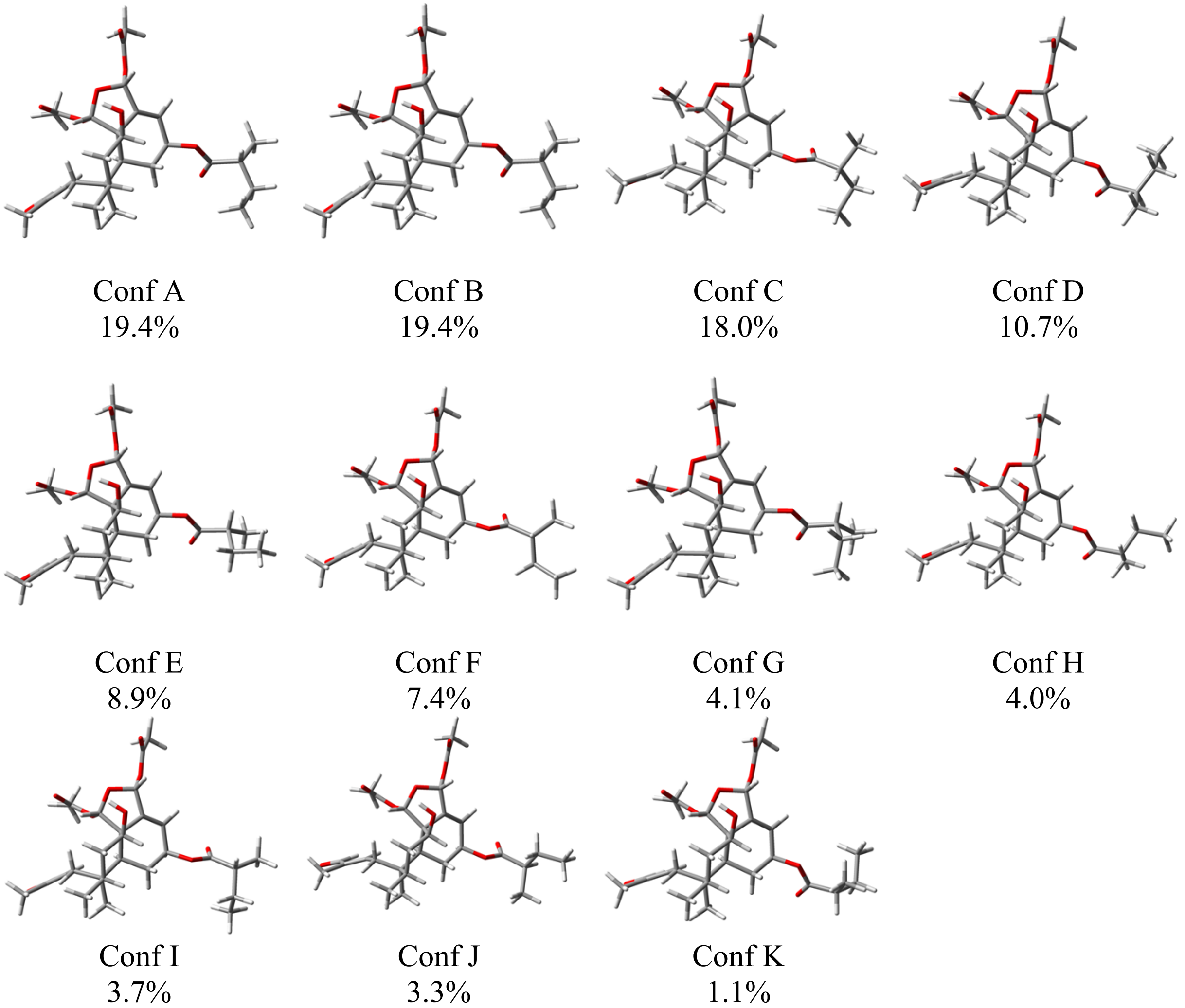

Figure 61: Low-energy conformers (≥ 1%) of compound **6** obtained at the B3LYP/6-311+G(d,p) IEFPCM/MeCN level of theory.

Reference

B. Sammet, T. Bogner, M. Nahrwold, C. Weiss, N. Sewald, *J. Org. Chem.*, 2010, **75**, 6953-6960.
